# Using neuroimaging genomics to investigate the evolution of human brain structure

**DOI:** 10.1073/pnas.2200638119

**Published:** 2022-09-26

**Authors:** Gökberk Alagöz, Barbara Molz, Else Eising, Dick Schijven, Clyde Francks, Jason L. Stein, Simon E. Fisher

**Affiliations:** ^a^Language and Genetics Department, Max Planck Institute for Psycholinguistics, 6500 AH Nijmegen, The Netherlands;; ^b^Donders Institute for Brain, Cognition and Behaviour, Radboud University, 6500 HB Nijmegen, The Netherlands;; ^c^Department of Human Genetics, Radboud University Medical Center, 6500 HB Nijmegen, The Netherlands;; ^d^Department of Genetics, University of North Carolina at Chapel Hill, Chapel Hill, NC 27599;; ^e^UNC Neuroscience Center, University of North Carolina at Chapel Hill, Chapel Hill, NC 27599

**Keywords:** neuroimaging, genetics, evolution

## Abstract

Alterations in brain size and organization represent some of the most distinctive changes in the emergence of our species. Yet, there is limited understanding of how genetic factors contributed to altered neuroanatomy during human evolution. Here, we analyze neuroimaging and genetic data from up to 30,000 people in the UK Biobank and integrate with genomic annotations for different aspects of human evolution, including those based on ancient DNA and comparative genomics. We show that previously reported signals of recent polygenic selection for cortical anatomy are not replicable in a more ancestrally homogeneous sample. We then investigate relationships between evolutionary annotations and common genetic variants shaping cortical surface area and white-matter connectivity for each hemisphere. Our analyses identify single-nucleotide polymorphism heritability enrichment in human-gained regulatory elements that are active in early brain development, affecting surface areas of several parts of the cortex, including left-hemispheric speech-associated regions. We also detect heritability depletion in genomic regions with Neanderthal ancestry for connectivity of the uncinate fasciculus; this is a white-matter tract involved in memory, language, and socioemotional processing with relevance to neuropsychiatric disorders. Finally, we show that common genetic loci associated with left-hemispheric pars triangularis surface area overlap with a human-gained enhancer and affect regulation of *ZIC4*, a gene implicated in neurogenesis. This work demonstrates how genomic investigations of present-day neuroanatomical variation can help shed light on the complexities of our evolutionary past.

## Significance

Which aspects of our genetic makeup contribute to distinctly human features of brain anatomy? Here, we identify associations between common DNA variants and interindividual differences in cortical circuitry, based on neuroimaging in tens of thousands of people, and assess how they relate to genomic regions of evolutionary interest, capturing different timescales along the lineage leading to *Homo sapiens*. Our work confirms and extends links between human-gained enhancers active in fetal brain tissue and cortical surface area measured in adults, including left-hemisphere regions related to speech. The study also reveals that regions of the genome carrying introgressed Neanderthal variants make a significantly diminished contribution to connectivity of the left-hemisphere uncinate fasciculus, a white-matter tract involved in mapping sound to meaning.

The size and organization of the brain exhibit great variation across primates, with especially notable differences in our own species (1). Comparative studies of ape neuroanatomy, along with endocranial fossil data from archaic hominins, indicate that there were dramatic expansions of cortical surface area on the lineage leading to *Homo sapiens*, as well as more recent shifts in brain shape (2–4). Today, the human cerebral cortex has a surface area of ∼1,843 ± 196 cm^2^ per hemisphere, compared to ∼599 cm^2^ per hemisphere for our closest living relative, the chimpanzee (1). It is thought that differences in brain size and shape were accompanied by altered architecture for a number of white-matter tracts (5). Given that changes in neuroanatomy coincided with the emergence of complex language and cognitive skills in humans (6), it has been suggested that an expanded cortex and/or associated alterations in connectivity and organization contributed to the evolution of complex behaviors (7–9). Nonetheless, despite advances across a range of disciplines, we still have only a limited understanding of the molecular bases of these fundamental aspects of human brain evolution.

A promising way to address this gap is through large-scale neuroimaging genomic investigations in present-day humans, integrating with the latest data from the ancient DNA field and broader literature on comparative primate genetics. Datasets containing brain MRI data and genetic information on single-nucleotide polymorphisms (SNPs) for tens of thousands of individuals, such as those collected by the Enhancing NeuroImaging Genetics through Meta-Analysis (ENIGMA) Consortium (10) and in population cohorts like the UK Biobank (11), have recently enabled the first well-powered genome-wide association studies (GWAS) of complex neuroanatomical traits. The last decade has also seen dramatic advances in ancient DNA research, including the discovery of putative Neanderthal introgressed alleles that can be found to varying degrees in the genomes of some present-day humans, resulting from admixture events ∼50,000 to 60,000 y ago (12). Such findings led to identification of long stretches of regions in the human genome that are significantly depleted of introgressed fragments—archaic deserts—with potential relevance for genetics of human-specific traits (13). On a deeper timescale, enhancer elements that gained activity in the human lineage were identified by comparing genome-wide posttranslational histone modification profiles across human, macaque, and mouse cortical brain tissue (14). Elements of this kind, referred to as human-gained enhancers (HGEs), can potentially shed light on the last ∼30 My of brain evolution (15). Here, we perform GWAS of structural neuroimaging measures in the UK Biobank (up to ∼30,000 participants) and use the results, together with evolutionary annotations of the genome capturing different timescales along the human lineage, to gain insights into regional expansions of cortical surface area as well as effects on brain connectivity.

Prior work by Tilot et al. (16) investigated the evolution of hemisphere-averaged cortical surface area using a neuroimaging genetics meta-analysis comprising results from 33,992 participants of European ancestry (23,909 from 49 cohorts participating in the ENIGMA consortium and 10,083 from the UK Biobank) (10). As well as directly using the available GWAS data to test for recent polygenic selection (i.e., involving effects at many different loci), the study took genomic regions with known relevance to human evolution and asked whether current genotypic variation in those regions contributes more than expected to interindividual differences in the neuroanatomical traits. There were two key findings: 1) evidence of recent polygenic selection based on singleton density scores (SDS) (17) for common variants associated with surface area and 2) enrichments of SNP-based heritability within HGEs active in fetal brain tissue, for a set of cortical regions. One potential confound acknowledged by Tilot et al. (16) stemmed from the multicohort structure and residual population stratification of the ENIGMA dataset used for GWAS, factors which can adversely affect evolutionary analyses. In particular, with regard to the polygenic selection signals, SDS analysis is known to be susceptible to residual population stratification (18, 19). SNP-based heritability estimates are also prone to confounding effects of population stratification in admixed samples to a certain extent (20, 21).

In the present study, we first performed a targeted replication analysis of the two main Tilot et al. (16) findings using a large independent cohort that is ancestrally homogenous (18,960 participants from UK Biobank, not included in the earlier analysis). The prior evolutionary investigations were limited by the use of measures averaged across hemispheres, but it is well established that certain cognitive specializations of our species, such as language, depend on lateralized circuits in the human brain. Thus, in the second stage of our study we went on to perform an analysis using genome-wide scans of 33 regional and global surface area measures for each hemisphere separately. We maximized power for this effort by using an even larger study sample (*n* = 30,332) from the UK Biobank, including a subset of individuals from the prior work on hemisphere-averaged measures (16), allowing us to investigate evolutionary annotations for genetic variants affecting surface area of left- and right-hemispheric regions. These analyses were enhanced over prior work by using updated sets of evolutionary annotations, including HGEs, Neanderthal introgressed alleles, and archaic deserts. In addition, we moved beyond surface area-based morphometrics to investigate brain connectivity. White-matter microstructure can be probed with diffusion MRI (dMRI) in vivo to explore structural connectivity patterns between different regions of the brain (22). Therefore, we leveraged fractional anisotropy (FA) values of 48 standard space white-matter tracts in UK Biobank (*n* = 29,924) to identify genetic variants associated with white-matter connectivity within and across hemispheres then assessed links to evolution across the same annotations as those used for cortical surface area. We demonstrated the value of integrating neuroimaging genomics with data on human-specific regulatory elements, gene expression, and evolutionary history for identifying genetic pathways of interest, using the *ZIC4* gene as an illustration. Finally, we investigated human accelerated regions (HARs) (23–27) and differentially methylated regions between anatomically modern humans and archaic hominins (referred to as AMH-derived DMRs) (28), assessing overlaps with GWAS signals from all full and regional surface area measures. HARs and AMH-derived DMRs are two important evolutionary annotations that could not be robustly investigated using the partitioned heritability approach due to low SNP coverage (*Materials and Methods*). We anticipate that shedding light on present-day variation and evolution of human cortical anatomy will enhance our understanding of disorders affecting brain and cognition (29, 30).

## Results

### Replication of Deep, but Not Recent, Evolutionary Findings for Hemisphere-Averaged Surface Area.

**Fig. 1.**
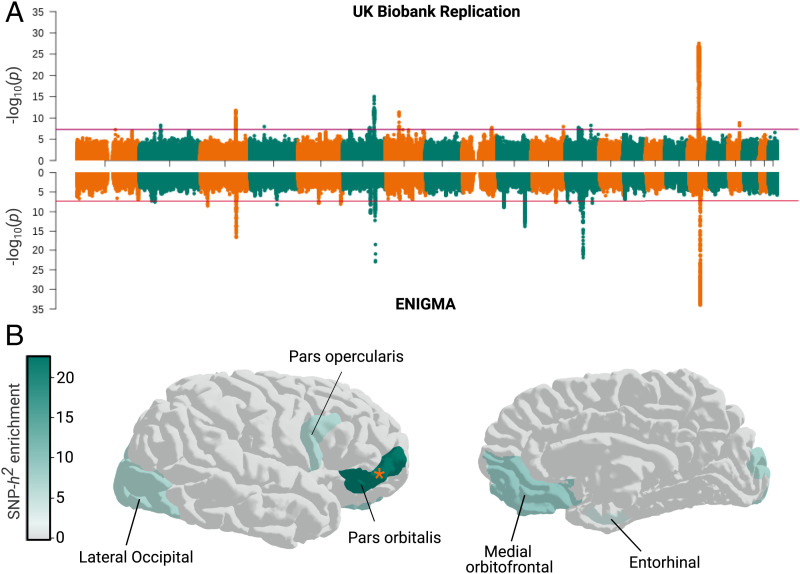
Replication of deep evolutionary findings for hemisphere-averaged surface area. (*A*) Manhattan plot of loci associated with total surface area in UK Biobank replication sample (*Top*, this study) and ENIGMA (*Bottom*) from Grasby et al. (10) GWAS. Red horizontal lines indicate genome-wide significant multiple-testing correction threshold. (*B*) SNP heritability of pars orbitalis is enriched in HGEs active at the seventh postconception week in fetal human brain, both in Tilot et al. (16) and our UK Biobank replication. SNP-heritability enrichments were estimated with LD-Score Regression. For this replication analysis, we tested only the five regions showing significance in the prior study, applying FDR correction accordingly. Regions that were not tested are in gray.

Toward replication of the main Tilot et al. (16) findings, we first identified genetic loci associated with interindividual variation in hemisphere-averaged cortical surface area using MRI and genome-wide genotype data of UK Biobank (accessed February 2020) in a set of individuals independent from those analyzed for the prior evolutionary study (16). In particular, to avoid any overlap with data contributing to the initial discovery GWAS by Grasby et al. (10), participants from UK Biobank neuroimaging releases prior to 2018 were excluded from this part of the study. Moreover, our replication effort focused on individuals of White British ancestry to limit potential population stratification. Anatomical measures were derived from structural MRI and the cortical regions were extracted from parcellations based on the Desikan–Killiany atlas (31). Applying an additive model, we conducted GWAS for total surface area as well as 33 cortical regions averaged across both hemispheres in 18,960 individuals (*SI Appendix*, Table S1). In line with previous work, common genetic variants accounted for 40.22% (SEM = 4%) of the phenotypic variation in total surface area (*SI Appendix*, Table S2). We observed very high genetic correlations between our independent UK Biobank subset of White British ancestry and the earlier GWAS data [global surface area r_g_(SEM) = 1.03(0.05), *P_r_*_G_ = 4.52 × 10^−91^] (*SI Appendix*, Fig. S1 and Table S3), suggesting consistent genetic underpinnings of cortical anatomy in Grasby et al. (10) and the independent sample that we analyzed here, as also illustrated by the similarity in Manhattan plots (Fig. 1*A*).

To further minimize potential confounding effects of residual population stratification on GWAS effect size estimates, we applied the ancestry regression procedure implemented by Tilot et al. (16). As in that study, we then analyzed the ancestry regressed-GWAS summary statistics using SDS (17), a method for detecting signs of polygenic selection acting over the past ∼2,000 to 3,000 y. First, we performed a targeted replication analysis for the full cortical surface and the eight cortical regions that had been reported as being under recent selection in the Tilot et al. study (16) and found no evidence of recent selection on the cortical area of any of these brain regions in this independent homogenous sample (*SI Appendix*, Table S4). Next, we correlated the SDS results of all brain regions with results from Tilot et al. (16) but found no significant correlation (*SI Appendix*, Fig. S2).

We went on to study the second major finding of Tilot et al. (16), concerning the role of genetic variants located in HGEs active in fetal brain (14). As in the prior study, we used LD-score regression (LDSC) partitioned heritability analysis (32), a method that can assess whether common variation in particular regions of the genome contributes disproportionately to the total SNP heritability of a trait. Specifically, we tested in our independent homogenous sample whether we could replicate the finding that HGEs active in fetal human brain at the seventh week postconception impact hemisphere-averaged cortical surface area. We robustly replicated the SNP-heritability enrichment signal for pars orbitalis [enrichment(SEM) = 22.82(8.02), *P_FDR_* = 0.004] (Fig. 1*B*). This brain region showed the strongest evidence for SNP-heritability enrichment in Tilot et al. (16) and is involved in aspects of language processing (33, 34). Here, it should be noted that both the current and previous studies controlled for global surface area when performing GWAS for different cortical regions. The consistency of the findings also confirmed the robustness of LDSC heritability partitioning as a tool for investigating evolutionary annotations.

### Hemispheric Surface Area–Associated SNPs Are Enriched in Fetal Brain HGEs, Including Regions Involved in Speech, Language, and Visual Processing.

Given that cognitive specializations of our species, such as language, depend on lateralized circuits in the cortex (35), we went on to perform GWAS of 33 regional and global surface area measures for each hemisphere separately (68 measures in total). Since this part of our work was toward novel evolutionary analyses that had not been run in the prior study, we here used all the available participants of European ancestry with genetic/neuroimaging data (*n* = 30,332) in the UK Biobank (*SI Appendix*, Table S5). GWAS results for cortical regions were controlled for the matching total hemispheric surface area and may thus be considered independent of the total measures. Common variation accounted for 39.02% (SEM = 3.14%) of total left-hemispheric and 39.37% (SEM = 3.21%) of total right-hemispheric surface area variation (*SI Appendix*, Table S6). Because our targeted follow-up of Tilot et al. (16) showed a lack of replicability for SDS results (elaborated on in *Discussion*), in line with other work showing that this polygenic selection method can be easily confounded by residual population stratification (18, 19), our new analyses focused only on the partitioned heritability approach (32). In addition, partitioned heritability allowed us to analyze all individuals of European ancestry (not just the White British subsample), increasing the statistical power of the GWAS.

To enhance reliability of the LDSC-based partitioned heritability approach, we curated refined sets of evolutionary annotations that encompass at least 1% of the well-imputed and quality-controlled 1000 Genomes Phase 3 reference panel SNPs (36). Further, we combined biologically related annotations and removed SNPs that overlapped in opposing annotations (i.e., in archaic deserts and Neanderthal introgressed alleles). Three distinct sets of annotations passed our quality control (*Materials and Methods*) and were taken forward for the evolutionary analysis: 1) genomic regions covering fetal brain HGEs active at 7th, 8.5th, and 12th postconception weeks, 2) Neanderthal introgressed alleles, and 3) archaic deserts.

**Fig. 2.**
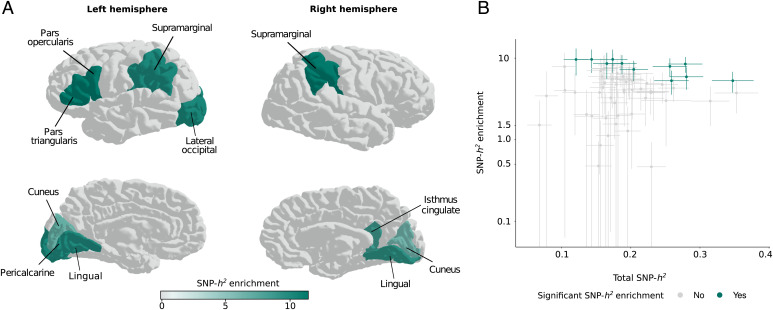
SNP heritability explained by genetic variants in HGEs active at 7th, 8.5th, and 12th postconception weeks in fetal brain reached significance for various regions in each hemisphere. (*A*) Brain plots showing SNP-heritability enrichment levels for left and right hemisphere regions that reached significance. (*B*) The relation of total SNP-heritability to SNP-heritability enrichment in HGEs. Data points represent regional hemispheric surface areas. Regions that showed significant heritability enrichment (*P_FDR_* < 0.05) are indicated in green and others in gray. FDR correction was applied for 43 independent cortical regions (*Materials and Methods*). Error bars represent SEs.

After false discovery rate (FDR) correction for 43 independent GWAS traits [estimated with PhenoSpD (37) using a genetic correlation matrix of the 68 cortical area traits], there were six left-hemisphere regions and four right-hemisphere regions that showed significant SNP-heritability enrichment in fetal brain HGEs (Fig. 2*A* and *SI Appendix*, Tables S7 and S8). Significant enrichments were identified for brain regions with average to high SNP-heritability estimates; it is possible that some positive findings may have been missed for brain regions with low SNP-heritability estimates due to lack of power (Fig. 2*B* and *SI Appendix*, Fig. S3). Left-hemisphere cortical regions that reached significance, pars triangularis [enrichment(SEM) = 9.65(3.04), *P_FDR_* = 0.015] and pars opercularis [enrichment(SEM) = 9.61(3.47), *P_FDR_* = 0.044], together form Broca’s area, well-known for its roles in speech-associated functions, among others (38). The other significant left-hemispheric regions are involved in functions such as word processing, visual processing, and object recognition (39). Right-hemispheric cortical regions that showed significant SNP-heritability enrichment in fetal brain HGEs have been linked to visual processing and language perception (40, 41). One region reached significance for heritability enrichment in fetal brain HGEs on both hemispheres: lingual gyrus, a region related to vision and word recognition (41). We did not find a significant enrichment or depletion for any surface area phenotype with respect to Neanderthal introgressed alleles. However, significant SNP-heritability depletion was seen within archaic deserts for total left- and right-hemispheric, as well as left-hemispheric pars opercularis, surface area [total left: enrichment(SEM) = 0.52(0.1), *P_FDR_* = 0.0006; total right: enrichment(SEM) = 0.57(0.11), *P_FDR_* = 0.005; left pars opercularis: enrichment(SEM) = 0.03(0.3), *P_FDR_* = 0.008] (*SI Appendix*, Fig. S4).

### Left-Hemispheric Uncinate Fasciculus Heritability Is Depleted in Neanderthal Introgressed Fragments.

We next moved beyond cortical surface areas to investigate measures of structural connectivity between different regions. Here, we leveraged FA metrics of 48 white-matter tracts in UK Biobank (*n* = 29,924) to identify genetic variants associated with white-matter integrity within and across hemispheres, using protocols that closely matched those used for our surface area GWASs (*SI Appendix*, Table S9). Similar to our findings for hemispheric surface area, white-matter connectivity GWAS identified large SNP-heritability levels for all 48 tracts (*SI Appendix*, Table S10), allowing assessment of links to evolution using LDSC partitioned heritability across the same annotations that we had used for cortical surface area. Genetic correlations (*SI Appendix*, Fig. S5 and Table S11) showed high consistency with relevant publicly available GWAS summary statistics obtained via the Oxford Brain Imaging Genetics Server (42).

**Fig. 3.**
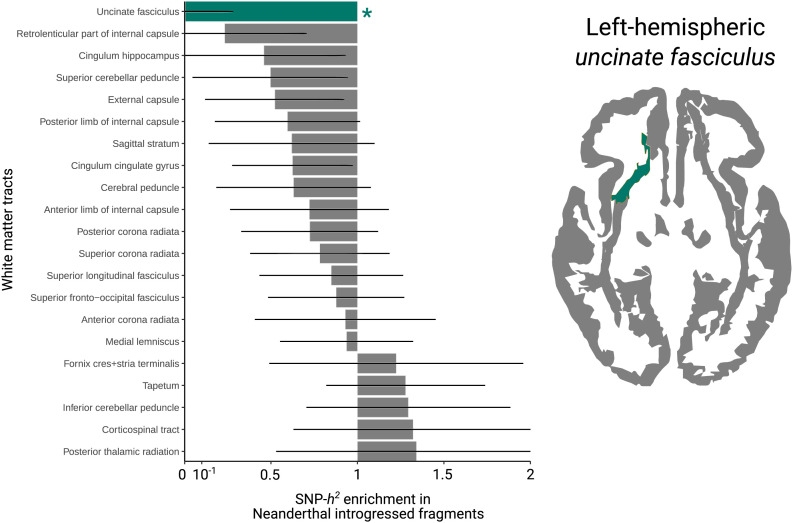
Strong SNP-heritability depletion of left-hemispheric uncinate fasciculus is in regions of Neanderthal introgression. Depletion and enrichment of heritability for left-hemispheric white-matter tracts. FDR correction was applied for 25 independent white-matter phenotypes (*Materials and Methods*). Left-hemispheric uncinate fasciculus is the only tract that was significant after FDR correction. *P < 0.05.

Our analysis detected a heritability depletion in Neanderthal introgressed alleles for left-hemispheric uncinate fasciculus [enrichment(SEM) = 0.01(0.28), *P_FDR_* = 0.022] (Fig. 3), after correction for 25 independent traits [estimated with PhenoSpD (37) using a genetic correlation matrix of the 48 tracts]. Although the tract is highly conserved, surface projections of the uncinate fasciculus show differences between humans and macaques (43), consistent with divergence of fascicular anatomy between Old World Monkeys and apes. No other significant enrichments or depletions were found for any of the white-matter tracts with the evolutionary annotations that we tested (*SI Appendix*, Table S12).

### Surface Area–Associated Loci in HGE Regions Impact Expression of the *ZIC4* Gene.

**Fig. 4.**
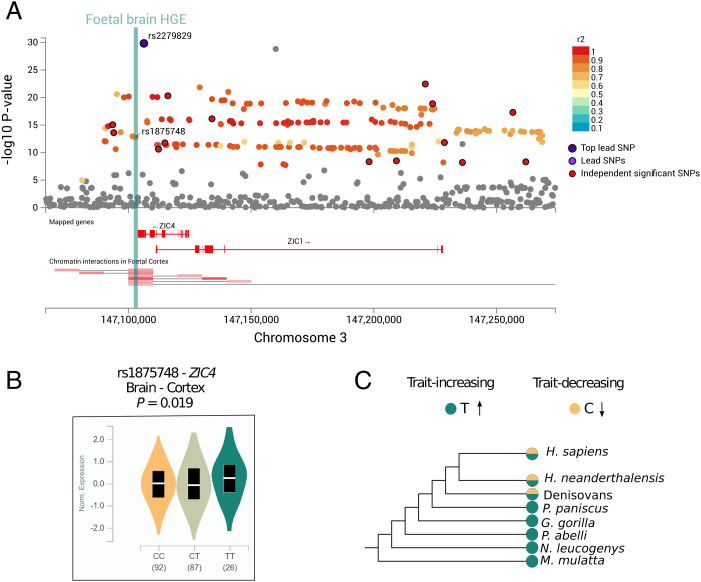
Functional and evolutionary annotation of the pars triangularis–associated *ZIC4* locus. (*A*) The LocusZoom plot (hg19) shows the SNPs in the locus genome-wide significantly (*P* < 5 × 10^−8^) associated with the pars triangularis that overlaps with the *ZIC4* and *ZIC1* genes. Colors represent linkage disequilibrium with the most significant SNP rs2279829 based on the 1000 Genomes Project reference data. A fetal brain HGE is located adjacent to *ZIC4* and contains the genome-wide significant SNP rs1875748. Chromatin interaction data from fetal brain that overlaps the locus and originates in the fetal brain HGE is shown below the LocusZoom plot from FUMA. (*B*) eQTL plot showing impact of rs1875748 on *ZIC4* expression in adult cortical brain tissue. Data and figures were taken from the GTEx database. (*C*) Ancestral and derived allele states in five nonhuman primates, Denisovans, Neanderthals, and *H. sapiens*. Effect directions of alleles are shown with arrows.

In the last part of the study, we zeroed in on loci of potential interest to more specifically evaluate the degree of convergence of trait, functional, and evolutionary associations. As an illustration, given the involvement of left-hemispheric pars triangularis in functions related to speech and semantic processing (44), as well as the significant heritability enrichment for fetal brain HGEs, we examined SNPs associated with this region in further detail. Among the 13 independent genome-wide significant (*P* < 5 × 10^−8^) left-hemispheric pars triangularis–associated loci (LD blocks harboring genome-wide significant SNPs that are in LD *r*^2^ > 0.6), there are two that overlap with a fetal brain HGE. This includes the chromosome-3 locus near *ZIC4,* which has the lowest *P* value for association with this brain region (rs2279829; *P* = 1.49 × 10^−30^, Beta = 17.37) and contains a genome-wide significant SNP (rs1875748; *P* = 1.54 × 10^−13^, Beta = 9.84) that falls in an HGE element located adjacent to *ZIC4* (chr3:147,101,875-147,103,850) (Fig. 4*A*). We annotated the locus with expression quantitative trait locus (eQTL) and chromatin interaction data, to further investigate functional consequences of the variants in this enhancer element. Chromatin interaction data from neural progenitor cells and fetal brain tissue indicated that the fetal brain HGE interacts with the promoter region of *ZIC4* and *ZIC1* (Fig. 4*A*). SNPs in the locus, including rs1875748, are annotated as *ZIC4* eQTLs in adult brain tissue, and there is evidence to suggest that the SNPs also function as *ZIC1* eQTLs (*SI Appendix*, Table S13). The minor allele (T) of rs1875748 is associated both with increased surface area of the left-hemispheric pars triangularis and with increased *ZIC4* expression in adult brain cortical tissue (Fig. 4*B*). Comparative genomics analysis indicated that this minor allele corresponds to the ancestral allele, and we detect the same polymorphism at the site in the available ancient DNA data from Neanderthals and Denisovans (Fig. 4*C*). The Human Genome Dating Atlas estimates rs1875748 polymorphism to be 47,675 generations old (95% confidence interval), corresponding to ∼1.2 Ma assuming 25 y per generation (45).

Given the association between the human-derived allele at rs1875748 and *ZIC4* expression in adult brain tissue, we further investigated the genetic architecture of the *ZIC4* locus (which also harbors the *ZIC1* gene). We looked for human-specific regional deletions and insertions in the last ∼6 My of human evolution by aligning human and chimpanzee sequences for a genomic locus flanking *ZIC4* 45 kb upstream and downstream (*SI Appendix*, Fig. S6) but found no major genomic rearrangements. Finally, we generated a multiple sequence alignment for *ZIC4* coding sequence across 36 mammalian species to run phylogenetic analysis by maximum likelihood (PAML) (46) to test for selection. Comparison between PAML site models for *ZIC4* coding sequence with the likelihood ratio test shows that 13.5% of the codons evolve under positive selection (χ^2^ *P* < 0.001) (*SI Appendix*, Table S14).

Finally, we applied our overlap analysis to HARs and AMH-derived DMRs (two interesting evolutionary annotations that could not be reliably investigated with partitioned heritability) using an extended list of genome-wide significant loci (*P* < 5 × 10^−8^ and SNPs that are in LD *r*^2^ > 0.6, comprising 2,596 loci in total) from all full and regional left- and right-hemisphere surface area GWASs. There were 10 loci coinciding with HARs and 23 loci located within AMH-derived DMRs (*SI Appendix*, Fig. S7 and Table S15). Intriguingly, 5 of the 10 loci that overlap with HARs are significantly associated with left or right hemisphere lateral orbitofrontal surface area, while loci coinciding with AMH-derived DMRs are associated with a range of cortical regions including superior parietal, lateral occipital, and pars orbitalis.

## Discussion

Recent availability of large-scale neuroimaging and genetics data in tens of thousands of individuals enabled us to investigate the evolution of complex neuroanatomical traits through common variation in a present-day population. By integrating genome-wide scans for cortical surface area and white-matter connectivity with relevant genomic annotations, we map current genetic variation with respect to annotations reflecting different aspects of human evolution, over diverse timescales.

The first part of our study attempted targeted replication of evolutionary findings from Tilot et al. (16) concerning hemisphere-averaged cortical surface area in a multisite sample that had combined many cohorts. We found that the signals of recent polygenic selection identified by Tilot et al. (16) could not be replicated in an independent ancestrally homogeneous cohort of a size similar to that in the earlier work. Our results suggest that, despite the use of an ancestry regression procedure, Tilot et al. (16) had not been able to fully exclude the effects of residual population stratification on GWAS effect size estimates, a known confound of the SDS method. Indeed, recent studies using this method have shown that polygenic selection signals for height based on SNPs below genome-wide significance are extremely sensitive to population stratification (18, 19). On the other hand, following the partitioned heritability approach of Tilot et al. (16) we could successfully replicate findings related to deeper evolutionary timescales. In particular, in our independent sample we robustly replicated the heritability enrichment signal for pars orbitalis surface area in fetal brain HGEs active at the seventh postconception week. Thus, the findings confirm the hypothesis that genetic variation in fetal brain HGEs can significantly impact neuroanatomical features as measured in living adults. Moreover, given the demonstrated replicability of the approach, we went on to apply partitioned heritability of evolutionary annotations to novel brain traits that had not been investigated in the previous work. The extreme sensitivity of the polygenic selection methods to residual population stratification stems from the fact that GWAS effect sizes are stratified across European populations. In contrast, Sohail et al. (19) suggested that LD-score regression is less likely to be affected by this issue, as residual stratification affects high- and low-LD SNPs to a similar extent, and so should not have a large impact on the parameter estimates. Sohail et al. also compared bivariate LD-score regression estimates from separate datasets to confirm its robustness.

Analyzing regions of evolutionary significance in genome screens of each hemisphere separately, we observed enriched heritability in fetal brain HGEs for surface area of left-hemispheric regions implicated in speech and language and of right-hemispheric regions involved in vision and self-projection, among others. Given the documented differences in inferior frontal gyrus anatomy in humans and macaques (47), and the importance of this part of the brain for speech and language in health and disease, the enrichment of genetic variants in human-specific enhancer elements is intriguing. In addition, findings showing heritability enrichment of visual-cortical regions in the same regulatory elements are consistent with the existence of substantial differences in the organization of primary visual cortex between humans and nonhuman primates (48). Moving beyond partitioned heritability, our work demonstrates how convergence of neuroimaging genomics with evolutionary data and information on gene function can shed light on contributions of potential loci of interest. As an example, we zeroed in on a locus on chromosome 3, using eQTL data to show that the same variants that are associated with the anatomy of the pars triangularis affect the neural expression of *ZIC4*. Of note, one of the eQTLs is located in the fetal brain HGE adjacent to *ZIC4*. Our analyses indicate that the *ZIC4*/*ZIC1* promoter region interacts with this enhancer element, suggesting further relevance for these two genes in cortical evolution, and we additionally show that the *ZIC4* coding sequence has been under positive selection in mammals. Both *ZIC4* and its colocalized paralog *ZIC1* are known to be involved in neurogenesis (49). Overlap analysis between GWAS signals from all full and regional surface-area GWASs and two evolutionary annotations (HARs and AMH-derived DMRs) pointed to a set of putative evolutionarily interesting loci and genes which can be candidates for further research in this domain. We note that this overlap analysis is a qualitative approach that does not involve evaluation of statistical significance but rather generates hypotheses for future targeted investigations of loci of interest.

Lack of significant heritability enrichments for cortical surface area within Neanderthal introgressed alleles and archaic deserts may suggest that variants influencing these traits tend to concentrate at genomic regions shaped in earlier periods of human evolution, consistent with the more prominent neuroanatomical differences between *H. sapiens* and nonhuman primates compared to Neanderthals. Nonetheless, we observed a significant heritability depletion for total surface area and left-hemispheric pars opercularis in archaic deserts, findings that are difficult to interpret. The depletion does not indicate an overall lack of common variants in the archaic deserts, as heritability partitioning is performed per variant, and all annotations contain at least 1% of SNPs in the reference panel. Archaic deserts are known to be enriched for genes expressed in certain brain regions including developing cortex and adult striatum (13), as well as for enhancer regions active in brain tissue (50). Neanderthal introgressed alleles in these brain enhancers have been subject to unusually stringent purifying selection (50). Thus, we hypothesize that the significant SNP-heritability depletion for full and regional cortical surface areas in archaic deserts could be a result of the strong purifying selection on the Neanderthal introgressed variants in brain enhancers. In other words, Neanderthal introgressed alleles might have initially replaced key variants in brain enhancers for brain structure, and the subsequent negative selection on these enhancers might have resulted in the identified depletion of SNP heritability observed in current living populations. Another explanation would be that archaic deserts coincide with regions of the genome that lack genes and regulatory elements involved in the early neurodevelopmental processes that establish cortical surface area. While it is known that archaic deserts are enriched for regulatory regions active in the brain, these may conceivably affect other aspects of neural structure/function, or different developmental periods.

Analyzing white-matter measures, we identified another relationship with ancient DNA data—a substantial SNP-heritability depletion within the Neanderthal introgressed alleles annotation, for connectivity of the left-hemispheric uncinate fasciculus. In other words, Neanderthal alleles that were introduced into the human genome through ancient admixture events and that have persisted to the present-day explain substantially less variability in uncinate fasciculus structure than expected under the null hypothesis of complete polygenicity, perhaps in relation to conservation of *H. sapiens*–specific features of neuroanatomy. A caveat of our dMRI based analysis is that we were not able to here investigate all tracts of potential evolutionary interest in the human brain. In particular, white-matter connectivity data for the arcuate fasciculus, a tract with especially compelling links to language processing and well-documented differences in comparative primate neuroanatomy, are not available in the UK Biobank and so could not be included in the present study. It has been hypothesized that language processing involves two streams; the dorsal stream connects Broca’s and Wernicke’s regions via the arcuate fasciculus and is involved in mapping sound to articulation, whereas the ventral language stream connects the anterior and lateral temporal lobe via the uncinate fasciculus and is involved in mapping sound to meaning (51). On the other hand, it is also known that the uncinate is highly conserved across humans and macaques (40). Thus, despite its critical role in the so-called language-relevant connectome (52), the evolutionary relevance of our heritability depletion finding for this trait is unclear. However, we note that heritability depletion in Neanderthal introgressed fragments has been observed for a number of other complex human traits (53). In addition to its involvement in language, the uncinate fasciculus has been linked to cognitive functions such as episodic memory, and aspects of socioemotional processing, and implicated in a range of neurodevelopmental and psychiatric disorders (54).

Notably, a lack of fossilized brain tissue from archaic hominins means that most prior knowledge of the neuroanatomical specializations of our species concerns gross levels such as overall brain size and shape. Hence, for the main part of this project it was necessary to adopt a systematic objective screening approach that considered all the regions of cortex and white-matter tracts available, yielding a higher multiple-testing burden and a consequent reduction in power. There are several other limitations of our study. First, for data availability reasons, the results were all derived from a population with European ancestry. Therefore, the scope of work is necessarily restricted to investigation of detectable polymorphic sites in a limited population. To consolidate findings, further research will be needed using neuroimaging genetics datasets from populations with diverse ancestral backgrounds, once sufficiently large-scale cohorts have been assembled. Second, it is likely that many of the critical genetic changes that affected key aspects of cortical anatomy in the course of hominin evolution have become fixed in all living humans and so cannot be directly studied in GWAS data from extant populations. Here, we capture the existing genetic and phenotypic variation in the UK Biobank and assess contributions of different evolutionary genomic annotations to heritabilities of neuroanatomical traits, as an indirect way to probe the relevance of those annotations for different aspects of human brain structure. Third, the relationship between heritability and evolution is far from straightforward, especially in light of the complex history of archaic hominins, involving multiple admixture events. Contributions of common genetic variants to heritability are shaped by selection and demography, and heritable variation is maintained by mutation–selection balance (55). Combining our approach with additional ancient DNA data that robustly track allele frequencies over the last ∼40,000 y or so could be valuable for enhancing analyses of the impacts of Neanderthal introgression. Fourth, although we used the largest homogenous sample currently available, we acknowledge that for those neuroimaging traits with modest SNP-based heritability estimates, larger GWAS sample sizes may be required to detect enrichments and/or depletions. As indicated by the fairly large error bars of the partitioned heritability results and varying levels of total SNP-heritability per trait, a larger GWAS sample size would have potential to reveal further enrichment and/or depletion signals, especially for the traits with lower SNP-heritability estimates. Although we are confident that we have been able to detect true heritability enrichment and depletion signals using the current cohort, it remains likely that the power was not sufficient to capture all the positive signals. Finally, there are other annotations of the human genome that are of interest for understanding cortical evolution beyond those included in the current study. However, given that such annotations are related to particular evolutionary events (e.g., selective sweeps, interspecific differences in chromatin accessibility), they often cover a relatively small number of SNPs, which complicates analyses based on SNP heritability. Thus, development of alternative methods beyond heritability partitioning will be important to derive more comprehensive accounts of the genetic bases of human brain evolution.

Overall, through analyses of large-scale neuroimaging genetics data, we showed that patterns of common variation in HGE elements, Neanderthal introgressed alleles, and archaic deserts influence the surface area and white-matter connectivity of our brains at global, regional, hemispheric, and microstructural levels in complex ways. Applying similar approaches to other measures of human brain structure and function, and in further large samples, holds promise for gaining further insights into evolution of our species.

## Materials and Methods

### Dataset.

All data for neuroimaging genetics were obtained from the UK Biobank under the research application 16066 with C.F. as the principal investigator. The present study involved two samples using surface-based morphometric data, further referred to as replication and hemisphere-specific sample, and one dMRI sample. Data availability and sample-specific preprocessing and quality control are described in detail in *SI Appendix*. Informed consent was obtained for all participants by UK Biobank with details about data collection and ethical procedures described elsewhere (56, 57).

### Neuroimaging Phenotypes.

This study made use of imaging-derived phenotypes generated by an imaging-processing pipeline developed and run on behalf of the UK Biobank (58, 59). We used imaging-derived structural measures (UK Biobank category 192) where cortical surface was parcellated for each individual according to the Desikan–Killiany atlas (31) and a standard preprocessed dMRI measure (UK Biobank category 134). All relevant preprocessing steps are described in full in the UK Biobank imaging documentation (https://biobank.ctsu.ox.ac.uk/crystal/crystal/docs/brain_mri.pdf).

### Genome-Wide Association Analysis.

For all individuals, imputed variant genotype data (UK Biobank Category 263, bgen files; imputed data v3, released March 2018) were extracted and variant-level quality control and SNP statistics were computed for each dataset using qctool v2.0.2 (https://www.well.ox.ac.uk/~gav/qctool_v2/). In each of the three study samples, variants with a minor allele frequency <0.1%, Hardy–Weinberg equilibrium *P* value <1 × 10^−6^, imputation quality INFO score <0.7 (included in the imputed UK Biobank data) and multiallelic SNPs were excluded.

This procedure resulted in 14,537,705 biallelic variants in the replication sample, 14,532,493 in the hemisphere-specific sample, and 14,531,866 in the dMRI sample. For the replication sample, following the approach of Tilot et al. (16) and Grasby et al. (10), univariate association analyses were conducted for total surface area and 33 cortical regions, where both metrics were averaged across hemispheres, resulting in 34 traits in total. For the hemisphere-specific sample, these same traits were analyzed separately for each hemisphere, resulting in 68 traits in total. For the dMRI sample, FA values for all 48 standard-space tracts were used as traits. Associations of phenotypes with sample-specific imputed genotype dosages were tested applying an additive model in BGENIE (v1.2) (56).

### Estimating Number of Independent Neuroimaging Traits.

For the hemisphere-specific surfaced-based and the dMRI analysis streams we accommodated the multiple-testing burden while considering the correlation structure of the traits under investigation. In each case, we used spectral decomposition of matrices (SpD) implemented in the R package PhenoSpD (37) to calculate the effective number of independent variables (VeffLi), resulting in 43.07 and 25.34 independent traits for hemisphere-specific and dMRI samples, respectively.

### Recent Polygenic Selection Replication Analysis.

For the hemisphere-averaged replication sample, to most closely match the Tilot et al. (16) study design we first assessed the impact of population stratification on effect sizes of GWAS summary statistics by applying a block jackknife correlation test between the top 20 ancestry principal components (PCs) and GWAS beta values. We then applied the ancestry regression method as implemented in Tilot et al. (16). Summary statistics of each hemisphere-averaged GWAS were fitted to the top 20 ancestry PCs using a regression model. The LDSC (60) intercept for each set of summary statistics was estimated as an additional measure of population stratification, following the guidelines given in the LDSC website (https://github.com/bulik/ldsc/wiki/LD-Score-Estimation-Tutorial). SDS (17) analysis as implemented in Tilot et al. (16) was then applied, following instructions in https://bitbucket.org/jasonlouisstein/enigmaevolma6/src/master. We first downloaded SDS values per genomic variant from https://datadryad.org/stash/dataset/doi:10.5061/dryad.kd58f and merged with ancestry-regressed summary statistics by SNP IDs, while SNPs lacking an SDS value were removed from the analysis. In case of mismatching alleles, the SDS values were flipped to match the correct allele. Variants in merged files were sorted based on genomic position, and a block jackknife Spearman’s correlation test (100 blocks) between trait-increasing SDS values and GWAS Z-scores was applied. This analysis was applied for 33 cortical regions segmented with FreeSurfer (22) and full cortical surface area. In this case, to match the evaluation procedure adopted by Tilot et al. (16), *P* values were conservatively corrected for multiple testing (without taking into account nonindependence of traits) using Benjamini–Hochberg FDR (61). We assessed consistency with the Tilot et al. findings in two stages: 1) a targeted replication of only the nine significant traits from the prior report and 2) correlation analysis of all 34 traits across the two studies.

### Partitioned Heritability Analysis with Custom Evolutionary Annotations.

Surface area SNP-heritability contributions of each evolutionary annotation were computed using LDSC partitioned heritability analysis (32) following the guidelines in the Wiki page (https://github.com/bulik/ldsc/wiki/Partitioned-Heritability). For the replication study, proportion of heritability, heritability enrichment, and SNP proportions within fetal cortical HGEs active at the seventh postconception week (14) were computed. Fetal HGEs annotation was controlled for both the baselineLD model v2.2 and fetal brain active regulatory elements from the Roadmap Epigenomics Consortium database (62). Before partitioning heritabilities for hemispheric surface area and white-matter tract SNP-heritabilities, three newly curated enhanced evolutionary annotations were established. We made sure that these three annotations cover at least 1% of the total number of well-imputed SNPs in 1000 Genomes Phase 3 reference panel (only HapMap3 SNPs without the MHC region) (https://github.com/bulik/ldsc) to enhance reliability of heritability estimates. For HGEs, we merged HGEs active in human fetal cortical tissue (14) at three consecutive developmental stages adopted by Tilot et al. (16), creating a single annotation covering all fetal cortical HGEs, increasing the SNP proportion of fetal brain enhancers annotation to above 1%. For Neanderthal introgressed alleles (63), we made use of a list covering Neanderthal introgressed SNPs and SNPs in perfect LD with them (*r*^2^ = 1). The archaic deserts (13) annotation was also refined, by removing Neanderthal introgressed SNPs (12) within this annotation. Vernot et al. (13) used a sliding window approach to describe genomic regions that are significantly depleted of Neanderthal sequences in Europeans, East Asians, South Asians, and Melanesians, with average introgression percent <10^−3.5^. This analysis yielded six genomic regions that are significantly depleted for Neanderthal sequence, spanning 85.3 Mb in total. We removed 128 Neanderthal introgressed SNPs in these desert regions, corresponding to 10^−4^ of the total length of Neanderthal deserts, and removed the haplotypes that these introgressed SNPs were in LD with (*r*^2^ > 0.6). HGEs active in fetal cortical tissue, archaic deserts and Neanderthal introgressed alleles were controlled for the baselineLD v2.2 model provided by the Alkes Price group (32).

### Functional and Evolutionary Annotation of Associated Loci.

The GWAS results for left pars triangularis were annotated using Functional Mapping and Annotation of Genome-Wide Association Studies (FUMA; https://fuma.ctglab.nl; version 1.3.6a) (64). Using the SNP2GENE function, genome-wide significant loci were annotated with eQTL data from four databases with gene expression data of fetal and adult brain samples: GTEx V8 (brain samples only; www.gtexportal.org), BRAINEAC (www.braineac.org), CommonMind consortium (65), and psychENCODE (66). Loci were also annotated with chromatin interaction data of fetal and adult brain samples from three sources: psychENCODE, Giusti-Rodriguez et al. (67) and Schmitt et al. (68). GTEx V8 data assessed through the GTEx Portal (https://www.gtexportal.org) was used to visualize the association between rs1875748 and *ZIC4* expression in adult cortical brain tissue. Neanderthal and Denisovan allele states for SNPs of interest were derived from the ancient DNA genotype data provided by the Max Planck Institute for Evolutionary Anthropology (cdna.eva.mpg.de/neandertal/) (69). Sequence alignment for the *ZIC4* locus was performed using sequence data from GRCh37 and panTro3, DOTTER (70) for alignment and plotting. Multiple sequence alignment of 36 mammalian species was downloaded from the Ensembl phylogenetic context server (71). We used the codeml program from the PAML package (46) to detect selection on the *ZIC4* coding sequence. To identify genome-wide significant loci associated with any of our neuroimaging traits, we performed clumping with PLINK (72), and selected SNPs that are in LD (*r*^2^ > 0.6) with clumped GWAS SNPs. We then identified the loci that overlap with HAR or AMH-derived DMRs by using the findOverlaps function from the GenomicRanges R package (73).

### Data Analysis and Visualization.

Genetic and evolutionary analyses were conducted on the computing cluster of the Max Planck Institute for Psycholinguistics. Results were parsed and organized with bash scripts. Statistical analyses were conducted with R, Python, and Linux Shell. Plots were generated using R packages plotly and ggplot2.

## Supplementary Material

Supplementary File

## Data Availability

Neuroimaging and genotype data used for GWAS are available from UK Biobank (https://www.ukbiobank.ac.uk). GWAS summary statistics for hemispheric and hemisphere-averaged surface area metrics, as well as for dMRI metrics, are deposited at The Language Archive, a public data archive hosted by the Max Planck Institute for Psycholinguistics (https://archive.mpi.nl/mpi/islandora/object/mpi:1839_4f0e197a_d3cc_4bf7_a5ef_dbe956f59691?asOfDateTime=2022-05-25T12:03:02.720Z) (74). All scripts used for the analysis are available on the project GitHub repository (https://github.com/galagoz/cortical-evo) (75). Previously published data were used for this work (10, 16, 42).
