# Supplementary material for: Using neuroimaging genomics to investigate the evolution of human brain structure
Source: Proc Natl Acad Sci U S A. 2022 Sep 26;119(40):e2200638119. doi: 10.1073/pnas.2200638119 (PMC9546597; doi:10.1073/pnas.2200638119)
Supplement: Supplementary File [file pnas.2200638119.sapp.pdf]

PNAS
www.pnas.org

Supplementary Information for

Using neuroimaging genomics to investigate the evolution of human brain structure

Gökberk Alagöz[a], Barbara Molz[a], Else Eising[a], Dick Schijven[a], Clyde Francks[a,b,c], Jason L. Stein[d,e], Simon E. Fisher[a,b,1]

Simon E. Fisher

Email: Simon.Fisher@mpi.nl

This PDF file includes:

Supplementary Information Text
Figures S1 to S7
Tables S1 to S15
SI References

Supplementary Information Text

Genetic quality control

Participants with mismatch of their self-reported (UK Biobank data field 31) and genetically inferred sex (UK Biobank field 22001) were excluded, as were those with putative aneuploidies (UK Biobank field 22019) or who were identified as outliers based on heterozygosity (PC corrected heterozygosity >0.1903) or genotype missingness rate (missing rate >0.05) (UK Biobank field 22027) (1). As explained in the main manuscript, the replication sample was restricted to participants of 'White British' ancestry (UK Biobank data field 22006) (1), while for the hemisphere-specific and dMRI samples participants with 'White European' and similar genetic ancestry were selected. Therefore, principal components (PCs) were calculated for all participants passing above described sample level quality control. Further, all participants reporting White European ancestry (data field 21000) were selected and a Bayesian outlier detection algorithm (aberrant) (2) was run to identify dense clusters of participants with similar genetic ancestry along PC1-PC2, PC3-PC4 and PC5-PC6. Participants included in the intersection of all three clusters were selected.

For all cohorts we included only unrelated individuals. Therefore, pairs of individuals with a kinship coefficient > 0.0442 (UK Biobank data field 22021) were identified. Listed individuals who passed the above described QC with available imaging data were extracted from a pre-computed kinship table (1), and one individual from each pair was excluded. Exclusion of individuals was prioritized if they were related to a larger number of other participants.

Neuroimaging phenotypes.

All details regarding image acquisition and subsequent applied processing pipelines are available on the UK Biobank website (http://biobank.ctsu.ox.ac.uk/crystal/refer.cgi?id=2367), with the respective brain imaging documentation (http://biobank.ctsu.ox.ac.uk/crystal/refer.cgi?id=1977), and are described in detail in Miller et al (3). In brief, for surface-based morphometry, T1-weighted MRI images were used for surface reconstruction with FreeSurfer 6.0 'recon all', where surface area is measured at the grey–white matter boundary, and thickness is measured as the average distance between the white-matter and pial surfaces (4, 5). Derived output was quality controlled according to UK Biobank standards described in the imaging documentation. We excluded participants whose T1 scan was deemed unusable after quality control (QC) by UK Biobank (6) and included only participants where a T2_Flair scan was used in conjunction with a T1-weighted MRI scan for FreeSurfer processing as indicated by Data field 26500, to avoid introducing any procedural bias. For both data sets also participants with neurological brain disorders as indicated by both medical records (Data field 41202 – 42105) and self-reported medical conditions in the verbal interview (Data field 20002, instance 0-2) were excluded. For the replication dataset, participants included in the data release prior to April 2018 were excluded to avoid any sample overlap with the earlier study by Tilot et al. (7, 8). For each participant, we used the provided bilateral measures for global surface area as well as 33 cortical regions parcellated using a gyrally defined atlas (9). For phenotype-specific QC, participants whose global measures extended -/+ 5* median absolute deviation (MAD) were excluded.

For hemisphere-averaged surface-based morphometric data this resulted in a total of 18,960 participants for the replication sample with an age range of 47-81 years (median=65), where 10,071 participants were female and 8,889 were male, while the hemisphere-specific sample included a total of 30,322 participants with an age range of 45-81 years (median=64) where 16,120 participants were female and 14,212 participants were male. Regional specific datapoints exceeding -/+ 5* MAD were removed bilaterally for the specific region. Sample sizes differed depending on the specific cortical regions, and are listed in *SI Appendix*, Tables S1 and S2. The dMRI data were acquired using a multishell approach with two diffusion weightings (b = 1 and 2 ms/μm2) where for each diffusion-weighted shell a total of 50 non-coplanar diffusion-encoding directions were acquired. The generated data were quality controlled according to the UK Biobank imaging pipeline. Further, the b=1000 shell was fed into the diffusion-tensor-imaging (DTI) fitting tool DTIFIT, generating the fractional anisotropy output used in Tract-Based Spatial Statistics (TBSS) processing (10). Here, the FA image is aligned to a standard-space white-matter skeleton. As before, we excluded participants whose T1 scans were deemed unusable after QC by UK Biobank (11). For each participant, we used the provided averaged FA measures within 48 standard-space tracts as defined by the JHU White-Matter Atlas (12, 13). Again, participants whose measures extended -/+ 5*median absolute deviation (MAD) were excluded, thus sample sizes differed depending on the specific tract and are listed in *SI Appendix*, Table S9.

Genome wide association analysis.

We included standard covariates in all our association analyses, namely age (UK Biobank field 21003-2.0), age^2, sex (UK Biobank field 31-0.0), sex-by-age and age^2 interactions, the first 10 genetic principal components (UK Biobank fields 22009-0.1 to 22009-0.10) and dummy variables for assessment centre (UK Biobank field 54-2.0) and genotype measurement array (UK Biobank field 22000-0.0). For all surface-based morphometric traits we additionally included scanner position parameters (X, Y and Z position: fields 25756-2.0, 25757-2.0 and 25758-2.0), T1 signal-to-noise ratio (UK Biobank field 25734-2.0) and T1 contrast-to-noise ratio (UK Biobank field 25735-2.0). Further, for all surface-based regional measures, global measure of surface area (either averaged or hemisphere-specific depending on data sample) was included as an additional covariate to assure identification of genetic influences specific to each region. Manhattan plots and QQ-plots were made using the “qqman” R (14) package (v0.1.8). Subsequently, LD score regression (15) was used to determine SNP-based heritability for all traits investigated and genetic correlation of summary statistics derived from the replication sample with the respective (averaged) traits in (8), as well as genetic correlations between summary statistics for all dMRI traits with the respective publicly available GWAS summary statistics obtained via the Oxford Brain Imaging Genetics Server (http://big.stats.ox.ac.uk/) (10).

Supplemental Figures

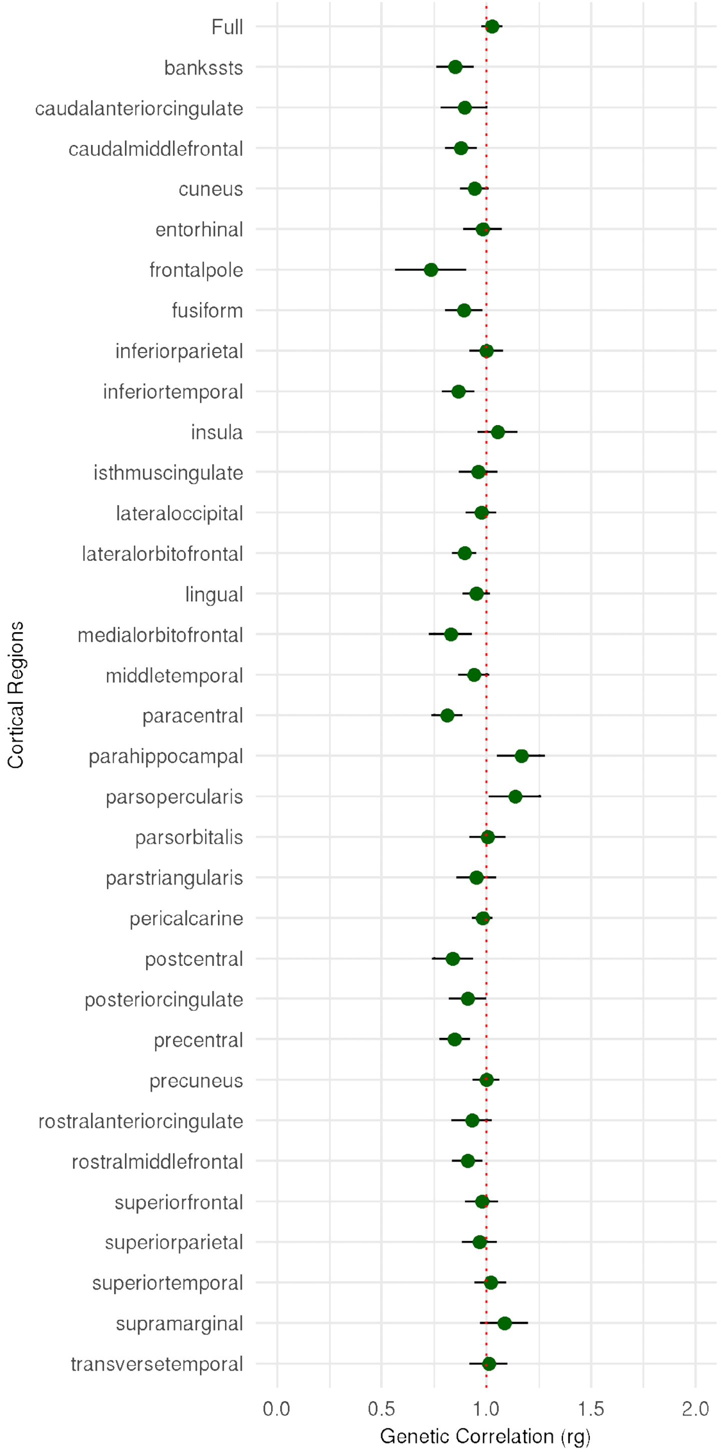

Fig. S1. Genetic correlations (LD score r_g) calculated between traits in replication dataset and respective traits in Grasby et al. (8). Traits from both datasets were all controlled for total surface area; results without genomic control were used from Grasby et al. (8). Detailed results with r_g, SE and P-values per region are provided in Table S3.

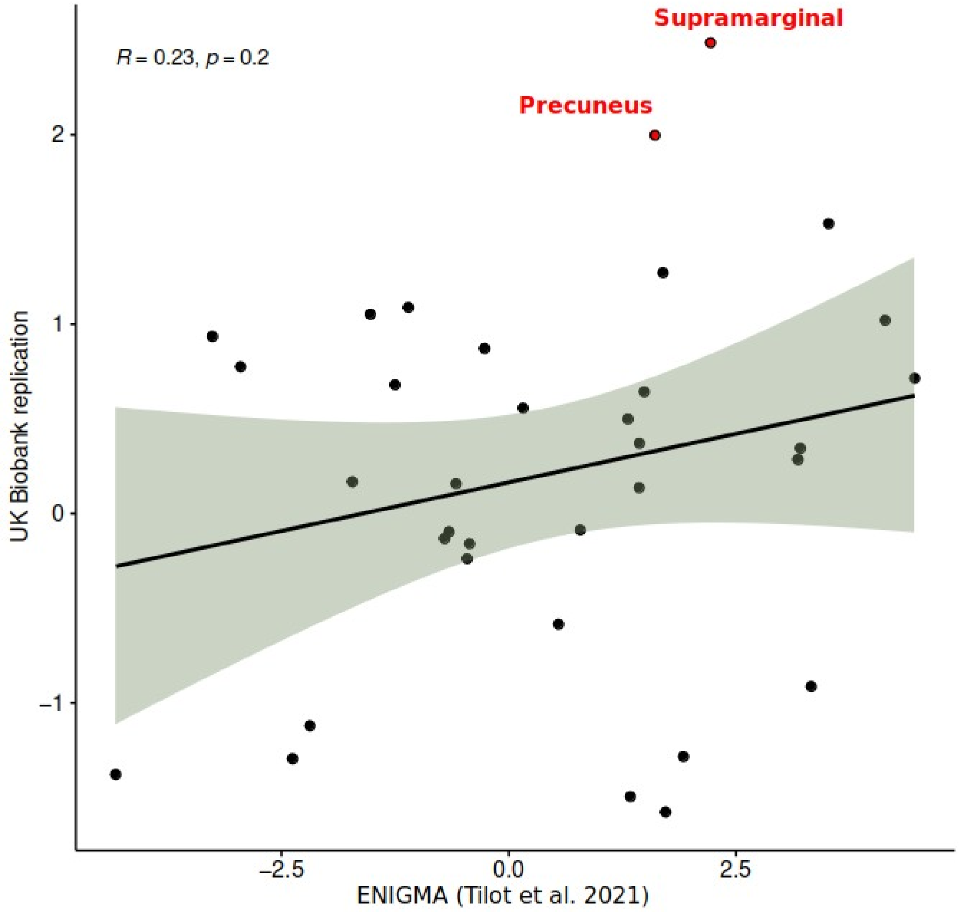

Fig. S2. Block-jacknife correlations between tSDS and ancestry regressed GWAS effect sizes of Tilot et al. (7) and the UK Biobank replication GWASs are not significantly correlated (R = 0.23, *P*-value = 0.2). Two regions that have high positive correlations are highlighted in red.

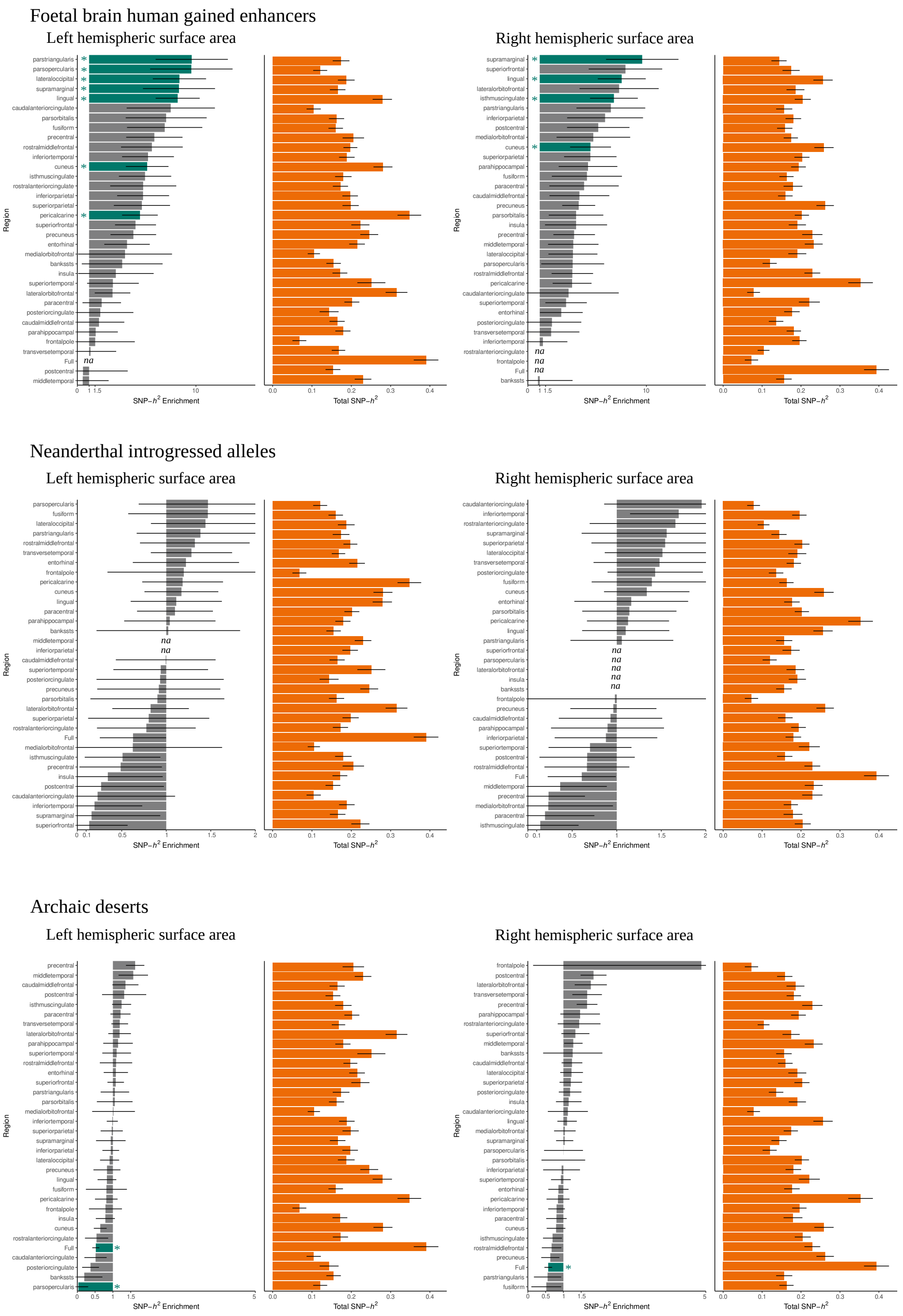

Figure S3. Log-scaled SNP-h^2 enrichment and depletion levels of full and regional left- and right-hemispheric cortical surface areas in foetal brain HGEs (top), Neanderthal introgressed allele.s (middle) and archaic deserts (bottom). Green bars and * indicate P<0.05 after FDR correction was applied for 43 independent traits (see Methods). Orange bars show SNP heritability estimates. Regions with a negative SNP-h^2 enrichment estimate are indicated as *na*. Error bars represent standard errors.

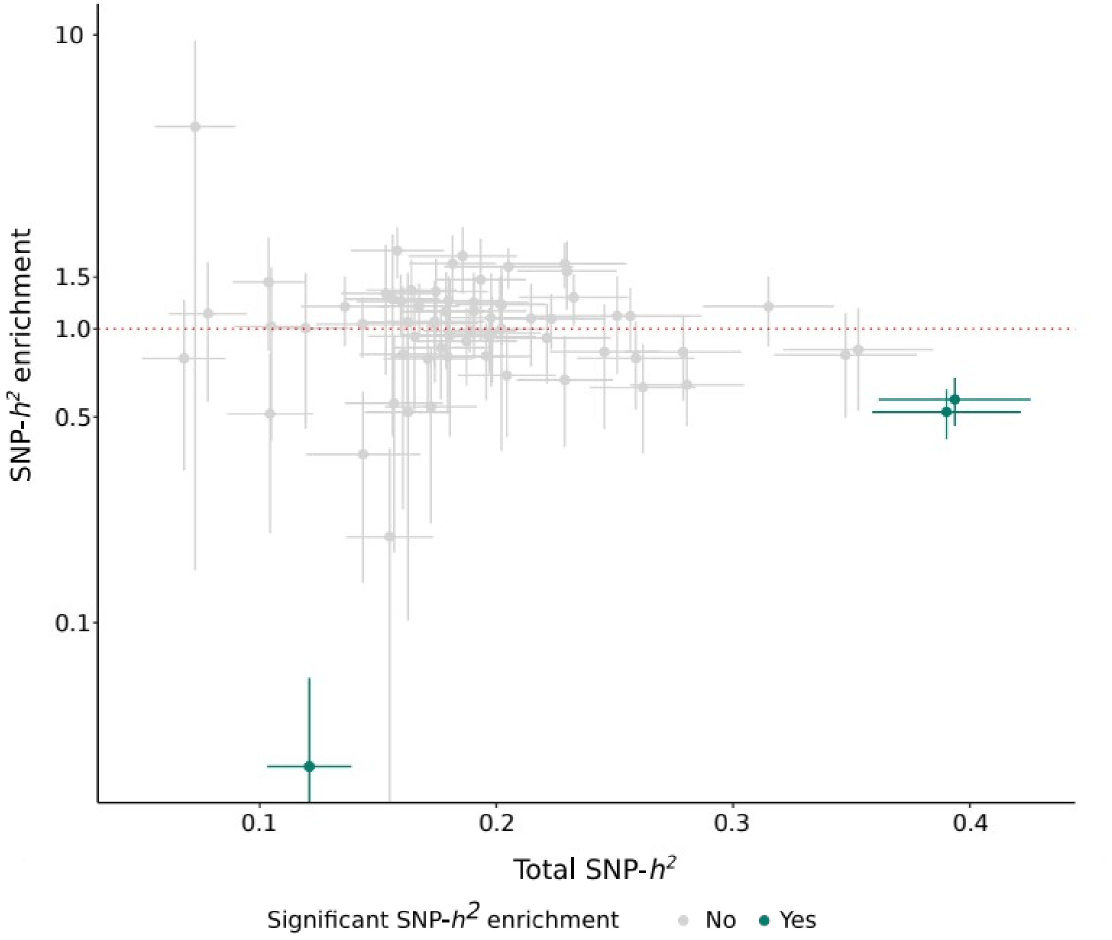

Fig. S4. The relation of total SNP-heritability to SNP-heritability enrichment/depletion in archaic deserts. Datapoints represent full and regional hemispheric surface areas. Regions that showed significant heritability depletion ($P_{FDR} < 0.05$) are indicated in green; others in grey. FDR correction was applied for 43 independent cortical regions (see Methods). Error bars represent standard errors.

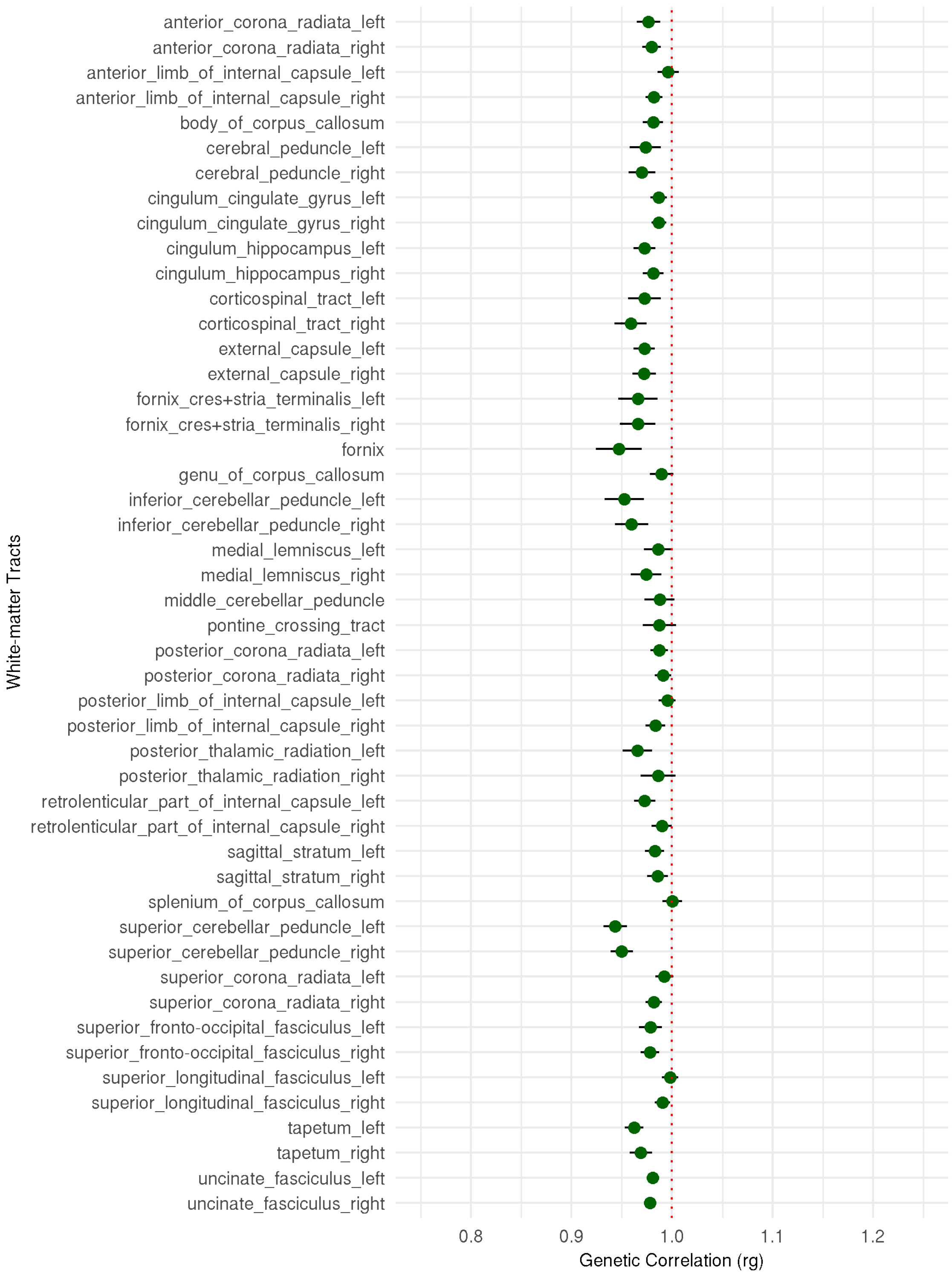

Fig S5. Genetic correlations (LD score r_g) calculated between traits in dMRI dataset and respective traits downloaded from Oxford Brain Imaging Genetics Server (10), an expanded set of genome-wide association studies of brain imaging phenotypes in UK Biobank. Detailed results with r_g, SE and P-values per region are provided in Table S11.

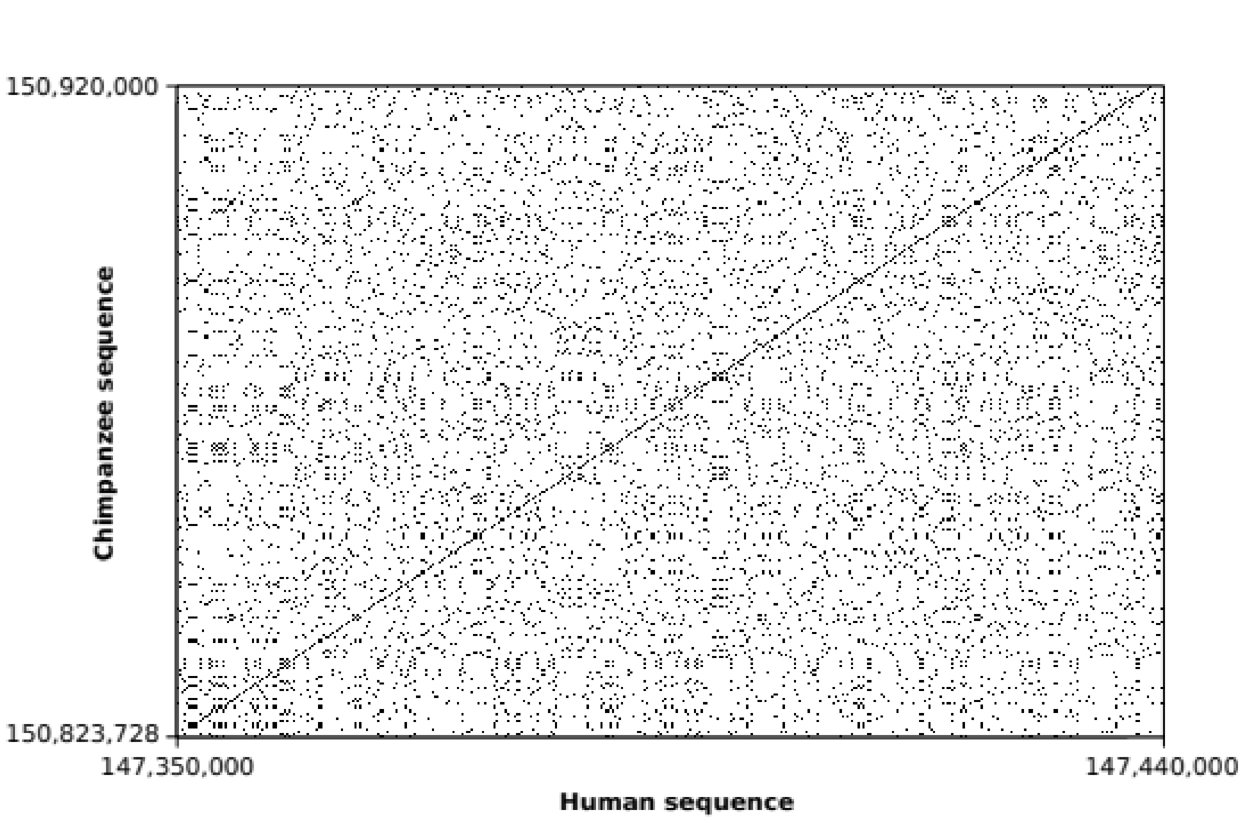

Fig. S6. Dotter plot showing sequence alignment between the 90 kilobase region surrounding *ZIC4* in human genome (hg19) and the homologous sequence in the chimpanzee genome (panTro3).

rs2853928
Left Hemisphere Total
HAR

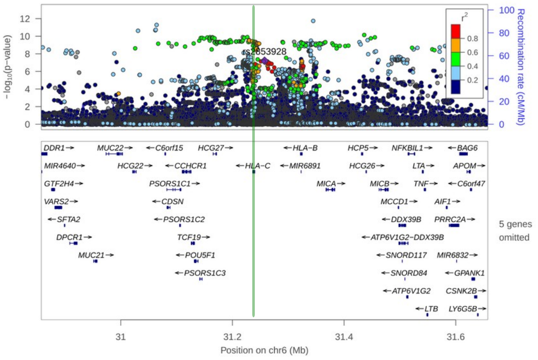

rs2853928
Right Hemisphere Total
HAR

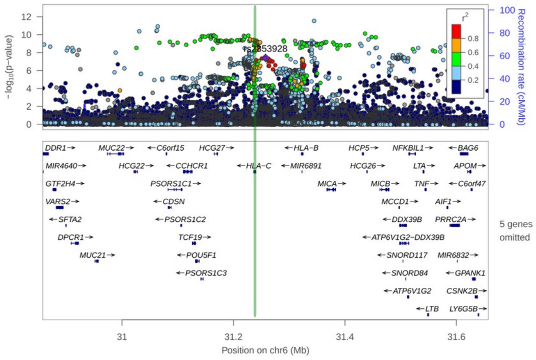

rs10230207
Left lateralorbitofrontal
HAR

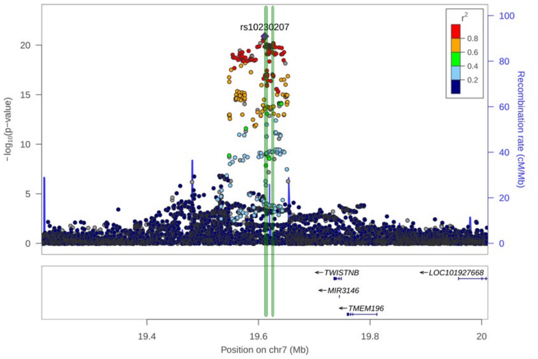

rs56207542
Left lateralorbitofrontal
HAR

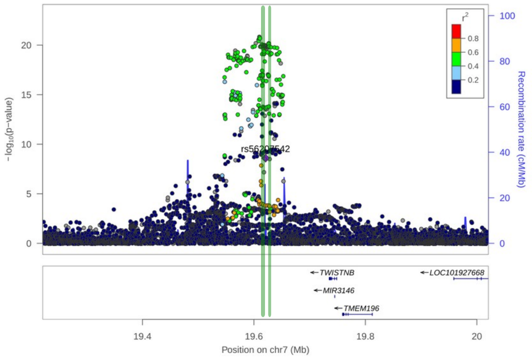

rs321403
Left insula
HAR

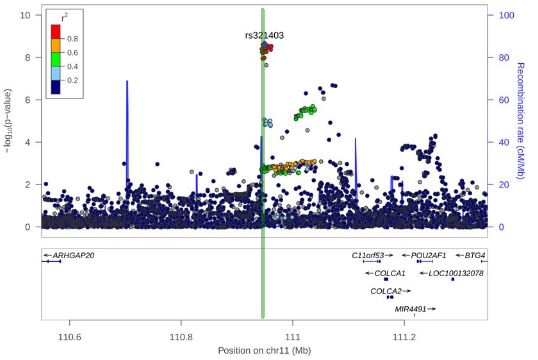

rs6965709
Right lateralorbitofrontal
HAR

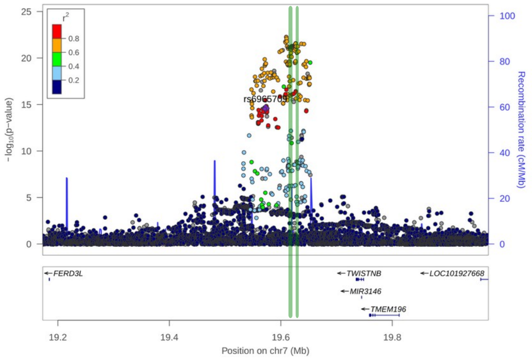

rs714392
Right lateralorbitofrontal
HAR

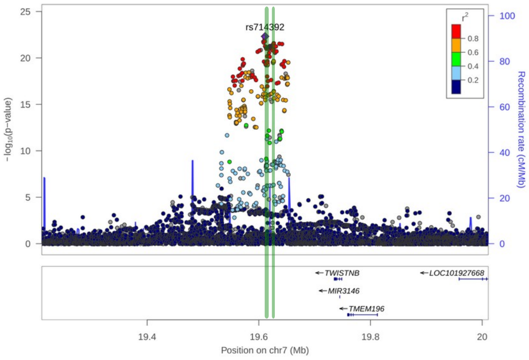

rs56207542
Right lateralorbitofrontal
HAR

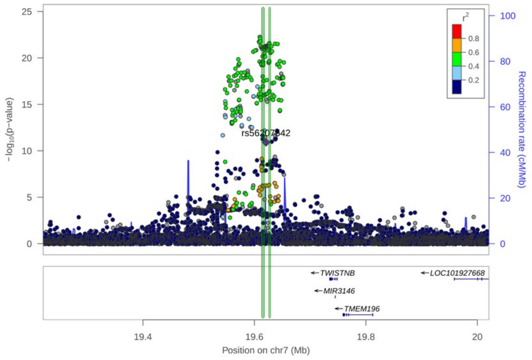

rs512182
Right middletemporal
HAR

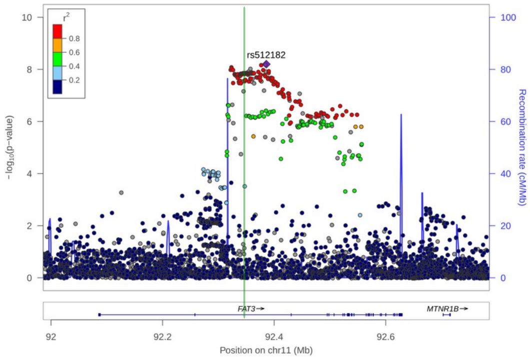

rs76715069
Right lateraloccipital
HAR

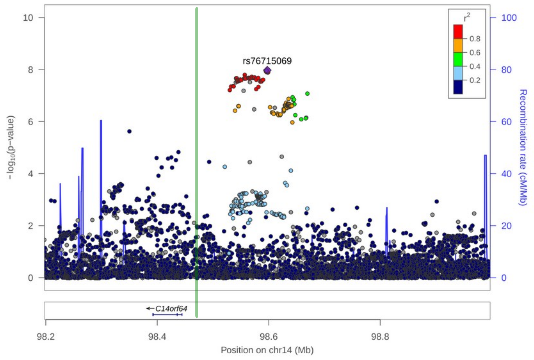

rs2857594
Left Hemisphere Total
AMH-derived DMR

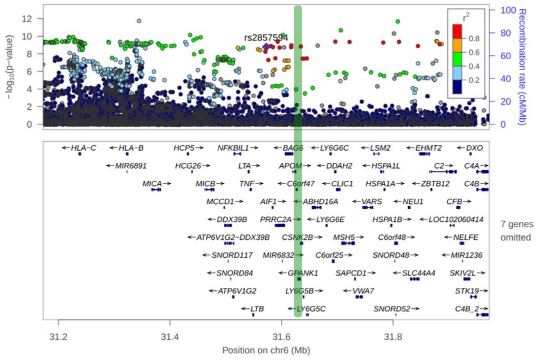

rs3130625
Right Hemisphere Total
AMH-derived DMR

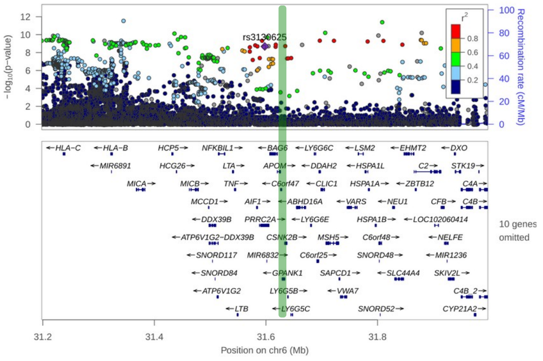

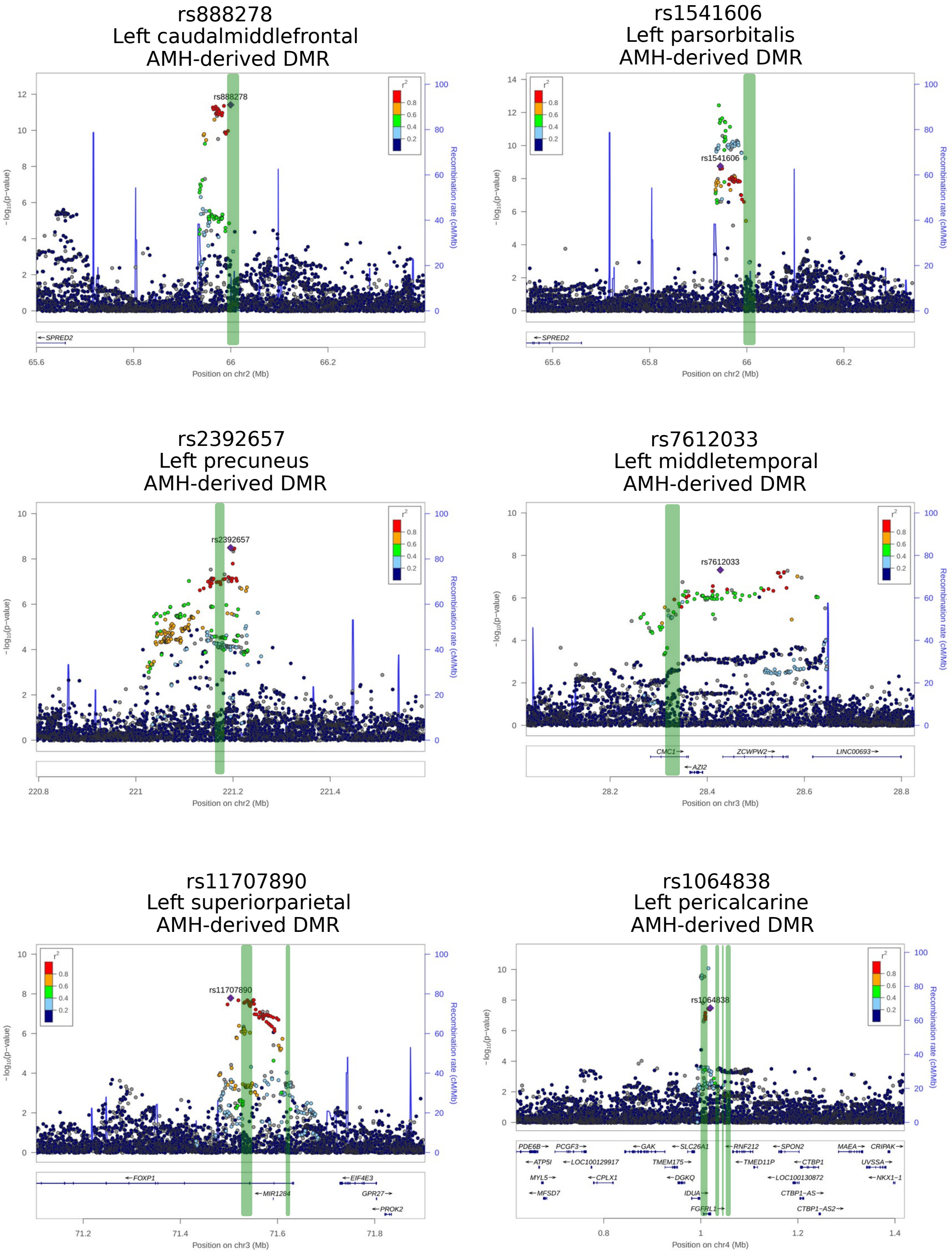
rs888278
Left caudalmiddlefrontal
AMH-derived DMR
SPRED2
Position on chr2 (Mb)
rs1541606
Left parsorbitalis
AMH-derived DMR
SPRED2
Position on chr2 (Mb)
rs2392657
Left precuneus
AMH-derived DMR
Position on chr2 (Mb)
rs7612033
Left middletemporal
AMH-derived DMR
CMC1
AZI2
ZCWPW2
LINC00693
Position on chr3 (Mb)
rs11707890
Left superiorparietal
AMH-derived DMR
FOXP1
MIR1284
EIF4E3
GPR27
PROK2
Position on chr3 (Mb)
rs1064838
Left pericalcarine
AMH-derived DMR
PDE6B
PCGF3
GAK
SLC26A1
RNF212
SPON2
MAEA
CRIPAK
ATP5I
LOC100129917
TMEM175
TMED11P
CTBP1
UVSSA
MYL5
CPLX1
DGKQ
LOC100130872
NKX1-1
MFSD7
IDUA
CTBP1-AS
FGFRL1
CTBP1-AS2
Position on chr4 (Mb)
- log10(p-value)
Recombination rate (cM/Mb)

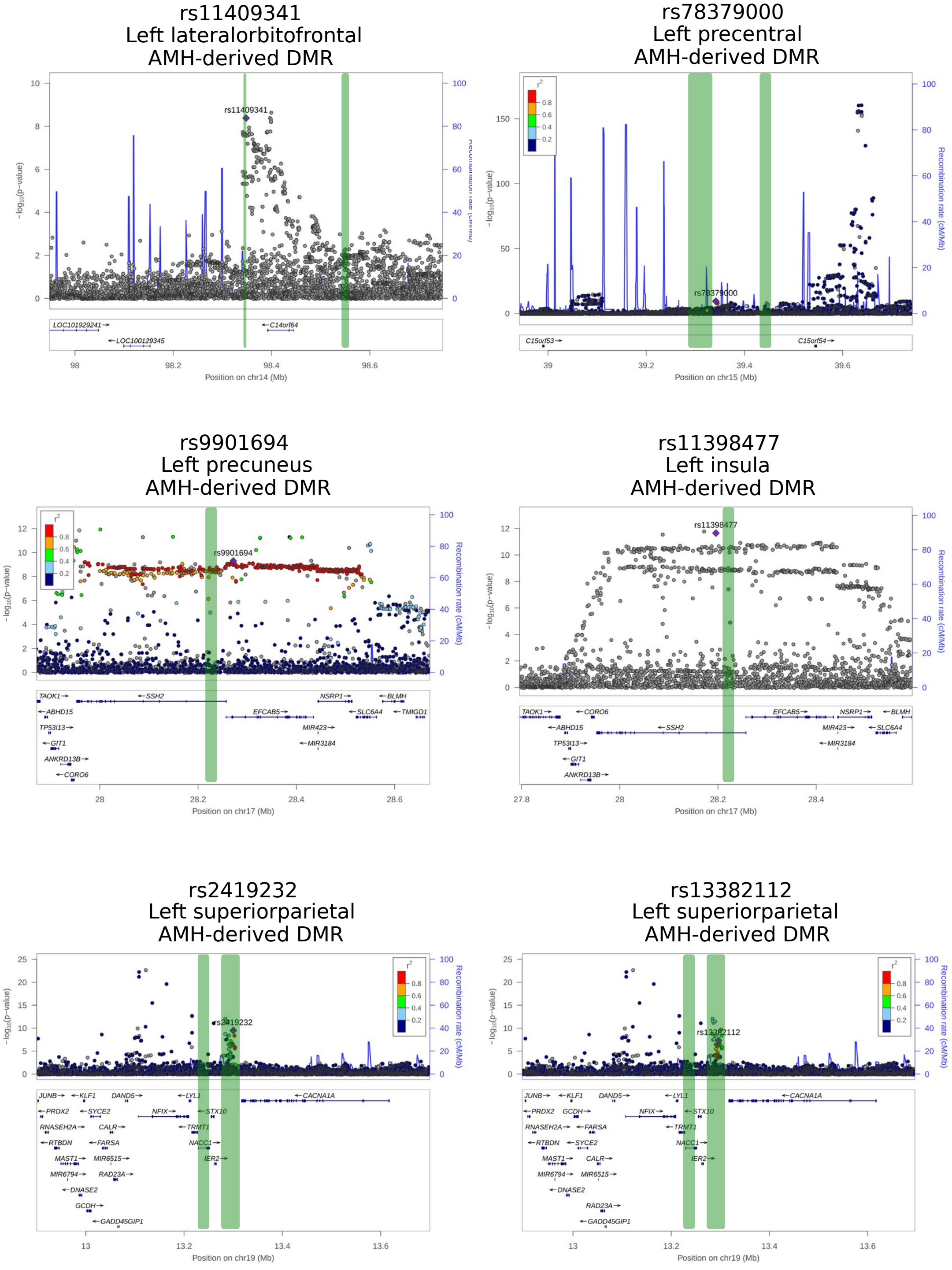
rs11409341
Left lateralorbitofrontal
AMH-derived DMR
rs78379000
Left precentral
AMH-derived DMR
rs9901694
Left precuneus
AMH-derived DMR
rs11398477
Left insula
AMH-derived DMR
rs2419232
Left superiorparietal
AMH-derived DMR
rs13382112
Left superiorparietal
AMH-derived DMR
-log10(p-value)
Recombination rate (cM/Mb)
Position on chr14 (Mb)
Position on chr15 (Mb)
Position on chr17 (Mb)
Position on chr19 (Mb)

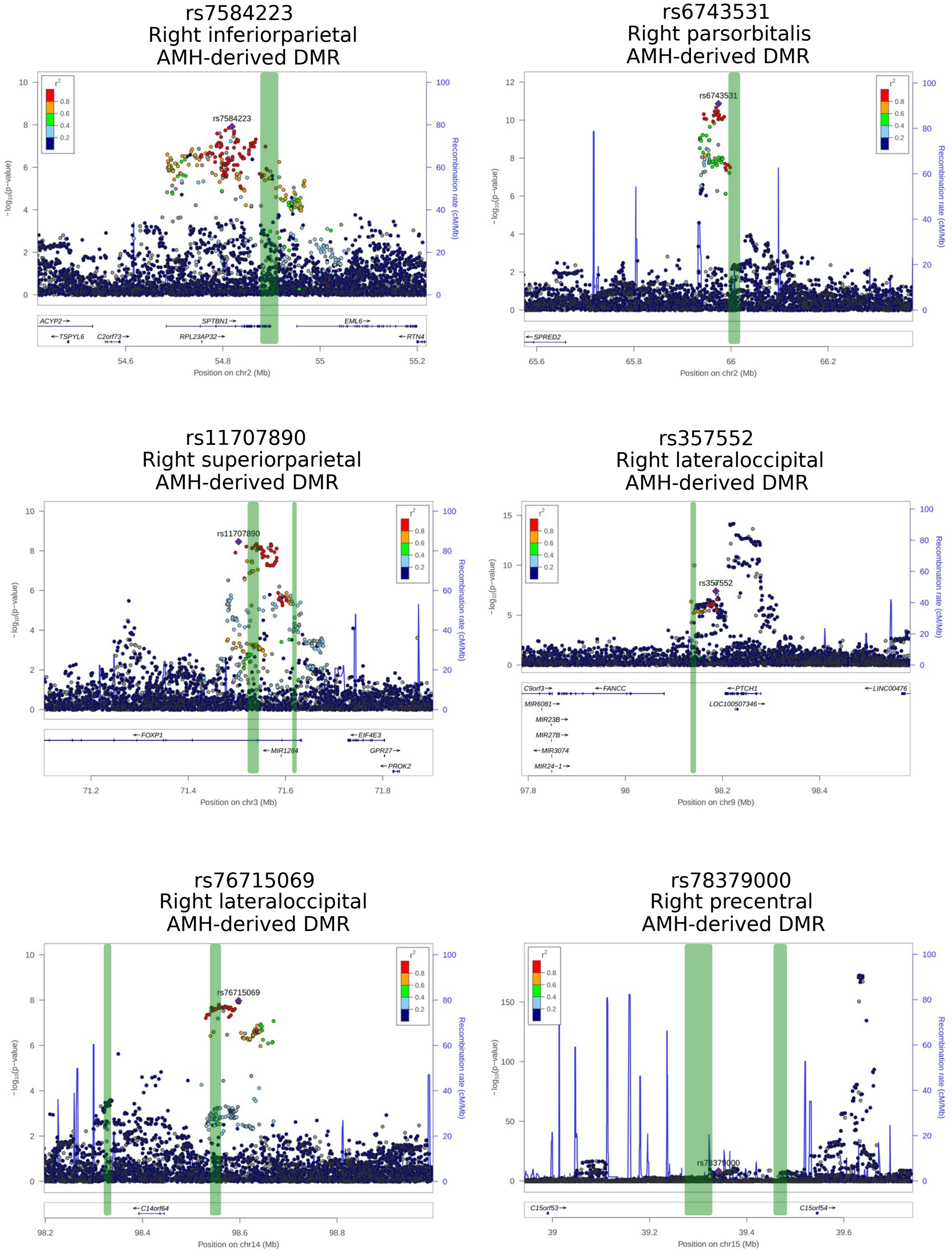

rs7584223
Right inferiorparietal
AMH-derived DMR
Position on chr2 (Mb)
rs6743531
Right parsorbitalis
AMH-derived DMR
Position on chr2 (Mb)
rs11707890
Right superiorparietal
AMH-derived DMR
Position on chr3 (Mb)
rs357552
Right lateraloccipital
AMH-derived DMR
Position on chr9 (Mb)
rs76715069
Right lateraloccipital
AMH-derived DMR
Position on chr14 (Mb)
rs78379000
Right precentral
AMH-derived DMR
Position on chr15 (Mb)
Recombination rate (cM/Mb)

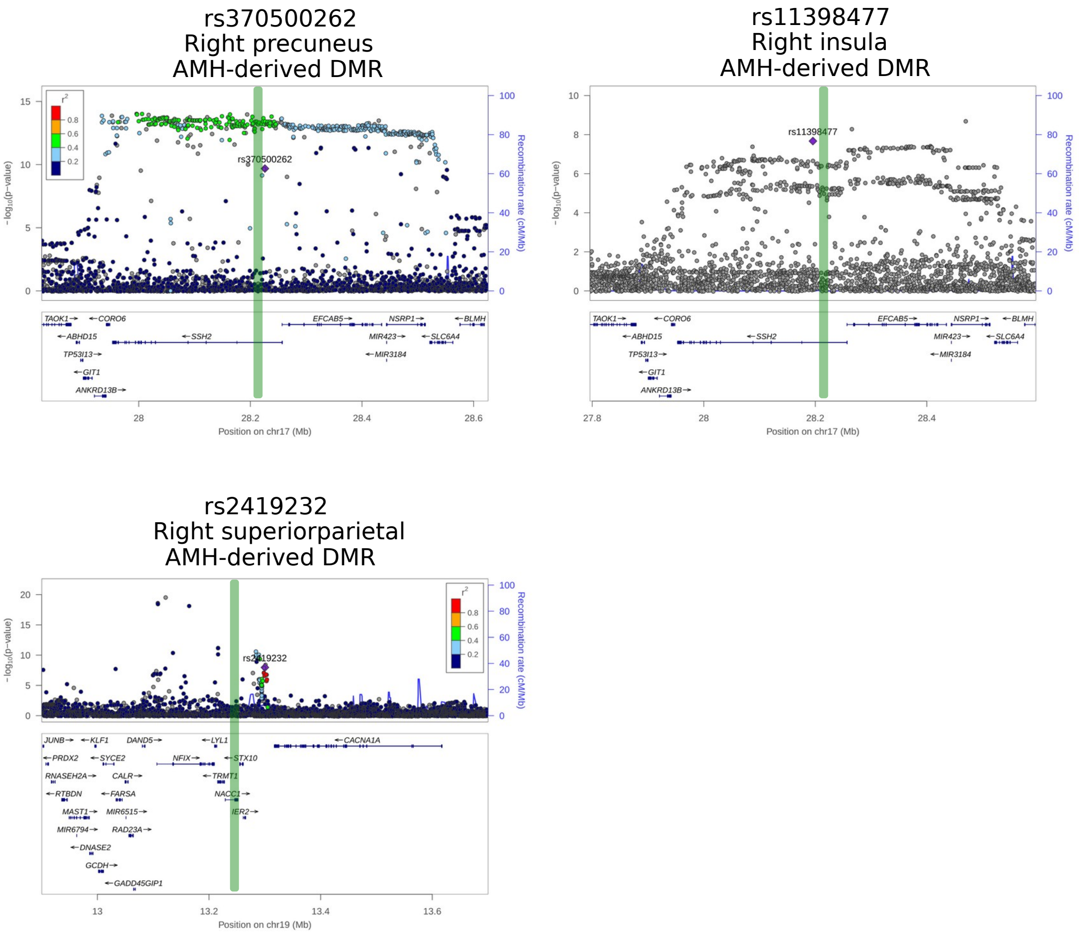

Fig. S7. LocusZoom (16) plots showing GWAS signals of the SNPs that overlap with either a HAR or an AMH-derived DMR. HAR and AMH-derived DMR elements are highlighted in green.

Supplemental Tables

Table S1. Phenotype descriptions for surface area in replication data sample. Surface area measures are the average of both hemispheres (2 IDPs per region), except for Total Surface area.

UKBB IDP		Area	N	SA Mean (mm^2)	SD
26721-2.0	26822-2.0	Total surface areas	18960	169048.88	15477.82
26722-2.0	26823-2.0	Banks of the Superior Temporal Sulcus	18939	922.97	133.57
26723-2.0	26824-2.0	Caudal Anterior Cingulate	18920	646.91	134.50
26724-2.0	26825-2.0	Caudal Middle Frontal	18953	2144.34	305.02
26725-2.0	26826-2.0	Cuneus	18956	1603.98	213.29
26726-2.0	26827-2.0	Entorhinal	18932	440.32	93.24
26727-2.0	26828-2.0	Fusiform	18954	3063.07	346.97
26728-2.0	26829-2.0	Inferior Parietal	18954	4722.65	621.81
26729-2.0	26830-2.0	Inferior Temporal	18957	3297.08	415.50
26730-2.0	26831-2.0	Isthmus Cingulate	18950	964.52	147.38
26731-2.0	26832-2.0	Lateral Occipital	18960	5037.04	592.71
26732-2.0	26833-2.0	Lateral Orbitofrontal	18957	2641.42	277.45
26733-2.0	26834-2.0	Lingual	18960	3156.08	388.07
26734-2.0	26835-2.0	Medial Orbitofrontal	18959	1963.28	206.01
26735-2.0	26836-2.0	Middle Temporal	18958	3266.67	385.87
26736-2.0	26837-2.0	Parahippocampal	18951	641.62	76.49
26737-2.0	26838-2.0	Paracentral	18955	1443.74	170.33
26738-2.0	26839-2.0	Pars Opercularis	18931	1430.40	204.66
26739-2.0	26840-2.0	Pars Orbitalis	18951	746.36	96.54
26740-2.0	26841-2.0	Pars Triangularis	18951	1378.41	190.87
26741-2.0	26842-2.0	Pericalcarine	18957	1490.99	248.06
26742-2.0	26843-2.0	Postcentral	18957	4013.70	427.98
26743-2.0	26844-2.0	Posterior Cingulate	18948	1144.83	160.95
26744-2.0	26845-2.0	Precentral	18953	4753.58	488.26
26745-2.0	26846-2.0	Precuneus	18954	3856.68	472.65
26746-2.0	26847-2.0	Rostral Anterior Cingulate	18954	718.17	131.85
26747-2.0	26848-2.0	Rostral Middle Frontal	18955	5624.17	733.51
26748-2.0	26849-2.0	Superior Frontal	18956	6972.85	822.70
26749-2.0	26850-2.0	Superior Parietal	18959	5309.44	596.81
26750-2.0	26851-2.0	Superior Temporal	18958	3750.73	394.30
26751-2.0	26852-2.0	Supramarginal	18953	3790.55	521.21
26752-2.0	26853-2.0	Frontal Pole	18949	278.84	42.05
26753-2.0	26854-2.0	Transverse Temporal	18946	390.62	65.07
26754-2.0	26855-2.0	Insula	18955	2413.24	264.71

Table S2. Univariate LDSC SNP heritability estimates for replication dataset. Regional surface area metrics were corrected for total hemisphere-averaged surface area.

Surface area (averaged across hemispheres)				
**Region**	**Total SNP-h^2**	**SE**	**Intercept**	**SE**
Total surface area	0.4022	0.0400	1.0086	0.0082
Banks of the Superior Temporal Sulcus	0.2634	0.0315	0.9990	0.0069
Caudal Anterior Cingulate	0.1438	0.0292	0.9999	0.0069
Caudal Middle Frontal	0.2518	0.0315	0.9976	0.0071
Cuneus	0.3063	0.0351	1.0192	0.0077
Entorhinal	0.2270	0.0293	1.0093	0.0069
Frontal Pole	0.1111	0.0288	0.9991	0.0073
Fusiform	0.2207	0.0294	1.0011	0.0069
Inferior Parietal	0.2457	0.0312	1.0109	0.0074
Inferior Temporal	0.2354	0.0288	1.0030	0.0072
Insula	0.2152	0.0339	1.0113	0.0079
Isthmus Cingulate	0.2433	0.0309	1.0158	0.0078
Lateral Occipital	0.2269	0.0377	1.0315	0.0085
Lateral Orbitofrontal	0.3094	0.0355	1.0006	0.0077
Lingual	0.3557	0.0395	1.0083	0.0078
Medial Orbitofrontal	0.2176	0.0306	0.9941	0.0068
Middle Temporal	0.3107	0.0330	1.0086	0.0076
Paracentral	0.2436	0.0300	1.0050	0.0071
Parahippocampal	0.2196	0.0322	1.0149	0.0069
Pars Opercularis	0.1795	0.0315	1.0072	0.0077
Pars Orbitalis	0.2277	0.0320	1.0133	0.0070
Pars Triangularis	0.2211	0.0347	1.0096	0.0072
Pericalcarine	0.3803	0.0399	1.0169	0.0077
Postcentral	0.2097	0.0315	1.0122	0.0082
Posterior Cingulate	0.1945	0.0269	1.0067	0.0072
Precentral	0.2726	0.0382	1.0142	0.0076
Precuneus	0.3150	0.0327	1.0089	0.0073
Rostral Anterior Cingulate	0.2282	0.0301	0.9983	0.0072
Rostral Middle Frontal	0.2829	0.0288	1.0076	0.0064
Superior Frontal	0.2739	0.0318	1.0125	0.0073
Superior Parietal	0.2799	0.0367	1.0120	0.0078
Superior Temporal	0.2304	0.0303	1.0163	0.0070
Supramarginal	0.1737	0.0278	1.0112	0.0070
Transverse Temporal	0.1874	0.0262	1.0135	0.0068

Table S3. Genetic correlations (LD score r_g) calculated between traits in replication dataset and respective traits in Grasby et al. (8). Traits from both datasets were all controlled for total surface area; results without genomic control were used from Grasby et al. (8).

**Trait**	**LDSC results**		
**Surface Area**	$\boldsymbol{r_g}$	**SE**	***P***
Total surface area	1.0277	0.0508	4.5182E-91
Banks of the Superior Temporal Sulcus	0.8503	0.0897	2.5112E-21
Caudal Anterior Cingulate	0.8945	0.1130	2.5043E-15
Caudal Middle Frontal	0.8786	0.0761	7.2949E-31
Cuneus	0.9437	0.0693	3.0699E-42
Entorhinal	0.9834	0.0927	2.6182E-26
Frontal Pole	0.7341	0.1707	1.6950E-05
Fusiform	0.8929	0.0900	3.4114E-23
Inferior Parietal	0.9993	0.0811	6.9260E-35
Inferior Temporal	0.8665	0.0778	8.2425E-29
Insula	1.0548	0.0955	2.3647E-28
Isthmus Cingulate	0.9615	0.0937	1.0551E-24
Lateral Occipital	0.9752	0.0729	8.1445E-41
Lateral Orbitofrontal	0.8956	0.0582	1.8297E-53
Lingual	0.9530	0.0649	9.0914E-49
Medial Orbitofrontal	0.8286	0.1023	5.6095E-16
Middle Temporal	0.9399	0.0744	1.4711E-36
Paracentral	0.8112	0.0745	1.2452E-27
Parahippocampal	1.1658	0.1152	4.6038E-24
Pars Opercularis	1.1370	0.1264	2.4231E-19
Pars Orbitalis	1.0064	0.0857	8.0056E-32
Pars Triangularis	0.9524	0.0959	3.1453E-23
Pericalcarine	0.9811	0.0497	9.9465E-87
Postcentral	0.8382	0.0990	2.5504E-17
Posterior Cingulate	0.9099	0.0896	3.0432E-24
Precentral	0.8485	0.0725	1.2539E-31
Precuneus	0.9993	0.0642	1.2190E-54
Rostral Anterior Cingulate	0.9300	0.0965	5.5869E-22
Rostral Middle Frontal	0.9095	0.0738	7.0542E-35
Superior Frontal	0.9786	0.0786	1.4443E-35
Superior Parietal	0.9667	0.0835	5.4737E-31
Superior Temporal	1.0201	0.0757	2.2197E-41
Supramarginal	1.0861	0.1146	2.5610E-21
Transverse Temporal	1.0105	0.0917	3.0755E-28

Table S4. Singleton Density Score-GWAS effect size correlations and *P*-values for the replication study.

Brain Region	Correlation Coefficient	Z-score	*P*
Total surface area	0.006	1.531	0.126
Caudal middle frontal	0.002	0.345	0.731
Insula	0.001	0.285	0.776
Isthmuscingulate	0.004	0.935	0.349
Lateral occipital	-0.003	-0.913	0.361
Pars opercularis	0.004	1.02	0.308
Postcentral	0.003	0.775	0.438
Precentral	-0.006	-1.377	0.168
Rostralanteriorcingulate	0.003	0.715	0.475

Table S5. Phenotype descriptions for surface area in hemisphere-specific data sample.

UKBB IDP	Area	Hemisphere	N	SA Mean (mm^2)	SD
26721-2.0	Total surface area	left	30332	84334.91	7713.29
26722-2.0	Banks of the Superior Temporal Sulcus	left	30301	974.01	154.99
26723-2.0	Caudal Anterior Cingulate	left	30269	608.70	132.29
26724-2.0	Caudal Middle Frontal	left	30321	2195.63	330.39
26725-2.0	Cuneus	left	30327	1556.60	223.25
26726-2.0	Entorhinal	left	30277	470.65	91.76
26727-2.0	Fusiform	left	30317	3103.28	360.73
26728-2.0	Inferior Parietal	left	30320	4316.74	608.24
26729-2.0	Inferior Temporal	left	30324	3345.84	452.37
26730-2.0	Isthmus Cingulate	left	30314	1021.64	163.65
26731-2.0	Lateral Occipital	left	30332	5028.46	617.91
26732-2.0	Lateral Orbitofrontal	left	30328	2646.84	272.42
26733-2.0	Lingual	left	30332	3066.78	397.62
26734-2.0	Medial Orbitofrontal	left	30329	1931.37	233.79
26735-2.0	Middle Temporal	left	30326	3108.36	403.02
26736-2.0	Parahippocampal	left	30322	656.94	76.92
26737-2.0	Paracentral	left	30320	1364.87	165.93
26738-2.0	Pars Opercularis	left	30285	1543.46	226.47
26739-2.0	Pars Orbitalis	left	30319	678.78	87.87
26740-2.0	Pars Triangularis	left	30322	1277.16	187.30
26741-2.0	Pericalcarine	left	30329	1415.52	248.55
26742-2.0	Postcentral	left	30323	4069.73	451.60
26743-2.0	Posterior Cingulate	left	30313	1143.90	170.40
26744-2.0	Precentral	left	30322	4752.74	495.89
26745-2.0	Precuneus	left	30322	3783.39	474.15
26746-2.0	Rostral Anterior Cingulate	left	30321	845.05	171.09
26747-2.0	Rostral Middle Frontal	left	30321	5517.75	738.82
26748-2.0	Superior Frontal	left	30321	7115.94	857.58
26749-2.0	Superior Parietal	left	30327	5335.05	643.93
26750-2.0	Superior Temporal	left	30327	3883.73	446.77
26751-2.0	Supramarginal	left	30320	3975.02	620.78
26752-2.0	Frontal Pole	left	30321	250.54	29.75
26753-2.0	Transverse Temporal	left	30302	450.51	70.94
26754-2.0	Insula	left	30326	2429.11	259.97
26822-2.0	Total surface area	right	30332	85085.85	7851.80
26823-2.0	Banks of the Superior Temporal Sulcus	right	30301	883.07	119.62
26824-2.0	Caudal Anterior Cingulate	right	30269	693.65	147.93
26825-2.0	Caudal Middle Frontal	right	30321	2100.60	330.74
26826-2.0	Cuneus	right	30327	1660.87	227.91
26827-2.0	Entorhinal	right	30277	415.30	77.08

26828-2.0	Fusiform	right	30317	3051.38	372.08
26829-2.0	Inferior Parietal	right	30320	5160.66	731.21
26830-2.0	Inferior Temporal	right	30324	3277.01	439.33
26831-2.0	Isthmus Cingulate	right	30314	910.73	143.02
26832-2.0	Lateral Occipital	right	30332	5075.18	656.26
26833-2.0	Lateral Orbitofrontal	right	30328	2655.35	308.33
26834-2.0	Lingual	right	30332	3273.42	434.02
26835-2.0	Medial Orbitofrontal	right	30329	2003.04	217.82
26836-2.0	Middle Temporal	right	30326	3447.00	406.66
26837-2.0	Parahippocampal	right	30322	631.69	75.01
26838-2.0	Paracentral	right	30320	1522.14	196.35
26839-2.0	Pars Opercularis	right	30285	1329.20	187.33
26840-2.0	Pars Orbitalis	right	30319	820.77	106.69
26841-2.0	Pars Triangularis	right	30322	1486.19	225.66
26842-2.0	Pericalcarine	right	30329	1574.92	269.64
26843-2.0	Postcentral	right	30323	3966.20	454.39
26844-2.0	Posterior Cingulate	right	30313	1152.61	175.38
26845-2.0	Precentral	right	30322	4756.43	507.21
26846-2.0	Precuneus	right	30322	3952.12	498.11
26847-2.0	Rostral Anterior Cingulate	right	30321	599.60	125.05
26848-2.0	Rostral Middle Frontal	right	30321	5749.92	786.65
26849-2.0	Superior Frontal	right	30321	6855.09	847.21
26850-2.0	Superior Parietal	right	30327	5305.62	623.30
26851-2.0	Superior Temporal	right	30327	3635.34	387.38
26852-2.0	Supramarginal	right	30320	3627.34	510.53
26853-2.0	Frontal Pole	right	30321	309.67	37.16
26854-2.0	Transverse Temporal	right	30302	334.27	47.66
26855-2.0	Insula	right	30326	2404.50	298.19

Table S6. Univariate LDSC SNP-heritability overview for the hemisphere-specific analysis. Hemispheric regional surface area measures were corrected for the corresponding total hemispheric surface area.

**Surface area (per hemisphere)**					
**Region**	**Hemisphere**	**Total SNP-*h*²**	**SE**	**Intercept**	**SE**
Total surface area	left	0.3902	0.0314	1.0161	0.0086
Banks of the Superior Temporal Sulcus	left	0.1549	0.0184	1.0055	0.0060
Caudal Anterior Cingulate	left	0.1043	0.0181	1.0073	0.0069
Caudal Middle Frontal	left	0.1640	0.0192	1.0127	0.0071
Cuneus	left	0.2805	0.0242	1.0158	0.0073
Entorhinal	left	0.2146	0.0198	1.0088	0.0070
Frontal Pole	left	0.0680	0.0176	1.0064	0.0065
Fusiform	left	0.1605	0.0184	1.0019	0.0063
Inferior Parietal	left	0.1970	0.0198	1.0154	0.0077
Inferior Temporal	left	0.1887	0.0197	0.9937	0.0070
Insula	left	0.1710	0.0189	1.0157	0.0064
Isthmus Cingulate	left	0.1798	0.0210	1.0227	0.0070
Lateral Occipital	left	0.1873	0.0213	1.0285	0.0076
Lateral Orbitofrontal	left	0.3150	0.0277	0.9967	0.0080
Lingual	left	0.2789	0.0246	1.0169	0.0083
Medial Orbitofrontal	left	0.1048	0.0155	0.9988	0.0060
Middle Temporal	left	0.2298	0.0211	1.0153	0.0072
Paracentral	left	0.2015	0.0190	0.9979	0.0071
Parahippocampal	left	0.1787	0.0193	1.0174	0.0072
Pars Opercularis	left	0.1209	0.0178	1.0089	0.0070
Pars Orbitalis	left	0.1625	0.0194	1.0103	0.0068
Pars Triangularis	left	0.1739	0.0213	1.0173	0.0074
Pericalcarine	left	0.3475	0.0301	1.0222	0.0086
Postcentral	left	0.1533	0.0189	1.0124	0.0069
Posterior Cingulate	left	0.1436	0.0241	1.0021	0.0078
Precentral	left	0.2051	0.0272	1.0239	0.0076
Precuneus	left	0.2456	0.0229	1.0149	0.0066
Rostral Anterior Cingulate	left	0.1723	0.0193	1.0059	0.0066
Rostral Middle Frontal	left	0.1977	0.0178	1.0095	0.0069
Superior Frontal	left	0.2232	0.0230	1.0155	0.0078
Superior Parietal	left	0.1982	0.0206	1.0212	0.0079
Superior Temporal	left	0.2510	0.0357	1.0045	0.0100
Supramarginal	left	0.1654	0.0198	1.0037	0.0068
Transverse Temporal	left	0.1673	0.0171	1.0113	0.0063
Total surface area	right	0.3937	0.0321	1.0155	0.0086
Banks of the Superior Temporal Sulcus	right	0.1561	0.0200	1.0038	0.0070
Caudal Anterior Cingulate	right	0.0781	0.0166	1.0125	0.0063
Caudal Middle Frontal	right	0.1596	0.0187	0.9962	0.0066
Cuneus	right	0.2589	0.0249	1.0206	0.0072
Entorhinal	right	0.1766	0.0199	1.0157	0.0069
Frontal Pole	right	0.0727	0.0170	0.9941	0.0067
Fusiform	right	0.1626	0.0183	1.0173	0.0066
Inferior Parietal	right	0.1803	0.0200	1.0207	0.0073
Inferior Temporal	right	0.1957	0.0185	1.0051	0.0078
Insula	right	0.1905	0.0222	1.0046	0.0081

Isthmus Cingulate	right	0.2044	0.0206	1.0147	0.0070
Lateral Occipital	right	0.1904	0.0231	1.0333	0.0075
Lateral Orbitofrontal	right	0.1857	0.0230	1.0051	0.0074
Lingual	right	0.2566	0.0248	1.0289	0.0078
Medial Orbitofrontal	right	0.1738	0.0183	0.9959	0.0074
Middle Temporal	right	0.2326	0.0231	1.0120	0.0076
Paracentral	right	0.1794	0.0242	1.0110	0.0078
Parahippocampal	right	0.1934	0.0190	1.0073	0.0065
Pars Opercularis	right	0.1194	0.0181	1.0104	0.0065
Pars Orbitalis	right	0.2021	0.0184	1.0096	0.0068
Pars Triangularis	right	0.1567	0.0207	1.0030	0.0081
Pericalcarine	right	0.3529	0.0316	1.0254	0.0082
Postcentral	right	0.1581	0.0197	1.0147	0.0071
Posterior Cingulate	right	0.1360	0.0186	1.0043	0.0072
Precentral	right	0.2289	0.0260	0.9986	0.0069
Precuneus	right	0.2619	0.0223	1.0066	0.0072
Rostral Anterior Cingulate	right	0.1037	0.0152	1.0001	0.0058
Rostral Middle Frontal	right	0.2289	0.0203	1.0059	0.0068
Superior Frontal	right	0.1744	0.0220	1.0256	0.0068
Superior Parietal	right	0.2023	0.0194	1.0051	0.0064
Superior Temporal	right	0.2213	0.0270	1.0174	0.0084
Supramarginal	right	0.1434	0.0196	1.0058	0.0066
Transverse Temporal	right	0.1815	0.0185	1.0128	0.0064

Table S7 LDSC partitioned heritability analysis results for left-hemispheric surface area metrics. FDR column shows FDR corrected (n=43) *P*-values. fetal_hge, *fetal brain human gained enhancers*; archaic_deserts, *Archaic deserts;* nean_introgressed, *Neanderthal introgressed alleles.* * Some FDR values are smaller than Enrichment *P* as FDR correction was applied for the total number of independent traits. Significant enrichments are marked in bold.

Region	Annotation	Prop. of SNPs	Prop. of h^2	Prop. of h^2 SE	Enrichment	Enrichment SE	Enrichment *P*	FDR
Total surface area	fetal hge	0.0145	-0.0171	0.0219	-1.1832	1.5153	0.1532	0.1734
Banks of the Superior Temporal Sulcus	fetal hge	0.0145	0.0547	0.0493	3.7834	3.4070	0.4143	0.3426
Caudal Anterior Cingulate	fetal hge	0.0145	0.1139	0.0538	7.8784	3.7179	0.0539	0.0868
Caudal Middle Frontal	fetal hge	0.0145	0.0265	0.0309	1.8338	2.1351	0.6991	0.5010
**Cuneus**	**fetal hge**	**0.0145**	**0.0852**	**0.0260**	**5.8943**	**1.7981**	**0.0061**	**0.0291**
Entorhinal	fetal hge	0.0145	0.0606	0.0277	4.1902	1.9170	0.0889	0.1196
Frontal Pole	fetal hge	0.0145	0.0219	0.0482	1.5115	3.3338	0.8790	0.5820
Fusiform	fetal hge	0.0145	0.1066	0.0459	7.3691	3.1725	0.0492	0.0868
Inferior Parietal	fetal hge	0.0145	0.0802	0.0317	5.5439	2.1894	0.0440	0.0822
Inferior Temporal	fetal hge	0.0145	0.0862	0.0316	5.9604	2.1859	0.0235	0.0595
Insula	fetal hge	0.0145	0.0471	0.0460	3.2541	3.1803	0.4769	0.3798
Isthmus Cingulate	fetal hge	0.0145	0.0826	0.0319	5.7123	2.2070	0.0326	0.0700
**Lateral Occipital**	**fetal hge**	**0.0145**	**0.1245**	**0.0325**	**8.6126**	**2.2443**	**0.0013**	**0.0115**
Lateral Orbitofrontal	fetal hge	0.0145	0.0430	0.0217	2.9707	1.4998	0.1912	0.1805
**Lingual**	**fetal hge**	**0.0145**	**0.1223**	**0.0268**	**8.4598**	**1.8547**	**0.0001**	**0.0029**
Medial Orbitofrontal	fetal hge	0.0145	0.0582	0.0573	4.0225	3.9596	0.4566	0.3705
Middle Temporal	fetal hge	0.0145	0.0067	0.0316	0.4648	2.1821	0.8049	0.5494
Paracentral	fetal hge	0.0145	0.0298	0.0235	2.0605	1.6236	0.5133	0.4013
Parahippocampal	fetal hge	0.0145	0.0225	0.0270	1.5541	1.8683	0.7669	0.5319
**Pars Opercularis**	**fetal hge**	**0.0145**	**0.1390**	**0.0502**	**9.6100**	**3.4750**	**0.0124**	**0.0444**
Pars Orbitalis	fetal hge	0.0145	0.1084	0.0493	7.4933	3.4118	0.0415	0.0810
**Pars Triangularis**	**fetal hge**	**0.0145**	**0.1395**	**0.0440**	**9.6479**	**3.0440**	**0.0024**	**0.0147**
**Pericalcarine**	**fetal hge**	**0.0145**	**0.0764**	**0.0217**	**5.2814**	**1.5036**	**0.0053**	**0.0287**
Postcentral	fetal hge	0.0145	0.0069	0.0546	0.4755	3.7767	0.8883	0.5820
Posterior Cingulate	fetal hge	0.0145	0.0281	0.0404	1.9411	2.7909	0.7349	0.5180
Precentral	fetal hge	0.0145	0.0940	0.0343	6.4998	2.3710	0.0169	0.0519
Precuneus	fetal hge	0.0145	0.0683	0.0278	4.7268	1.9195	0.0565	0.0868
Rostral Anterior Cingulate	fetal hge	0.0145	0.0803	0.0402	5.5567	2.7812	0.0891	0.1196
Rostral Middle Frontal	fetal hge	0.0145	0.0909	0.0375	6.2878	2.5961	0.0350	0.0718
Superior Frontal	fetal hge	0.0145	0.0710	0.0250	4.9067	1.7308	0.0233	0.0595
Superior Parietal	fetal hge	0.0145	0.0788	0.0343	5.4466	2.3691	0.0564	0.0868

Superior Temporal	fetal hge	0.0145	0.0435	0.0315	3.0062	2.1760	0.3415	0.2879
**Supramarginal**	**fetal hge**	**0.0145**	**0.1239**	**0.0439**	**8.5718**	**3.0348**	**0.0124**	**0.0444**
Transverse Temporal	fetal hge	0.0145	0.0161	0.0313	1.1168	2.1612	0.9569	0.6069
**Total surface area**	**archaic deserts**	**0.0270**	**0.0141**	**0.0027**	**0.5213**	**0.0996**	**0.0000**	**0.0006**
Banks of the Superior Temporal Sulcus	archaic deserts	0.0270	0.0053	0.0138	0.1961	0.5103	0.1054	0.3777
Caudal Anterior Cingulate	archaic deserts	0.0270	0.0139	0.0084	0.5137	0.3121	0.1279	0.4230
Caudal Middle Frontal	archaic deserts	0.0270	0.0366	0.0099	1.3546	0.3662	0.3227	0.6044
Cuneus	archaic deserts	0.0270	0.0174	0.0048	0.6455	0.1793	0.0511	0.2442
Entorhinal	archaic deserts	0.0270	0.0293	0.0092	1.0841	0.3403	0.8040	0.6117
Frontal Pole	archaic deserts	0.0270	0.0214	0.0125	0.7933	0.4639	0.6595	0.6117
Fusiform	archaic deserts	0.0270	0.0221	0.0156	0.8186	0.5756	0.7550	0.6117
Inferior Parietal	archaic deserts	0.0270	0.0255	0.0063	0.9415	0.2346	0.8048	0.6117
Inferior Temporal	archaic deserts	0.0270	0.0266	0.0042	0.9851	0.1562	0.9246	0.6117
Insula	archaic deserts	0.0270	0.0213	0.0072	0.7897	0.2680	0.4439	0.6044
Isthmus Cingulate	archaic deserts	0.0270	0.0337	0.0073	1.2466	0.2690	0.3536	0.6044
Lateral Occipital	archaic deserts	0.0270	0.0246	0.0072	0.9086	0.2676	0.7348	0.6117
Lateral Orbitofrontal	archaic deserts	0.0270	0.0322	0.0086	1.1901	0.3186	0.5469	0.6117
Lingual	archaic deserts	0.0270	0.0225	0.0071	0.8337	0.2630	0.5301	0.6117
Medial Orbitofrontal	archaic deserts	0.0270	0.0275	0.0163	1.0188	0.6014	0.9748	0.6316
Middle Temporal	archaic deserts	0.0270	0.0425	0.0112	1.5731	0.4130	0.1541	0.4418
Paracentral	archaic deserts	0.0270	0.0328	0.0077	1.2147	0.2835	0.4498	0.6044
Parahippocampal	archaic deserts	0.0270	0.0310	0.0112	1.1453	0.4155	0.7271	0.6117
**Pars Opercularis**	**archaic deserts**	**0.0270**	**0.0009**	**0.0074**	**0.0324**	**0.2724**	**0.0005**	**0.0079**
Pars Orbitalis	archaic deserts	0.0270	0.0285	0.0135	1.0526	0.5000	0.9144	0.6117
Pars Triangularis	archaic deserts	0.0270	0.0286	0.0108	1.0581	0.4010	0.8841	0.6117
Pericalcarine	archaic deserts	0.0270	0.0220	0.0086	0.8147	0.3173	0.5588	0.6117
Postcentral	archaic deserts	0.0270	0.0356	0.0168	1.3174	0.6216	0.6061	0.6117
Posterior Cingulate	archaic deserts	0.0270	0.0101	0.0064	0.3740	0.2374	0.0221	0.1357
Precentral	archaic deserts	0.0270	0.0440	0.0070	1.6259	0.2586	0.0058	0.0622
Precuneus	archaic deserts	0.0270	0.0226	0.0102	0.8345	0.3778	0.6628	0.6117
Rostral Anterior Cingulate	archaic deserts	0.0270	0.0147	0.0088	0.5425	0.3247	0.1668	0.4481
Rostral Middle Frontal	archaic deserts	0.0270	0.0295	0.0123	1.0895	0.4544	0.8432	0.6117
Superior Frontal	archaic deserts	0.0270	0.0292	0.0063	1.0807	0.2315	0.7281	0.6117
Superior Parietal	archaic deserts	0.0270	0.0262	0.0084	0.9678	0.3100	0.9172	0.6117
Superior Temporal	archaic deserts	0.0270	0.0299	0.0109	1.1060	0.4040	0.7776	0.6117
Supramarginal	archaic deserts	0.0270	0.0255	0.0112	0.9426	0.4160	0.8905	0.6117
Transverse Temporal	archaic deserts	0.0270	0.0322	0.0064	1.1924	0.2353	0.4052	0.6044
Total surface area	nean introgressed	0.0113	0.0071	0.0042	0.6266	0.3727	0.3144	0.6146

Banks of the Superior Temporal Sulcus	nean introgressed	0.0113	0.0115	0.0091	1.0233	0.8068	0.9769	0.6294
Caudal Anterior Cingulate	nean introgressed	0.0113	0.0026	0.0098	0.2287	0.8711	0.3947	0.6198
Caudal Middle Frontal	nean introgressed	0.0113	0.0112	0.0063	0.9939	0.5604	0.9913	0.6294
Cuneus	nean introgressed	0.0113	0.0132	0.0047	1.1708	0.4147	0.6796	0.6198
Entorhinal	nean introgressed	0.0113	0.0138	0.0067	1.2214	0.5970	0.7093	0.6198
Frontal Pole	nean introgressed	0.0113	0.0135	0.0097	1.1972	0.8574	0.8153	0.6198
Fusiform	nean introgressed	0.0113	0.0166	0.0101	1.4671	0.8941	0.5898	0.6198
Inferior Parietal	nean introgressed	0.0113	-0.0015	0.0059	-0.1326	0.5191	0.0282	0.2873
Inferior Temporal	nean introgressed	0.0113	0.0022	0.0060	0.1950	0.5349	0.1295	0.5061
Insula	nean introgressed	0.0113	0.0039	0.0069	0.3447	0.6157	0.2991	0.6146
Isthmus Cingulate	nean introgressed	0.0113	0.0058	0.0048	0.5097	0.4250	0.2516	0.6146
Lateral Occipital	nean introgressed	0.0113	0.0163	0.0069	1.4406	0.6121	0.4656	0.6198
Lateral Orbitofrontal	nean introgressed	0.0113	0.0093	0.0049	0.8246	0.4299	0.6826	0.6198
Lingual	nean introgressed	0.0113	0.0126	0.0058	1.1128	0.5125	0.8243	0.6198
Medial Orbitofrontal	nean introgressed	0.0113	0.0071	0.0113	0.6252	1.0027	0.7048	0.6198
Middle Temporal	nean introgressed	0.0113	-0.0058	0.0055	-0.5150	0.4845	0.0010	0.0421
Paracentral	nean introgressed	0.0113	0.0124	0.0048	1.0985	0.4263	0.8169	0.6198
Parahippocampal	nean introgressed	0.0113	0.0117	0.0058	1.0405	0.5127	0.9370	0.6198
Pars Opercularis	nean introgressed	0.0113	0.0166	0.0088	1.4686	0.7776	0.5383	0.6198
Pars Orbitalis	nean introgressed	0.0113	0.0102	0.0085	0.9004	0.7523	0.8945	0.6198
Pars Triangularis	nean introgressed	0.0113	0.0156	0.0081	1.3839	0.7162	0.5860	0.6198
Pericalcarine	nean introgressed	0.0113	0.0134	0.0051	1.1842	0.4538	0.6833	0.6198
Postcentral	nean introgressed	0.0113	0.0030	0.0080	0.2692	0.7049	0.2883	0.6146
Posterior Cingulate	nean introgressed	0.0113	0.0105	0.0081	0.9312	0.7143	0.9208	0.6198
Precentral	nean introgressed	0.0113	0.0055	0.0052	0.4887	0.4633	0.2734	0.6146
Precuneus	nean introgressed	0.0113	0.0103	0.0078	0.9163	0.6916	0.9002	0.6198
Rostral Anterior Cingulate	nean introgressed	0.0113	0.0088	0.0063	0.7771	0.5545	0.6896	0.6198
Rostral Middle Frontal	nean introgressed	0.0113	0.0149	0.0070	1.3211	0.6159	0.5990	0.6198
Superior Frontal	nean introgressed	0.0113	0.0015	0.0049	0.1353	0.4307	0.0520	0.2873
Superior Parietal	nean introgressed	0.0113	0.0091	0.0077	0.8032	0.6795	0.7709	0.6198
Superior Temporal	nean introgressed	0.0113	0.0106	0.0060	0.9384	0.5320	0.9020	0.6198
Supramarginal	nean introgressed	0.0113	0.0018	0.0087	0.1616	0.7709	0.2620	0.6146
Transverse Temporal	nean introgressed	0.0113	0.0145	0.0052	1.2835	0.4568	0.5326	0.6198

Table S8 LDSC partitioned heritability analysis results for right-hemispheric surface area metrics. FDR column shows FDR corrected (n=43) *P*-values. fetal_hge, *fetal brain human gained enhancers*; archaic_deserts, *Archaic deserts;* nean_introgressed, *Neanderthal introgressed alleles. ** Some FDR values are smaller than Enrichment *P* as FDR correction was applied for the total number of independent traits. Significant enrichments are marked in bold.

Region	Annotation	Prop. of SNPs	Prop. of h2	Prop. of h2 SE	Enrichment	Enrichment SE	Enrichment P	FDR
Total surface area	fetal hge	0.0145	-0.0157	0.0215	-1.0873	1.4890	0.1655	0.1782
Banks of the Superior Temporal Sulcus	fetal hge	0.0145	0.0123	0.0423	0.8530	2.9251	0.9597	0.6069
Caudal Anterior Cingulate	fetal hge	0.0145	0.0497	0.0611	3.4402	4.2254	0.5699	0.4376
Caudal Middle Frontal	fetal hge	0.0145	0.0629	0.0365	4.3470	2.5230	0.1815	0.1782
**Cuneus**	**fetal hge**	**0.0145**	**0.0764**	**0.0249**	**5.2839**	**1.7217**	**0.0097**	**0.0416**
Entorhinal	fetal hge	0.0145	0.0408	0.0261	2.8243	1.8043	0.3096	0.2717
Frontal Pole	fetal hge	0.0145	-0.1770	0.2796	-12.2407	19.3385	0.3299	0.2837
Fusiform	fetal hge	0.0145	0.0720	0.0427	4.9810	2.9533	0.1824	0.1782
Inferior Parietal	fetal hge	0.0145	0.0942	0.0463	6.5133	3.1991	0.0830	0.1190
Inferior Temporal	fetal hge	0.0145	0.0185	0.0298	1.2762	2.0595	0.8934	0.5820
Insula	fetal hge	0.0145	0.0588	0.0378	4.0678	2.6122	0.2319	0.2121
**Isthmus Cingulate**	**fetal hge**	**0.0145**	**0.1050**	**0.0291**	**7.2606**	**2.0132**	**0.0013**	**0.0115**
Lateral Occipital	fetal hge	0.0145	0.0550	0.0298	3.8053	2.0642	0.1738	0.1782
Lateral Orbitofrontal	fetal hge	0.0145	0.1113	0.0482	7.6968	3.3350	0.0305	0.0700
**Lingual**	**fetal hge**	**0.0145**	**0.1145**	**0.0292**	**7.9157**	**2.0192**	**0.0008**	**0.0115**
Medial Orbitofrontal	fetal hge	0.0145	0.0799	0.0448	5.5247	3.1005	0.1364	0.1585
Middle Temporal	fetal hge	0.0145	0.0554	0.0307	3.8335	2.1224	0.1765	0.1782
Paracentral	fetal hge	0.0145	0.0686	0.0423	4.7444	2.9234	0.1655	0.1782
Parahippocampal	fetal hge	0.0145	0.0733	0.0357	5.0719	2.4709	0.0945	0.1196
Pars Opercularis	fetal hge	0.0145	0.0547	0.0384	3.7849	2.6531	0.2925	0.2620
Pars Orbitalis	fetal hge	0.0145	0.0588	0.0332	4.0689	2.2960	0.1803	0.1782
Pars Triangularis	fetal hge	0.0145	0.1013	0.0419	7.0038	2.9000	0.0318	0.0700
Pericalcarine	fetal hge	0.0145	0.0540	0.0237	3.7343	1.6383	0.0990	0.1217
Postcentral	fetal hge	0.0145	0.0859	0.0380	5.9391	2.6291	0.0629	0.0932
Posterior Cingulate	fetal hge	0.0145	0.0295	0.0358	2.0367	2.4734	0.6732	0.4991
Precentral	fetal hge	0.0145	0.0565	0.0253	3.9051	1.7509	0.0942	0.1196
Precuneus	fetal hge	0.0145	0.0620	0.0201	4.2902	1.3892	0.0170	0.0519
Rostral Anterior Cingulate	fetal hge	0.0145	-0.0146	0.0674	-1.0101	4.6619	0.6573	0.4958
Rostral Middle Frontal	fetal hge	0.0145	0.0543	0.0253	3.7576	1.7466	0.1036	0.1237
Superior Frontal	fetal hge	0.0145	0.1190	0.0447	8.2307	3.0934	0.0181	0.0519
Superior Parietal	fetal hge	0.0145	0.0762	0.0319	5.2710	2.2085	0.0545	0.0868
Superior Temporal	fetal hge	0.0145	0.0467	0.0253	3.2261	1.7502	0.1930	0.1805

**Supramarginal**	**fetal hge**	**0.0145**	**0.1395**	**0.0441**	**9.6460**	**3.0504**	**0.0020**	**0.0142**
Transverse Temporal	fetal hge	0.0145	0.0285	0.0344	1.9698	2.3820	0.6861	0.5000
**Total surface area**	**archaic deserts**	**0.0270**	**0.0155**	**0.0029**	**0.5747**	**0.1067**	**0.0002**	**0.0050**
Banks of the Superior Temporal Sulcus	archaic deserts	0.0270	0.0342	0.0225	1.2634	0.8330	0.7484	0.6117
Caudal Anterior Cingulate	archaic deserts	0.0270	0.0304	0.0152	1.1259	0.5613	0.8214	0.6117
Caudal Middle Frontal	archaic deserts	0.0270	0.0336	0.0079	1.2417	0.2907	0.3865	0.6044
Cuneus	archaic deserts	0.0270	0.0215	0.0071	0.7946	0.2634	0.4349	0.6044
Entorhinal	archaic deserts	0.0270	0.0233	0.0077	0.8624	0.2851	0.6341	0.6117
Frontal Pole	archaic deserts	0.0270	0.1318	0.1277	4.8740	4.7230	0.0639	0.2749
Fusiform	archaic deserts	0.0270	0.0141	0.0113	0.5209	0.4190	0.2587	0.5417
Inferior Parietal	archaic deserts	0.0270	0.0257	0.0140	0.9495	0.5191	0.9223	0.6117
Inferior Temporal	archaic deserts	0.0270	0.0218	0.0063	0.8077	0.2346	0.4185	0.6044
Insula	archaic deserts	0.0270	0.0311	0.0097	1.1504	0.3586	0.6717	0.6117
Isthmus Cingulate	archaic deserts	0.0270	0.0188	0.0072	0.6944	0.2662	0.2572	0.5417
Lateral Occipital	archaic deserts	0.0270	0.0333	0.0087	1.2302	0.3204	0.4662	0.6075
Lateral Orbitofrontal	archaic deserts	0.0270	0.0478	0.0122	1.7700	0.4524	0.0472	0.2442
Lingual	archaic deserts	0.0270	0.0298	0.0074	1.1040	0.2736	0.7059	0.6117
Medial Orbitofrontal	archaic deserts	0.0270	0.0281	0.0082	1.0407	0.3025	0.8932	0.6117
Middle Temporal	archaic deserts	0.0270	0.0346	0.0070	1.2798	0.2592	0.2645	0.5417
Paracentral	archaic deserts	0.0270	0.0218	0.0078	0.8051	0.2890	0.5151	0.6117
Parahippocampal	archaic deserts	0.0270	0.0397	0.0150	1.4698	0.5565	0.3957	0.6044
Pars Opercularis	archaic deserts	0.0270	0.0271	0.0148	1.0042	0.5457	0.9938	0.6316
Pars Orbitalis	archaic deserts	0.0270	0.0270	0.0166	0.9991	0.6141	0.9989	0.6316
Pars Triangularis	archaic deserts	0.0270	0.0151	0.0104	0.5576	0.3843	0.2475	0.5417
Pericalcarine	archaic deserts	0.0270	0.0230	0.0087	0.8493	0.3227	0.6425	0.6117
Postcentral	archaic deserts	0.0270	0.0499	0.0099	1.8465	0.3647	0.0121	0.0865
Posterior Cingulate	archaic deserts	0.0270	0.0321	0.0086	1.1893	0.3169	0.5383	0.6117
Precentral	archaic deserts	0.0270	0.0450	0.0079	1.6645	0.2908	0.0117	0.0865
Precuneus	archaic deserts	0.0270	0.0171	0.0069	0.6322	0.2556	0.1528	0.4418
Rostral Anterior Cingulate	archaic deserts	0.0270	0.0390	0.0162	1.4429	0.6004	0.4401	0.6044
Rostral Middle Frontal	archaic deserts	0.0270	0.0181	0.0074	0.6700	0.2738	0.2307	0.5417
Superior Frontal	archaic deserts	0.0270	0.0362	0.0106	1.3378	0.3934	0.3806	0.6044
Superior Parietal	archaic deserts	0.0270	0.0326	0.0086	1.2078	0.3177	0.5070	0.6117
Superior Temporal	archaic deserts	0.0270	0.0252	0.0075	0.9313	0.2786	0.8049	0.6117
Supramarginal	archaic deserts	0.0270	0.0281	0.0065	1.0379	0.2409	0.8736	0.6117
Transverse Temporal	archaic deserts	0.0270	0.0451	0.0112	1.6682	0.4152	0.0841	0.3289
Total surface area	nean introgressed	0.0113	0.0068	0.0043	0.6067	0.3825	0.3026	0.6146
Banks of the Superior	nean introgressed	0.0113	-0.0007	0.0074	-0.0594	0.6579	0.1088	0.4680

Temporal Sulcus								
Caudal Anterior Cingulate	nean introgressed	0.0113	0.0220	0.0124	1.9534	1.0949	0.3509	0.6198
Caudal Middle Frontal	nean introgressed	0.0113	0.0105	0.0066	0.9299	0.5806	0.9048	0.6198
Cuneus	nean introgressed	0.0113	0.0151	0.0054	1.3367	0.4793	0.4808	0.6198
Entorhinal	nean introgressed	0.0113	0.0131	0.0072	1.1624	0.6391	0.7982	0.6198
Frontal Pole	nean introgressed	0.0113	0.0111	0.0412	0.9794	3.6533	0.9954	0.6294
Fusiform	nean introgressed	0.0113	0.0157	0.0076	1.3949	0.6767	0.5495	0.6198
Inferior Parietal	nean introgressed	0.0113	0.0099	0.0065	0.8788	0.5762	0.8330	0.6198
Inferior Temporal	nean introgressed	0.0113	0.0191	0.0062	1.6960	0.5470	0.1914	0.6146
Insula	nean introgressed	0.0113	-0.0006	0.0057	-0.0570	0.5045	0.0469	0.2873
Isthmus Cingulate	nean introgressed	0.0113	0.0016	0.0049	0.1410	0.4299	0.0448	0.2873
Lateral Occipital	nean introgressed	0.0113	0.0171	0.0073	1.5127	0.6475	0.4198	0.6198
Lateral Orbitofrontal	nean introgressed	0.0113	-0.0028	0.0073	-0.2497	0.6431	0.0535	0.2873
Lingual	nean introgressed	0.0113	0.0124	0.0055	1.1005	0.4893	0.8356	0.6198
Medial Orbitofrontal	nean introgressed	0.0113	0.0026	0.0082	0.2312	0.7268	0.3040	0.6146
Middle Temporal	nean introgressed	0.0113	0.0041	0.0059	0.3651	0.5234	0.2320	0.6146
Paracentral	nean introgressed	0.0113	0.0022	0.0063	0.1944	0.5549	0.1648	0.5906
Parahippocampal	nean introgressed	0.0113	0.0101	0.0072	0.8962	0.6348	0.8704	0.6198
Pars Opercularis	nean introgressed	0.0113	-0.0036	0.0065	-0.3170	0.5783	0.0228	0.2873
Pars Orbitalis	nean introgressed	0.0113	0.0129	0.0060	1.1420	0.5292	0.7876	0.6198
Pars Triangularis	nean introgressed	0.0113	0.0119	0.0065	1.0574	0.5777	0.9208	0.6198
Pericalcarine	nean introgressed	0.0113	0.0127	0.0052	1.1264	0.4615	0.7825	0.6198
Postcentral	nean introgressed	0.0113	0.0075	0.0060	0.6673	0.5352	0.5395	0.6198
Posterior Cingulate	nean introgressed	0.0113	0.0162	0.0060	1.4319	0.5349	0.4048	0.6198
Precentral	nean introgressed	0.0113	0.0026	0.0046	0.2336	0.4087	0.0613	0.2931
Precuneus	nean introgressed	0.0113	0.0109	0.0055	0.9620	0.4836	0.9361	0.6198
Rostral Anterior Cingulate	nean introgressed	0.0113	0.0187	0.0109	1.6585	0.9635	0.4779	0.6198
Rostral Middle Frontal	nean introgressed	0.0113	0.0075	0.0054	0.6647	0.4768	0.4828	0.6198
Superior Frontal	nean introgressed	0.0113	-0.0020	0.0065	-0.1741	0.5788	0.0508	0.2873
Superior Parietal	nean introgressed	0.0113	0.0174	0.0094	1.5444	0.8290	0.5061	0.6198
Superior Temporal	nean introgressed	0.0113	0.0079	0.0053	0.7000	0.4658	0.5281	0.6198
Supramarginal	nean introgressed	0.0113	0.0176	0.0108	1.5616	0.9565	0.5521	0.6198
Transverse Temporal	nean introgressed	0.0113	0.0167	0.0084	1.4802	0.7404	0.5100	0.6198

Table S9. Phenotype descriptions in dMRI sample.

UKBB IDP	Tract	Hemisphere	N	FA	SD
25056-2.0	Mean FA in middle cerebellar peduncle on FA skeleton	n.a	29890	0.54	0.02
25057-2.0	Mean FA in pontine crossing tract on FA skeleton	n.a	29920	0.41	0.03
25058-2.0	Mean FA in genu of corpus callosum on FA skeleton	n.a	29865	0.72	0.03
25059-2.0	Mean FA in body of corpus callosum on FA skeleton	n.a	29890	0.71	0.03
25060-2.0	Mean FA in splenium of corpus callosum on FA skeleton	n.a	29861	0.79	0.02
25061-2.0	Mean FA in fornix on FA skeleton	n.a	29924	0.43	0.09
25062-2.0	Mean FA in corticospinal tract on FA skeleton	right	29913	0.52	0.04
25063-2.0	Mean FA in corticospinal tract on FA skeleton	left	29913	0.54	0.03
25064-2.0	Mean FA in medial lemniscus on FA skeleton	right	29860	0.60	0.03
25065-2.0	Mean FA in medial lemniscus on FA skeleton	left	29860	0.59	0.03
25066-2.0	Mean FA in inferior cerebellar peduncle on FA skeleton	right	29750	0.55	0.03
25067-2.0	Mean FA in inferior cerebellar peduncle on FA skeleton	left	29750	0.55	0.03
25068-2.0	Mean FA in superior cerebellar peduncle on FA skeleton	right	29870	0.70	0.02
25069-2.0	Mean FA in superior cerebellar peduncle on FA skeleton	left	29870	0.71	0.02
25070-2.0	Mean FA in cerebral peduncle on FA skeleton	right	29899	0.71	0.02
25071-2.0	Mean FA in cerebral peduncle on FA skeleton	left	29899	0.71	0.02
25072-2.0	Mean FA in anterior limb of internal capsule on FA skeleton	right	29888	0.60	0.02
25073-2.0	Mean FA in anterior limb of internal capsule on FA skeleton	left	29888	0.59	0.02
25074-2.0	Mean FA in posterior limb of internal capsule on FA skeleton	right	29909	0.68	0.02
25075-2.0	Mean FA in posterior limb of internal capsule on FA skeleton	left	29909	0.69	0.02
25076-2.0	Mean FA in retrolenticular part of internal capsule on FA skeleton	right	29909	0.59	0.03
25077-2.0	Mean FA in retrolenticular part of internal capsule on FA skeleton	left	29909	0.61	0.03
25078-2.0	Mean FA in anterior corona radiata on FA skeleton	right	29920	0.46	0.03
25079-2.0	Mean FA in anterior corona radiata on FA skeleton	left	29920	0.45	0.03
25080-2.0	Mean FA in superior corona radiata on FA skeleton	right	29918	0.48	0.02
25081-2.0	Mean FA in superior corona radiata on FA skeleton	left	29918	0.49	0.03
25082-2.0	Mean FA in posterior corona radiata on FA skeleton	right	29877	0.49	0.03
25083-2.0	Mean FA in posterior corona radiata on FA skeleton	left	29877	0.48	0.02
25084-2.0	Mean FA in posterior thalamic radiation on FA skeleton	right	29885	0.59	0.03

25085-2.0	Mean FA in posterior thalamic radiation on FA skeleton	left	29885	0.60	0.03
25086-2.0	Mean FA in sagittal stratum on FA skeleton	right	29906	0.56	0.03
25087-2.0	Mean FA in sagittal stratum on FA skeleton	left	29906	0.57	0.03
25088-2.0	Mean FA in external capsule on FA skeleton	right	29895	0.46	0.02
25089-2.0	Mean FA in external capsule on FA skeleton	left	29895	0.47	0.02
25090-2.0	Mean FA in cingulum cingulate gyrus on FA skeleton	right	29903	0.58	0.03
25091-2.0	Mean FA in cingulum cingulate gyrus on FA skeleton	left	29903	0.62	0.03
25092-2.0	Mean FA in cingulum hippocampus on FA skeleton	right	29903	0.46	0.04
25093-2.0	Mean FA in cingulum hippocampus on FA skeleton	left	29903	0.46	0.03
25094-2.0	Mean FA in fornix cres+stria terminalis on FA skeleton	right	29903	0.52	0.03
25095-2.0	Mean FA in fornix cres+stria terminalis on FA skeleton	left	29903	0.52	0.04
25096-2.0	Mean FA in superior longitudinal fasciculus on FA skeleton	right	29899	0.52	0.03
25097-2.0	Mean FA in superior longitudinal fasciculus on FA skeleton	left	29899	0.53	0.03
25098-2.0	Mean FA in superior fronto-occipital fasciculus on FA skeleton	right	29904	0.46	0.04
25099-2.0	Mean FA in superior fronto-occipital fasciculus on FA skeleton	left	29904	0.46	0.05
25100-2.0	Mean FA in uncinate fasciculus on FA skeleton	right	29904	0.53	0.04
25101-2.0	Mean FA in uncinate fasciculus on FA skeleton	left	29904	0.51	0.04
25102-2.0	Mean FA in tapetum on FA skeleton	right	29915	0.55	0.07
25103-2.0	Mean FA in tapetum on FA skeleton	left	29915	0.58	0.07

Table S10. Univariate SNP-heritability overview for the dMRI analysis**.**

mean FA on FA skeleton					
**Tract**	**Hemisphere**	**SNP-h^2**	**SE**	**intercept**	**SE**
Anterior Corona Radiata	left	0.2615	0.0229	1.0181	0.0078
Anterior Corona Radiata	right	0.2798	0.0248	1.0145	0.008
Anterior Limb Of Internal Capsule	left	0.2411	0.0227	1.0279	0.0072
Anterior Limb Of Internal Capsule	right	0.2657	0.023	1.0189	0.0073
Body Of Corpus Callosum	NA	0.2684	0.0259	1.022	0.008
Cerebral Peduncle	left	0.2031	0.0183	1.0175	0.0068
Cerebral Peduncle	right	0.2058	0.0182	1.0183	0.0065
Cingulum Cingulate Gyrus	left	0.2854	0.0251	1.0173	0.0075
Cingulum Cingulate Gyrus	right	0.281	0.0276	1.0177	0.0082
Cingulum Hippocampus	left	0.2266	0.0206	1.0079	0.0069
Cingulum Hippocampus	right	0.2295	0.022	1.021	0.0068
Corticospinal Tract	left	0.1451	0.0204	1.012	0.0069
Corticospinal Tract	right	0.1596	0.0193	1.0121	0.0068
External Capsule	left	0.2474	0.0245	1.0091	0.0076
External Capsule	right	0.2496	0.0256	1.0122	0.0074
Fornix	NA	0.1937	0.0207	1.0078	0.0073
Fornix Cres+Stria Terminalis	left	0.2003	0.0201	1.0071	0.007
Fornix Cres+Stria Terminalis	right	0.1712	0.0212	1.0055	0.007
Genu Of Corpus Callosum	NA	0.2632	0.0239	1.0078	0.0073
Inferior Cerebellar Peduncle	left	0.2019	0.0199	1.01	0.0065
Inferior Cerebellar Peduncle	right	0.2315	0.0221	1.0036	0.007
Medial Lemniscus	left	0.1771	0.0205	1.0281	0.0069
Medial Lemniscus	right	0.1778	0.0207	1.0203	0.0069
Middle Cerebellar Peduncle	NA	0.215	0.0225	1.0251	0.0074
Pontine Crossing Tract	NA	0.1363	0.0207	1.0173	0.0068
Posterior Corona Radiata	left	0.2609	0.0234	1.0125	0.0071
Posterior Corona Radiata	right	0.2637	0.0225	1.0108	0.0065
Posterior Limb Of Internal Capsule	left	0.2735	0.0249	1.0274	0.0077
Posterior Limb Of Internal Capsule	right	0.2626	0.0227	1.0262	0.0074
Posterior Thalamic Radiation	left	0.1935	0.0225	1.0257	0.007
Posterior Thalamic Radiation	right	0.1659	0.0222	1.0287	0.0067
Retrolenticular Part Of Internal Capsule	left	0.2167	0.0221	1.0222	0.0075
Retrolenticular Part Of Internal Capsule	right	0.2435	0.0237	1.0175	0.0081
Sagittal Stratum	left	0.2347	0.026	1.0224	0.0082
Sagittal Stratum	right	0.2246	0.0234	1.0275	0.0071
Splenium Of Corpus Callosum	NA	0.2269	0.0261	1.0212	0.0082

Superior Cerebellar Peduncle	left	0.268	0.0229	1.0233	0.0094
Superior Cerebellar Peduncle	right	0.2846	0.0234	1.0316	0.0092
Superior Corona Radiata	left	0.2567	0.0232	1.023	0.0075
Superior Corona Radiata	right	0.2699	0.0214	1.0212	0.007
Superior Fronto-Occipital Fasciculus	left	0.2351	0.0227	1.0103	0.007
Superior Fronto-Occipital Fasciculus	right	0.2454	0.0227	1.0034	0.007
Superior Longitudinal Fasciculus	left	0.2624	0.0234	1.0258	0.0074
Superior Longitudinal Fasciculus	right	0.2847	0.0247	1.0274	0.0074
Tapetum	left	0.2358	0.02	1.0104	0.0075
Tapetum	right	0.225	0.0211	1.0119	0.0072
Uncinate Fasciculus	left	0.3133	0.0301	1.0101	0.0087
Uncinate Fasciculus	right	0.3059	0.0295	1.0042	0.0086

Table S11. Genetic correlations (LD score r_g) calculated between traits in dMRI dataset and respective traits downloaded from Oxford Brain Imaging Genetics Server (10), an expanded set of genome-wide association studies of brain imaging phenotypes in UK Biobank.

mean FA on FA skeleton				
**Tract**	**Hemisphere**	r_g	***SE***	***P***
Anterior Corona Radiata	left	0.9765	0.0116	<1.0E-307
Anterior Corona Radiata	right	0.9796	0.0094	<1.0E-307
Anterior Limb Of Internal Capsule	left	0.996	0.0106	<1.0E-307
Anterior Limb Of Internal Capsule	right	0.982	0.0085	<1.0E-307
Body Of Corpus Callosum	NA	0.9812	0.0101	<1.0E-307
Cerebral Peduncle	left	0.9734	0.0154	<1.0E-307
Cerebral Peduncle	right	0.97	0.0132	<1.0E-307
Cingulum Cingulate Gyrus	left	0.9867	0.0082	<1.0E-307
Cingulum Cingulate Gyrus	right	0.9869	0.0073	<1.0E-307
Cingulum Hippocampus	left	0.9725	0.0107	<1.0E-307
Cingulum Hippocampus	right	0.9813	0.0101	<1.0E-307
Corticospinal Tract	left	0.9725	0.0163	<1.0E-307
Corticospinal Tract	right	0.9588	0.0161	<1.0E-307
External Capsule	left	0.9725	0.0106	<1.0E-307
External Capsule	right	0.9722	0.0118	<1.0E-307
Fornix	NA	0.966	0.0196	<1.0E-307
Fornix Cres+Stria Terminalis	left	0.9659	0.0176	<1.0E-307
Fornix Cres+Stria Terminalis	right	0.9471	0.0227	<1.0E-307
Genu Of Corpus Callosum	NA	0.9896	0.0115	<1.0E-307
Inferior Cerebellar Peduncle	left	0.9525	0.0196	<1.0E-307
Inferior Cerebellar Peduncle	right	0.9597	0.0165	<1.0E-307
Medial Lemniscus	left	0.9863	0.014	<1.0E-307
Medial Lemniscus	right	0.9744	0.0152	<1.0E-307
Middle Cerebellar Peduncle	NA	0.9877	0.0149	<1.0E-307
Pontine Crossing Tract	NA	0.9873	0.0166	<1.0E-307
Posterior Corona Radiata	left	0.9874	0.0088	<1.0E-307
Posterior Corona Radiata	right	0.9911	0.0081	<1.0E-307
Posterior Limb Of Internal Capsule	left	0.9952	0.0086	<1.0E-307
Posterior Limb Of Internal Capsule	right	0.9832	0.0098	<1.0E-307
Posterior Thalamic Radiation	left	0.9655	0.0148	<1.0E-307
Posterior Thalamic Radiation	right	0.9861	0.0172	<1.0E-307
Retrolenticular Part Of Internal Capsule	left	0.9728	0.0106	<1.0E-307
Retrolenticular Part Of Internal Capsule	right	0.9897	0.0103	<1.0E-307
Sagittal Stratum	left	0.9827	0.0095	<1.0E-307
Sagittal Stratum	right	0.9858	0.0103	<1.0E-307
Splenium Of Corpus Callosum	NA	1.0002	0.0098	<1.0E-307
Superior Cerebellar Peduncle	left	0.9435	0.0118	<1.0E-307
Superior Cerebellar Peduncle	right	0.95	0.0109	<1.0E-307
Superior Corona Radiata	left	0.9924	0.0092	<1.0E-307
Superior Corona Radiata	right	0.9819	0.0082	<1.0E-307
Superior Fronto-Occipital Fasciculus	left	0.9785	0.0116	<1.0E-307
Superior Fronto-Occipital Fasciculus	right	0.9778	0.0092	<1.0E-307
Superior Longitudinal Fasciculus	left	0.9981	0.008	<1.0E-307

Superior Longitudinal Fasciculus	right	0.9905	0.0075	<1.0E-307
Tapetum	left	0.9622	0.0094	<1.0E-307
Tapetum	right	0.969	0.011	<1.0E-307
Uncinate Fasciculus	left	0.9809	0.0063	<1.0E-307
Uncinate Fasciculus	right	0.9782	0.0061	<1.0E-307

Table S12. LDSC partitioned heritability analysis results for white-matter tracts. FDR column shows FDR corrected (n=25) *P*-values. fetal_hge, *fetal brain human gained enhancers*; archaic_deserts, *Archaic deserts;* nean_introgressed, *Neanderthal introgressed alleles*. Some FDR values are smaller than Enrichment *P* as FDR correction was applied for the total number of independent traits. Significant enrichments are marked in bold.

Region	Hemisphere	Annotation	Prop. of SNPs	Prop. of h^2	Prop. of h^2 SE	Enrichment	Enrichment SE	Enrichment *P*	FDR
Body Of Corpus Callosum	N.A.	fetal hge	0.0145	0.0294	0.0253	2.0315	1.7466	0.5529	0.3736
Fornix	N.A.	fetal hge	0.0145	0.0524	0.0418	3.6205	2.8919	0.3498	0.2733
Genu Of Corpus Callosum	N.A.	fetal hge	0.0145	-0.0080	0.0276	-0.5503	1.9096	0.4221	0.3015
Middle Cerebellar Peduncle	N.A.	fetal hge	0.0145	0.0907	0.0364	6.2693	2.5181	0.0365	0.1015
Pontine Crossing	N.A.	fetal hge	0.0145	0.0236	0.0408	1.6310	2.8243	0.8224	0.4284
Splenium Of Corpus Callosum	N.A.	fetal hge	0.0145	0.0612	0.0350	4.2336	2.4215	0.1828	0.1692
Body Of Corpus Callosum	N.A.	archaic deserts	0.0270	0.0170	0.0076	0.6303	0.2803	0.1827	0.4063
Fornix	N.A.	archaic deserts	0.0270	0.0150	0.0088	0.5545	0.3243	0.1817	0.4063
Genu Of Corpus Callosum	N.A.	archaic deserts	0.0270	0.0227	0.0089	0.8382	0.3282	0.6192	0.4691
Middle Cerebellar Peduncle	N.A.	archaic deserts	0.0270	0.0190	0.0079	0.7042	0.2912	0.3116	0.4063
Pontine Crossing	N.A.	archaic deserts	0.0270	0.0284	0.0084	1.0507	0.3111	0.8689	0.4731
Splenium Of Corpus Callosum	N.A.	archaic deserts	0.0270	0.0187	0.0089	0.6932	0.3290	0.3515	0.4063
Body Of Corpus Callosum	N.A.	nean introgressed	0.0113	0.0040	0.0044	0.3574	0.3939	0.1084	0.3673
Fornix	N.A.	nean introgressed	0.0113	0.0016	0.0080	0.1461	0.7093	0.2243	0.3673
Genu Of Corpus Callosum	N.A.	nean introgressed	0.0113	0.0047	0.0049	0.4184	0.4384	0.1906	0.3673
Middle Cerebellar Peduncle	N.A.	nean introgressed	0.0113	0.0178	0.0067	1.5773	0.5918	0.3150	0.3750
Pontine Crossing	N.A.	nean introgressed	0.0113	0.0184	0.0073	1.6307	0.6449	0.3134	0.3750
Splenium Of Corpus Callosum	N.A.	nean introgressed	0.0113	-0.0029	0.0063	-0.2547	0.5542	0.0212	0.1769
Anterior Corona Radiata	left	nean introgressed	0.0113	0.0105	0.0059	0.9297	0.5229	0.8930	0.4651
Anterior Limb Of Internal Capsule	left	nean introgressed	0.0113	0.0082	0.0052	0.7225	0.4592	0.5460	0.4265

Cerebral Peduncle	left	nean introgressed	0.0113	0.0071	0.0050	0.6308	0.4468	0.4142	0.3829
Cingulum Cingulate Gyrus	left	nean introgressed	0.0113	0.0071	0.0039	0.6253	0.3497	0.2851	0.3750
Cingulum Hippocampus	left	nean introgressed	0.0113	0.0052	0.0054	0.4587	0.4757	0.2578	0.3673
Corticospinal Tract	left	nean introgressed	0.0113	0.0149	0.0078	1.3216	0.6914	0.6373	0.4306
External Capsule	left	nean introgressed	0.0113	0.0059	0.0045	0.5219	0.4030	0.2385	0.3673
Fornix Cres+Stria Terminalis	left	nean introgressed	0.0113	0.0138	0.0083	1.2243	0.7343	0.7568	0.4400
Inferior Cerebellar Peduncle	left	nean introgressed	0.0113	0.0146	0.0067	1.2946	0.5898	0.6142	0.4265
Medial Lemniscus	left	nean introgressed	0.0113	0.0106	0.0043	0.9373	0.3848	0.8713	0.4634
Posterior Corona Radiata	left	nean introgressed	0.0113	0.0082	0.0045	0.7237	0.3958	0.4896	0.4080
Posterior Limb Of Internal Capsule	left	nean introgressed	0.0113	0.0067	0.0047	0.5954	0.4199	0.3353	0.3772
Posterior Thalamic Radiation	left	nean introgressed	0.0113	0.0151	0.0092	1.3410	0.8112	0.6704	0.4383
Retrolenticular Part Of Internal Capsule	left	nean introgressed	0.0113	0.0026	0.0054	0.2329	0.4751	0.0955	0.3673
Sagittal Stratum	left	nean introgressed	0.0113	0.0070	0.0054	0.6195	0.4796	0.4289	0.3829
Superior Cerebellar Peduncle	left	nean introgressed	0.0113	0.0056	0.0051	0.4962	0.4497	0.2645	0.3673
Superior Corona Radiata	left	nean introgressed	0.0113	0.0088	0.0045	0.7827	0.4030	0.5938	0.4265
Superior Fronto-Occipital Fasciculus	left	nean introgressed	0.0113	0.0099	0.0044	0.8770	0.3938	0.7555	0.4400
Superior Longitudinal Fasciculus	left	nean introgressed	0.0113	0.0096	0.0047	0.8488	0.4157	0.7173	0.4383
Tapetum	left	nean introgressed	0.0113	0.0144	0.0052	1.2790	0.4594	0.5408	0.4265
**Uncinate Fasciculus**	**left**	**nean introgressed**	**0.0113**	**0.0001**	**0.0031**	**0.0064**	**0.2764**	**0.0009**	**0.0219**
Anterior Corona Radiata	left	archaic deserts	0.0270	0.0217	0.0064	0.8022	0.2353	0.4030	0.4063
Anterior Limb Of Internal	left	archaic deserts	0.0270	0.0372	0.0101	1.3771	0.3745	0.3106	0.4063

Capsule									
Cerebral Peduncle	left	archaic deserts	0.0270	0.0327	0.0076	1.2080	0.2827	0.4551	0.4063
Cingulum Cingulate Gyrus	left	archaic deserts	0.0270	0.0293	0.0068	1.0830	0.2525	0.7413	0.4731
Cingulum Hippocampus	left	archaic deserts	0.0270	0.0216	0.0072	0.7998	0.2647	0.4530	0.4063
Corticospinal Tract	left	archaic deserts	0.0270	0.0227	0.0083	0.8413	0.3066	0.6106	0.4691
External Capsule	left	archaic deserts	0.0270	0.0187	0.0061	0.6909	0.2263	0.1775	0.4063
Fornix Cres+Stria Terminalis	left	archaic deserts	0.0270	0.0208	0.0136	0.7697	0.5028	0.6495	0.4731
Inferior Cerebellar Peduncle	left	archaic deserts	0.0270	0.0345	0.0111	1.2756	0.4092	0.4949	0.4266
Medial Lemniscus	left	archaic deserts	0.0270	0.0274	0.0056	1.0142	0.2060	0.9451	0.4922
Posterior Corona Radiata	left	archaic deserts	0.0270	0.0262	0.0049	0.9705	0.1799	0.8688	0.4731
Posterior Limb Of Internal Capsule	left	archaic deserts	0.0270	0.0308	0.0095	1.1390	0.3497	0.6906	0.4731
Posterior Thalamic Radiation	left	archaic deserts	0.0270	0.0201	0.0074	0.7450	0.2741	0.3633	0.4063
Retrolenticular Part Of Internal Capsule	left	archaic deserts	0.0270	0.0298	0.0079	1.1015	0.2924	0.7286	0.4731
Sagittal Stratum	left	archaic deserts	0.0270	0.0198	0.0055	0.7313	0.2047	0.1995	0.4063
Superior Cerebellar Peduncle	left	archaic deserts	0.0270	0.0285	0.0097	1.0534	0.3573	0.8812	0.4731
Superior Corona Radiata	left	archaic deserts	0.0270	0.0229	0.0066	0.8475	0.2442	0.5347	0.4312
Superior Fronto-Occipital Fasciculus	left	archaic deserts	0.0270	0.0186	0.0066	0.6876	0.2439	0.2026	0.4063
Superior Longitudinal Fasciculus	left	archaic deserts	0.0270	0.0179	0.0042	0.6615	0.1552	0.0348	0.4063
Tapetum	left	archaic deserts	0.0270	0.0376	0.0061	1.3915	0.2251	0.0654	0.4063
Uncinate Fasciculus	left	archaic deserts	0.0270	0.0261	0.0066	0.9658	0.2452	0.8894	0.4731
Anterior Corona Radiata	left	fetal hge	0.0145	0.0225	0.0262	1.5566	1.8096	0.7585	0.4202
Anterior Limb Of Internal Capsule	left	fetal hge	0.0145	0.0575	0.0288	3.9753	1.9889	0.1438	0.1685
Cerebral Peduncle	left	fetal hge	0.0145	0.0469	0.0334	3.2414	2.3105	0.3280	0.2646
Cingulum Cingulate Gyrus	left	fetal hge	0.0145	0.0055	0.0232	0.3776	1.6067	0.6987	0.4159
Cingulum Hippocampus	left	fetal hge	0.0145	0.0880	0.0304	6.0826	2.1022	0.0129	0.0648
Corticospinal Tract	left	fetal hge	0.0145	0.0354	0.0381	2.4495	2.6374	0.5805	0.3819
External Capsule	left	fetal hge	0.0145	0.0354	0.0244	2.4477	1.6873	0.3916	0.2920

Fornix Cres+Stria Terminalis	left	fetal hge	0.0145	0.0708	0.0414	4.8943	2.8640	0.1659	0.1685
Inferior Cerebellar Peduncle	left	fetal hge	0.0145	0.0277	0.0325	1.9123	2.2483	0.6845	0.4159
Medial Lemniscus	left	fetal hge	0.0145	0.0933	0.0285	6.4489	1.9730	0.0054	0.0648
Posterior Corona Radiata	left	fetal hge	0.0145	0.0632	0.0260	4.3686	1.7985	0.0514	0.1284
Posterior Limb Of Internal Capsule	left	fetal hge	0.0145	0.0708	0.0220	4.8973	1.5224	0.0088	0.0648
Posterior Thalamic Radiation	left	fetal hge	0.0145	0.0756	0.0364	5.2286	2.5190	0.0897	0.1601
Retrolenticular Part Of Internal Capsule	left	fetal hge	0.0145	0.0634	0.0347	4.3826	2.3981	0.1577	0.1685
Sagittal Stratum	left	fetal hge	0.0145	0.0782	0.0292	5.4055	2.0224	0.0322	0.1015
Superior Cerebellar Peduncle	left	fetal hge	0.0145	0.0795	0.0389	5.4968	2.6926	0.0866	0.1601
Superior Corona Radiata	left	fetal hge	0.0145	0.0388	0.0242	2.6854	1.6722	0.3037	0.2531
Superior Fronto-Occipital Fasciculus	left	fetal hge	0.0145	0.0629	0.0307	4.3520	2.1251	0.1102	0.1621
Superior Longitudinal Fasciculus	left	fetal hge	0.0145	0.0536	0.0228	3.7059	1.5787	0.0753	0.1574
Tapetum	left	fetal hge	0.0145	0.0400	0.0304	2.7686	2.1007	0.3971	0.2920
Uncinate Fasciculus	left	fetal hge	0.0145	0.0203	0.0179	1.4035	1.2356	0.7437	0.4202
Anterior Corona Radiata	right	nean introgressed	0.0113	0.0100	0.0055	0.8840	0.4862	0.8113	0.4507
Anterior Limb Of Internal Capsule	right	nean introgressed	0.0113	0.0053	0.0047	0.4725	0.4161	0.2054	0.3673
Cerebral Peduncle	right	nean introgressed	0.0113	0.0057	0.0045	0.5072	0.3948	0.2183	0.3673
Cingulum Cingulate Gyrus	right	nean introgressed	0.0113	0.0066	0.0037	0.5831	0.3273	0.2030	0.3673
Cingulum Hippocampus	right	nean introgressed	0.0113	0.0020	0.0054	0.1804	0.4801	0.0828	0.3673
Corticospinal Tract	right	nean introgressed	0.0113	0.0259	0.0083	2.2964	0.7386	0.0524	0.3273
External Capsule	right	nean introgressed	0.0113	0.0052	0.0046	0.4641	0.4036	0.1954	0.3673
Fornix Cres+Stria Terminalis	right	nean introgressed	0.0113	0.0134	0.0060	1.1901	0.5342	0.7187	0.4383
Inferior Cerebellar Peduncle	right	nean introgressed	0.0113	0.0096	0.0066	0.8516	0.5862	0.8004	0.4507

Medial Lemniscus	right	nean introgressed	0.0113	0.0102	0.0053	0.9059	0.4682	0.8412	0.4572
Posterior Corona Radiata	right	nean introgressed	0.0113	0.0091	0.0043	0.8034	0.3784	0.6059	0.4265
Posterior Limb Of Internal Capsule	right	nean introgressed	0.0113	0.0059	0.0043	0.5271	0.3789	0.2138	0.3673
Posterior Thalamic Radiation	right	nean introgressed	0.0113	0.0152	0.0098	1.3454	0.8657	0.6854	0.4383
Retrolenticular Part Of Internal Capsule	right	nean introgressed	0.0113	0.0067	0.0056	0.5976	0.4971	0.4190	0.3829
Sagittal Stratum	right	nean introgressed	0.0113	0.0065	0.0064	0.5749	0.5662	0.4581	0.3949
Superior Cerebellar Peduncle	right	nean introgressed	0.0113	0.0074	0.0043	0.6575	0.3838	0.3770	0.3829
Superior Corona Radiata	right	nean introgressed	0.0113	0.0092	0.0040	0.8169	0.3548	0.6081	0.4265
Superior Fronto-Occipital Fasciculus	right	nean introgressed	0.0113	0.0009	0.0045	0.0821	0.3978	0.0200	0.1769
Superior Longitudinal Fasciculus	right	nean introgressed	0.0113	0.0074	0.0044	0.6596	0.3892	0.3833	0.3829
Tapetum	right	nean introgressed	0.0113	0.0159	0.0050	1.4108	0.4421	0.3470	0.3772
Uncinate Fasciculus	right	nean introgressed	0.0113	0.0046	0.0044	0.4049	0.3883	0.1408	0.3673
Anterior Corona Radiata	right	archaic deserts	0.0270	0.0284	0.0068	1.0515	0.2499	0.8363	0.4731
Anterior Limb Of Internal Capsule	right	archaic deserts	0.0270	0.0398	0.0109	1.4718	0.4044	0.2380	0.4063
Cerebral Peduncle	right	archaic deserts	0.0270	0.0359	0.0059	1.3297	0.2172	0.1166	0.4063
Cingulum Cingulate Gyrus	right	archaic deserts	0.0270	0.0309	0.0090	1.1445	0.3325	0.6636	0.4731
Cingulum Hippocampus	right	archaic deserts	0.0270	0.0143	0.0051	0.5285	0.1901	0.0182	0.4063
Corticospinal Tract	right	archaic deserts	0.0270	0.0173	0.0093	0.6391	0.3441	0.3071	0.4063
External Capsule	right	archaic deserts	0.0270	0.0190	0.0046	0.7021	0.1717	0.0939	0.4063
Fornix Cres+Stria Terminalis	right	archaic deserts	0.0270	0.0154	0.0077	0.5689	0.2843	0.1397	0.4063
Inferior Cerebellar Peduncle	right	archaic deserts	0.0270	0.0417	0.0192	1.5420	0.7097	0.4405	0.4063
Medial Lemniscus	right	archaic deserts	0.0270	0.0256	0.0086	0.9458	0.3195	0.8650	0.4731
Posterior Corona Radiata	right	archaic deserts	0.0270	0.0262	0.0041	0.9675	0.1517	0.8307	0.4731

Posterior Limb Of Internal Capsule	right	archaic deserts	0.0270	0.0283	0.0071	1.0453	0.2629	0.8635	0.4731
Posterior Thalamic Radiation	right	archaic deserts	0.0270	0.0188	0.0097	0.6939	0.3571	0.3970	0.4063
Retrolenticular Part Of Internal Capsule	right	archaic deserts	0.0270	0.0323	0.0069	1.1937	0.2569	0.4447	0.4063
Sagittal Stratum	right	archaic deserts	0.0270	0.0162	0.0106	0.6002	0.3917	0.3059	0.4063
Superior Cerebellar Peduncle	right	archaic deserts	0.0270	0.0337	0.0090	1.2475	0.3312	0.4522	0.4063
Superior Corona Radiata	right	archaic deserts	0.0270	0.0302	0.0086	1.1160	0.3164	0.7140	0.4731
Superior Fronto-Occipital Fasciculus	right	archaic deserts	0.0270	0.0195	0.0080	0.7213	0.2971	0.3484	0.4063
Superior Longitudinal Fasciculus	right	archaic deserts	0.0270	0.0203	0.0059	0.7514	0.2181	0.2558	0.4063
Tapetum	right	archaic deserts	0.0270	0.0328	0.0092	1.2129	0.3412	0.5278	0.4312
Uncinate Fasciculus	right	archaic deserts	0.0270	0.0179	0.0043	0.6614	0.1591	0.0495	0.4063
Anterior Corona Radiata	right	fetal hge	0.0145	0.0282	0.0224	1.9477	1.5494	0.5414	0.3736
Anterior Limb Of Internal Capsule	right	fetal hge	0.0145	0.0694	0.0260	4.8026	1.7990	0.0339	0.1015
Cerebral Peduncle	right	fetal hge	0.0145	0.0289	0.0291	1.9962	2.0107	0.6189	0.3868
Cingulum Cingulate Gyrus	right	fetal hge	0.0145	0.0207	0.0211	1.4310	1.4619	0.7685	0.4202
Cingulum Hippocampus	right	fetal hge	0.0145	0.0527	0.0238	3.6442	1.6483	0.1043	0.1621
Corticospinal Tract	right	fetal hge	0.0145	0.0351	0.0408	2.4305	2.8207	0.6085	0.3868
External Capsule	right	fetal hge	0.0145	0.0509	0.0235	3.5201	1.6229	0.1176	0.1633
Fornix Cres+Stria Terminalis	right	fetal hge	0.0145	0.0615	0.0348	4.2527	2.4032	0.1753	0.1685
Inferior Cerebellar Peduncle	right	fetal hge	0.0145	0.0221	0.0314	1.5265	2.1703	0.8083	0.4284
Medial Lemniscus	right	fetal hge	0.0145	0.0891	0.0322	6.1596	2.2284	0.0197	0.0821
Posterior Corona Radiata	right	fetal hge	0.0145	0.0475	0.0245	3.2878	1.6911	0.1700	0.1685
Posterior Limb Of Internal Capsule	right	fetal hge	0.0145	0.0551	0.0247	3.8132	1.7070	0.0993	0.1621
Posterior Thalamic Radiation	right	fetal hge	0.0145	0.0886	0.0417	6.1253	2.8868	0.0756	0.1574
Retrolenticular Part Of Internal Capsule	right	fetal hge	0.0145	0.0512	0.0303	3.5394	2.0987	0.2204	0.1903
Sagittal Stratum	right	fetal hge	0.0145	0.1026	0.0306	7.0980	2.1137	0.0028	0.0648
Superior Cerebellar Peduncle	right	fetal hge	0.0145	0.0667	0.0360	4.6157	2.4921	0.1403	0.1685

Superior Corona Radiata	right	fetal hge	0.0145	0.0476	0.0223	3.2910	1.5417	0.1279	0.1683
Superior Fronto-Occipital Fasciculus	right	fetal hge	0.0145	0.0570	0.0305	3.9397	2.1067	0.1544	0.1685
Superior Longitudinal Fasciculus	right	fetal hge	0.0145	0.0678	0.0217	4.6887	1.5013	0.0130	0.0648
Tapetum	right	fetal hge	0.0145	0.0082	0.0217	0.5671	1.5003	0.7731	0.4202
Uncinate Fasciculus	right	fetal hge	0.0145	0.0410	0.0217	2.8329	1.4985	0.2207	0.1903

Table S13. Left hemisphere *pars triangularis*-associated eQTLs in chromosome 3 based on GTEx, psychENCODE and CommonMind consortium (CMC) databases. The SNP in the *ZIC4* upstream foetal brain HGE is highlighted in bold. PFC: prefrontal cortex. SVA: adjusted for 20 structural variants in SVA-type analysis. The FDR column shows *P*-values corrected for multiple testing within the three datasets.

Unique ID	Database	Tissue	Gene	Tested Allele	Risk Inc. Allele	*P*	FDR
3:147100140:A:C	PsychENCODE	Adult PFC	ZIC4	C	C	5.95E-10	1.30E-07
3:147100140:A:C	CMC	Adult PFC - SVA	ZIC4	C	C	NA	0.01
3:147100140:A:C	CMC	Adult PFC - no SVA	ZIC4	C	C	NA	0.01
3:147100140:A:C	CMC	Adult PFC - no SVA	ZIC4	C	C	NA	0.01
3:147101640:C:T	CMC	Adult PFC - SVA	ZIC4	C	C	NA	0.01
3:147101640:C:T	CMC	Adult PFC - no SVA	ZIC4	C	C	NA	0.01
3:147101640:C:T	CMC	Adult PFC - no SVA	ZIC4	C	C	NA	0.01
**3:147102874:C:T**	**PsychENCODE**	**Adult PFC**	**ZIC4**	**T**	**T**	**2.45E-11**	**6.33E-09**
**3:147102874:C:T**	**CMC**	**Adult PFC - SVA**	**ZIC4**	**T**	**T**	**NA**	**0.01**
**3:147102874:C:T**	**CMC**	**Adult PFC - no SVA**	**ZIC4**	**T**	**T**	**NA**	**0.01**
**3:147102874:C:T**	**CMC**	**Adult PFC - no SVA**	**ZIC4**	**T**	**T**	**NA**	**0.01**
3:147106319:C:T	CMC	Adult PFC - SVA	ZIC4	T	T	NA	0.01
3:147106319:C:T	CMC	Adult PFC - no SVA	ZIC4	T	T	NA	0.01
3:147106319:C:T	CMC	Adult PFC - no SVA	ZIC4	T	T	NA	0.01
3:147107606:A:G	CMC	Adult PFC - SVA	ZIC4	G	G	NA	0.01
3:147107606:A:G	CMC	Adult PFC - no SVA	ZIC4	G	G	NA	0.01
3:147107606:A:G	CMC	Adult PFC - no SVA	ZIC4	G	G	NA	0.01
3:147107665:C:T	CMC	Adult PFC - SVA	ZIC4	C	C	NA	0.01
3:147107665:C:T	CMC	Adult PFC - no SVA	ZIC4	C	C	NA	0.01
3:147107665:C:T	CMC	Adult PFC - no SVA	ZIC4	C	C	NA	0.01
3:147109829:C:T	CMC	Adult PFC - SVA	ZIC4	C	C	NA	0.01
3:147109829:C:T	CMC	Adult PFC - no SVA	ZIC4	C	C	NA	0.01
3:147109829:C:T	CMC	Adult PFC - no SVA	ZIC4	C	C	NA	0.01
3:147111779:C:T	PsychENCODE	Adult PFC	ZIC4	C	C	4.03E-10	9.02E-08
3:147111779:C:T	CMC	Adult PFC - SVA	ZIC4	C	C	NA	0.01
3:147111779:C:T	CMC	Adult PFC - no SVA	ZIC4	C	C	NA	0.01
3:147111779:C:T	CMC	Adult PFC - no SVA	ZIC4	C	C	NA	0.01
3:147113586:A:T	PsychENCODE	Adult PFC	ZIC4	T	T	6.90E-08	1.13E-05
3:147113586:A:T	CMC	Adult PFC - SVA	ZIC4	T	T	NA	0.01

3:147113586:A:T	CMC	Adult PFC - no SVA	ZIC4	T	T	NA	0.01
3:147113586:A:T	CMC	Adult PFC - no SVA	ZIC4	T	T	NA	0.01
3:147116084:A:G	CMC	Adult PFC - SVA	ZIC4	G	G	NA	0.01
3:147116084:A:G	CMC	Adult PFC - no SVA	ZIC4	G	G	NA	0.01
3:147116084:A:G	CMC	Adult PFC - no SVA	ZIC4	G	G	NA	0.01
3:147116846:A:C	PsychENCODE	Adult PFC	ZIC4	C	C	7.99E-08	1.29E-05
3:147116846:A:C	CMC	Adult PFC - SVA	ZIC4	C	C	NA	0.01
3:147116846:A:C	CMC	Adult PFC - no SVA	ZIC4	C	C	NA	0.01
3:147116846:A:C	CMC	Adult PFC - no SVA	ZIC4	C	C	NA	0.01
3:147117260:A:G	CMC	Adult PFC - SVA	ZIC4	G	G	NA	0.01
3:147117260:A:G	CMC	Adult PFC - no SVA	ZIC4	G	G	NA	0.01
3:147117260:A:G	CMC	Adult PFC - no SVA	ZIC4	G	G	NA	0.01
3:147124706:A:G	PsychENCODE	Adult PFC	ZIC4	A	A	8.86E-08	1.42E-05
3:147124706:A:G	CMC	Adult PFC - SVA	ZIC4	A	A	NA	0.01
3:147124706:A:G	CMC	Adult PFC - no SVA	ZIC4	A	A	NA	0.01
3:147124706:A:G	CMC	Adult PFC - no SVA	ZIC4	A	A	NA	0.01
3:147129057:A:AAC	CMC	Adult PFC - SVA	ZIC4	A	A	NA	0.05
3:147129057:A:AAC	CMC	Adult PFC - no SVA	ZIC4	A	A	NA	0.01
3:147129057:A:AAC	CMC	Adult PFC - no SVA	ZIC4	A	A	NA	0.01
3:147134032:A:G	CMC	Adult PFC - SVA	ZIC4	G	G	NA	0.01
3:147134032:A:G	CMC	Adult PFC - no SVA	ZIC4	G	G	NA	0.01
3:147134032:A:G	CMC	Adult PFC - no SVA	ZIC4	G	G	NA	0.01
3:147134039:A:G	CMC	Adult PFC - SVA	ZIC4	G	G	NA	0.01
3:147134039:A:G	CMC	Adult PFC - no SVA	ZIC4	G	G	NA	0.01
3:147134039:A:G	CMC	Adult PFC - no SVA	ZIC4	G	G	NA	0.01
3:147137518:A:G	PsychENCODE	Adult PFC	ZIC4	A	A	6.95E-09	1.32E-06
3:147137518:A:G	CMC	Adult PFC - SVA	ZIC4	A	A	NA	0.01
3:147137518:A:G	CMC	Adult PFC - no SVA	ZIC4	A	A	NA	0.01
3:147137518:A:G	CMC	Adult PFC - no SVA	ZIC4	A	A	NA	0.01
3:147137597:C:T	PsychENCODE	Adult PFC	ZIC4	C	C	1.82E-06	0
3:147137597:C:T	CMC	Adult PFC - SVA	ZIC4	C	C	NA	0.01
3:147137597:C:T	CMC	Adult PFC - no SVA	ZIC4	C	C	NA	0.01
3:147137597:C:T	CMC	Adult PFC - no SVA	ZIC4	C	C	NA	0.01
3:147137674:A:G	PsychENCODE	Adult PFC	ZIC4	G	G	7.47E-08	1.22E-05
3:147137674:A:G	CMC	Adult PFC - SVA	ZIC4	G	G	NA	0.01

3:147137674:A:G	CMC	Adult PFC - no SVA	ZIC4	G	G	NA	0.01
3:147137674:A:G	CMC	Adult PFC - no SVA	ZIC4	G	G	NA	0.01
3:147137976:G:T	CMC	Adult PFC - SVA	ZIC4	G	G	NA	0.01
3:147137976:G:T	CMC	Adult PFC - no SVA	ZIC4	G	G	NA	0.01
3:147137976:G:T	CMC	Adult PFC - no SVA	ZIC4	G	G	NA	0.01
3:147139132:A:G	PsychENCODE	Adult PFC	ZIC4	G	G	5.23E-09	1.01E-06
3:147139132:A:G	CMC	Adult PFC - SVA	ZIC4	G	G	NA	0.01
3:147139132:A:G	CMC	Adult PFC - no SVA	ZIC4	G	G	NA	0.01
3:147139132:A:G	CMC	Adult PFC - no SVA	ZIC4	G	G	NA	0.01
3:147140680:G:T	PsychENCODE	Adult PFC	ZIC4	G	G	7.29E-08	1.19E-05
3:147140680:G:T	CMC	Adult PFC - SVA	ZIC4	G	G	NA	0.05
3:147140680:G:T	CMC	Adult PFC - no SVA	ZIC4	G	G	NA	0.05
3:147140680:G:T	CMC	Adult PFC - no SVA	ZIC4	G	G	NA	0.05
3:147141344:C:T	CMC	Adult PFC - SVA	ZIC4	T	T	NA	0.01
3:147141344:C:T	CMC	Adult PFC - no SVA	ZIC4	T	T	NA	0.01
3:147141344:C:T	CMC	Adult PFC - no SVA	ZIC4	T	T	NA	0.01
3:147142127:A:G	PsychENCODE	Adult PFC	ZIC4	A	A	7.39E-08	1.20E-05
3:147142127:A:G	CMC	Adult PFC - SVA	ZIC4	A	A	NA	0.01
3:147142127:A:G	CMC	Adult PFC - no SVA	ZIC4	A	A	NA	0.01
3:147142127:A:G	CMC	Adult PFC - no SVA	ZIC4	A	A	NA	0.01
3:147142513:A:G	CMC	Adult PFC - SVA	ZIC4	G	G	NA	0.01
3:147142513:A:G	CMC	Adult PFC - no SVA	ZIC4	G	G	NA	0.01
3:147142513:A:G	CMC	Adult PFC - no SVA	ZIC4	G	G	NA	0.01
3:147142723:A:G	PsychENCODE	Adult PFC	ZIC1	G	G	0	0.04
3:147142723:A:G	PsychENCODE	Adult PFC	ZIC4	G	G	1.62E-07	2.50E-05
3:147142723:A:G	CMC	Adult PFC - SVA	ZIC4	G	G	NA	0.01
3:147142723:A:G	CMC	Adult PFC - no SVA	ZIC4	G	G	NA	0.01
3:147142723:A:G	CMC	Adult PFC - no SVA	ZIC4	G	G	NA	0.01
3:147146009:G:T	PsychENCODE	Adult PFC	ZIC1	T	T	0	0.03
3:147146009:G:T	PsychENCODE	Adult PFC	ZIC4	T	T	2.44E-07	3.65E-05
3:147146009:G:T	CMC	Adult PFC - SVA	ZIC4	T	T	NA	0.01
3:147146009:G:T	CMC	Adult PFC - no SVA	ZIC4	T	T	NA	0.01
3:147146009:G:T	CMC	Adult PFC - no SVA	ZIC4	T	T	NA	0.01
3:147148217:A:G	PsychENCODE	Adult PFC	ZIC4	G	G	2.38E-08	4.19E-06
3:147148217:A:G	CMC	Adult PFC - SVA	ZIC4	G	G	NA	0.01

3:147148217:A:G	CMC	Adult PFC - no SVA	ZIC4	G	G	NA	0.01
3:147148217:A:G	CMC	Adult PFC - no SVA	ZIC4	G	G	NA	0.01
3:147149477:A:T	PsychENCODE	Adult PFC	ZIC4	T	T	2.38E-08	4.19E-06
3:147149477:A:T	CMC	Adult PFC - SVA	ZIC4	T	T	NA	0.01
3:147149477:A:T	CMC	Adult PFC - no SVA	ZIC4	T	T	NA	0.01
3:147149477:A:T	CMC	Adult PFC - no SVA	ZIC4	T	T	NA	0.01
3:147153599:G:T	PsychENCODE	Adult PFC	ZIC1	T	T	0	0.03
3:147153599:G:T	PsychENCODE	Adult PFC	ZIC4	T	T	1.88E-07	2.86E-05
3:147153599:G:T	CMC	Adult PFC - SVA	ZIC4	T	T	NA	0.01
3:147153599:G:T	CMC	Adult PFC - no SVA	ZIC4	T	T	NA	0.01
3:147153599:G:T	CMC	Adult PFC - no SVA	ZIC4	T	T	NA	0.01
3:147153885:G:T	PsychENCODE	Adult PFC	ZIC4	G	G	1.19E-09	2.51E-07
3:147153885:G:T	CMC	Adult PFC - SVA	ZIC4	G	G	NA	0.01
3:147153885:G:T	CMC	Adult PFC - no SVA	ZIC4	G	G	NA	0.01
3:147153885:G:T	CMC	Adult PFC - no SVA	ZIC4	G	G	NA	0.01
3:147153885:G:T	GTEx/v8	Brain_Putamen_basal_ganglia	ZIC4	G	G	1.64E-05	0.04
3:147154084:A:G	PsychENCODE	Adult PFC	ZIC4	G	G	3.20E-09	6.37E-07
3:147154084:A:G	CMC	Adult PFC - SVA	ZIC4	G	G	NA	0.01
3:147154084:A:G	CMC	Adult PFC - no SVA	ZIC4	G	G	NA	0.01
3:147154084:A:G	CMC	Adult PFC - no SVA	ZIC4	G	G	NA	0.01
3:147160171:A:G	PsychENCODE	Adult PFC	ZIC1	A	A	0	0.03
3:147160171:A:G	PsychENCODE	Adult PFC	ZIC4	A	A	1.72E-07	2.64E-05
3:147160171:A:G	CMC	Adult PFC - SVA	ZIC4	A	A	NA	0.01
3:147160171:A:G	CMC	Adult PFC - no SVA	ZIC4	A	A	NA	0.01
3:147160171:A:G	CMC	Adult PFC - no SVA	ZIC4	A	A	NA	0.01
3:147161075:A:G	CMC	Adult PFC - SVA	ZIC4	G	G	NA	0.01
3:147161075:A:G	CMC	Adult PFC - no SVA	ZIC4	G	G	NA	0.01
3:147161075:A:G	CMC	Adult PFC - no SVA	ZIC4	G	G	NA	0.01
3:147163128:G:T	PsychENCODE	Adult PFC	ZIC4	T	T	2.24E-09	4.55E-07
3:147163128:G:T	CMC	Adult PFC - SVA	ZIC4	T	T	NA	0.01
3:147163128:G:T	CMC	Adult PFC - no SVA	ZIC4	T	T	NA	0.01
3:147163128:G:T	CMC	Adult PFC - no SVA	ZIC4	T	T	NA	0.01
3:147163128:G:T	GTEx/v8	Brain_Putamen_basal_ganglia	ZIC4	T	T	2.96E-05	0.04

3:147163325:C:T	PsychENCODE	Adult PFC	ZIC1	C	C	0	0.03
3:147163325:C:T	PsychENCODE	Adult PFC	ZIC4	C	C	2.89E-07	4.27E-05
3:147163325:C:T	CMC	Adult PFC - SVA	ZIC4	C	C	NA	0.01
3:147163325:C:T	CMC	Adult PFC - no SVA	ZIC4	C	C	NA	0.01
3:147163325:C:T	CMC	Adult PFC - no SVA	ZIC4	C	C	NA	0.01
3:147163978:A:G	PsychENCODE	Adult PFC	ZIC4	A	A	3.08E-09	6.15E-07
3:147163978:A:G	CMC	Adult PFC - SVA	ZIC4	A	A	NA	0.01
3:147163978:A:G	CMC	Adult PFC - no SVA	ZIC4	A	A	NA	0.01
3:147163978:A:G	CMC	Adult PFC - no SVA	ZIC4	A	A	NA	0.01
3:147163978:A:G	GTEx/v8	Brain_Putamen_basal_ganglia	ZIC4	A	A	2.81E-05	0.04
3:147164159:C:T	PsychENCODE	Adult PFC	ZIC4	C	C	2.66E-09	5.35E-07
3:147164159:C:T	CMC	Adult PFC - SVA	ZIC4	C	C	NA	0.01
3:147164159:C:T	CMC	Adult PFC - no SVA	ZIC4	C	C	NA	0.01
3:147164159:C:T	CMC	Adult PFC - no SVA	ZIC4	C	C	NA	0.01
3:147164766:A:T	PsychENCODE	Adult PFC	ZIC1	A	A	0	0.03
3:147164766:A:T	PsychENCODE	Adult PFC	ZIC4	A	A	6.97E-07	9.62E-05
3:147164766:A:T	CMC	Adult PFC - SVA	ZIC4	A	A	NA	0.01
3:147164766:A:T	CMC	Adult PFC - no SVA	ZIC4	A	A	NA	0.01
3:147164766:A:T	CMC	Adult PFC - no SVA	ZIC4	A	A	NA	0.01
3:147164899:G:GT	CMC	Adult PFC - SVA	ZIC4	GT	GT	NA	0.01
3:147164899:G:GT	CMC	Adult PFC - no SVA	ZIC4	GT	GT	NA	0.01
3:147164899:G:GT	CMC	Adult PFC - no SVA	ZIC4	GT	GT	NA	0.01
3:147165368:A:G	PsychENCODE	Adult PFC	ZIC1	A	A	0	0.03
3:147165368:A:G	PsychENCODE	Adult PFC	ZIC4	A	A	1.66E-07	2.55E-05
3:147165368:A:G	CMC	Adult PFC - SVA	ZIC4	A	A	NA	0.01
3:147165368:A:G	CMC	Adult PFC - no SVA	ZIC4	A	A	NA	0.01
3:147165368:A:G	CMC	Adult PFC - no SVA	ZIC4	A	A	NA	0.01
3:147165814:A:G	CMC	Adult PFC - SVA	ZIC4	G	G	NA	0.01
3:147165814:A:G	CMC	Adult PFC - no SVA	ZIC4	G	G	NA	0.01
3:147165814:A:G	CMC	Adult PFC - no SVA	ZIC4	G	G	NA	0.01
3:147166229:C:T	PsychENCODE	Adult PFC	ZIC1	C	C	0	0.03
3:147166229:C:T	PsychENCODE	Adult PFC	ZIC4	C	C	1.63E-07	2.51E-05
3:147166229:C:T	CMC	Adult PFC - SVA	ZIC4	C	C	NA	0.01
3:147166229:C:T	CMC	Adult PFC - no SVA	ZIC4	C	C	NA	0.01

3:147166229:C:T	CMC	Adult PFC - no SVA	ZIC4	C	C	NA	0.01
3:147169065:C:G	CMC	Adult PFC - SVA	ZIC4	G	G	NA	0.01
3:147169065:C:G	CMC	Adult PFC - no SVA	ZIC4	G	G	NA	0.01
3:147169065:C:G	CMC	Adult PFC - no SVA	ZIC4	G	G	NA	0.01
3:147169158:A:G	CMC	Adult PFC - SVA	ZIC4	G	G	NA	0.01
3:147169158:A:G	CMC	Adult PFC - no SVA	ZIC4	G	G	NA	0.01
3:147169158:A:G	CMC	Adult PFC - no SVA	ZIC4	G	G	NA	0.01
3:147170593:C:T	PsychENCODE	Adult PFC	ZIC4	C	C	8.06E-09	1.52E-06
3:147170593:C:T	CMC	Adult PFC - SVA	ZIC4	C	C	NA	0.01
3:147170593:C:T	CMC	Adult PFC - no SVA	ZIC4	C	C	NA	0.01
3:147170593:C:T	CMC	Adult PFC - no SVA	ZIC4	C	C	NA	0.01
3:147171246:G:T	PsychENCODE	Adult PFC	ZIC4	G	G	6.15E-09	1.18E-06
3:147171246:G:T	CMC	Adult PFC - SVA	ZIC4	G	G	NA	0.01
3:147171246:G:T	CMC	Adult PFC - no SVA	ZIC4	G	G	NA	0.01
3:147171246:G:T	CMC	Adult PFC - no SVA	ZIC4	G	G	NA	0.01
3:147171254:A:G	PsychENCODE	Adult PFC	ZIC4	A	A	6.14E-09	1.18E-06
3:147171254:A:G	CMC	Adult PFC - SVA	ZIC4	A	A	NA	0.01
3:147171254:A:G	CMC	Adult PFC - no SVA	ZIC4	A	A	NA	0.01
3:147171254:A:G	CMC	Adult PFC - no SVA	ZIC4	A	A	NA	0.01
3:147171592:C:T	PsychENCODE	Adult PFC	ZIC4	T	T	5.76E-08	9.54E-06
3:147171592:C:T	CMC	Adult PFC - SVA	ZIC4	T	T	NA	0.01
3:147171592:C:T	CMC	Adult PFC - no SVA	ZIC4	T	T	NA	0.01
3:147171592:C:T	CMC	Adult PFC - no SVA	ZIC4	T	T	NA	0.01
3:147172542:C:T	PsychENCODE	Adult PFC	ZIC1	T	T	0	0.03
3:147172542:C:T	PsychENCODE	Adult PFC	ZIC4	T	T	1.48E-07	2.29E-05
3:147172542:C:T	CMC	Adult PFC - SVA	ZIC4	T	T	NA	0.01
3:147172542:C:T	CMC	Adult PFC - no SVA	ZIC4	T	T	NA	0.01
3:147172542:C:T	CMC	Adult PFC - no SVA	ZIC4	T	T	NA	0.01
3:147172921:G:T	CMC	Adult PFC - SVA	ZIC4	G	G	NA	0.01
3:147172921:G:T	CMC	Adult PFC - no SVA	ZIC4	G	G	NA	0.01
3:147172921:G:T	CMC	Adult PFC - no SVA	ZIC4	G	G	NA	0.01
3:147173901:C:T	PsychENCODE	Adult PFC	ZIC1	C	C	0	0.03
3:147173901:C:T	PsychENCODE	Adult PFC	ZIC4	C	C	1.43E-07	2.22E-05
3:147173901:C:T	CMC	Adult PFC - SVA	ZIC4	C	C	NA	0.01
3:147173901:C:T	CMC	Adult PFC - no SVA	ZIC4	C	C	NA	0.01

3:147173901:C:T	CMC	Adult PFC - no SVA	ZIC4	C	C	NA	0.01
3:147174864:C:T	CMC	Adult PFC - SVA	ZIC4	C	C	NA	0.01
3:147174864:C:T	CMC	Adult PFC - no SVA	ZIC4	C	C	NA	0.01
3:147174864:C:T	CMC	Adult PFC - no SVA	ZIC4	C	C	NA	0.01
3:147175324:C:T	CMC	Adult PFC - SVA	ZIC4	C	C	NA	0.01
3:147175324:C:T	CMC	Adult PFC - no SVA	ZIC4	C	C	NA	0.01
3:147175324:C:T	CMC	Adult PFC - no SVA	ZIC4	C	C	NA	0.01
3:147175367:A:G	CMC	Adult PFC - SVA	ZIC4	G	G	NA	0.01
3:147175367:A:G	CMC	Adult PFC - no SVA	ZIC4	G	G	NA	0.01
3:147175367:A:G	CMC	Adult PFC - no SVA	ZIC4	G	G	NA	0.01
3:147178252:G:T	PsychENCODE	Adult PFC	ZIC1	G	G	0	0.03
3:147178252:G:T	PsychENCODE	Adult PFC	ZIC4	G	G	1.39E-07	2.17E-05
3:147178252:G:T	CMC	Adult PFC - SVA	ZIC4	G	G	NA	0.01
3:147178252:G:T	CMC	Adult PFC - no SVA	ZIC4	G	G	NA	0.01
3:147178252:G:T	CMC	Adult PFC - no SVA	ZIC4	G	G	NA	0.01
3:147180677:A:C	CMC	Adult PFC - SVA	ZIC4	A	A	NA	0.01
3:147180677:A:C	CMC	Adult PFC - no SVA	ZIC4	A	A	NA	0.01
3:147180677:A:C	CMC	Adult PFC - no SVA	ZIC4	A	A	NA	0.01
3:147180946:A:G	PsychENCODE	Adult PFC	ZIC1	A	A	0	0.03
3:147180946:A:G	PsychENCODE	Adult PFC	ZIC4	A	A	1.47E-07	2.28E-05
3:147180946:A:G	CMC	Adult PFC - SVA	ZIC4	A	A	NA	0.01
3:147180946:A:G	CMC	Adult PFC - no SVA	ZIC4	A	A	NA	0.01
3:147180946:A:G	CMC	Adult PFC - no SVA	ZIC4	A	A	NA	0.01
3:147187590:C:T	CMC	Adult PFC - SVA	ZIC4	T	T	NA	0.01
3:147187590:C:T	CMC	Adult PFC - no SVA	ZIC4	T	T	NA	0.01
3:147187590:C:T	CMC	Adult PFC - no SVA	ZIC4	T	T	NA	0.01
3:147188788:C:T	PsychENCODE	Adult PFC	ZIC1	T	T	0	0.02
3:147188788:C:T	PsychENCODE	Adult PFC	ZIC4	T	T	3.52E-07	5.12E-05
3:147188788:C:T	CMC	Adult PFC - SVA	ZIC4	T	T	NA	0.01
3:147188788:C:T	CMC	Adult PFC - no SVA	ZIC4	T	T	NA	0.01
3:147188788:C:T	CMC	Adult PFC - no SVA	ZIC4	T	T	NA	0.01
3:147189001:A:G	PsychENCODE	Adult PFC	ZIC4	G	G	3.14E-09	6.26E-07
3:147189001:A:G	CMC	Adult PFC - SVA	ZIC4	G	G	NA	0.01
3:147189001:A:G	CMC	Adult PFC - no SVA	ZIC4	G	G	NA	0.01
3:147189001:A:G	CMC	Adult PFC - no SVA	ZIC4	G	G	NA	0.01

3:147191972:C:G	PsychENCODE	Adult PFC	ZIC4	C	C	9.34E-09	1.74E-06
3:147191972:C:G	CMC	Adult PFC - SVA	ZIC4	C	C	NA	0.01
3:147191972:C:G	CMC	Adult PFC - no SVA	ZIC4	C	C	NA	0.01
3:147191972:C:G	CMC	Adult PFC - no SVA	ZIC4	C	C	NA	0.01
3:147194290:C:T	PsychENCODE	Adult PFC	ZIC4	C	C	3.46E-09	6.87E-07
3:147194290:C:T	CMC	Adult PFC - SVA	ZIC4	C	C	NA	0.01
3:147194290:C:T	CMC	Adult PFC - no SVA	ZIC4	C	C	NA	0.01
3:147194290:C:T	CMC	Adult PFC - no SVA	ZIC4	C	C	NA	0.01
3:147198037:A:G	CMC	Adult PFC - SVA	ZIC4	G	G	NA	0.05
3:147199132:C:T	PsychENCODE	Adult PFC	ZIC1	C	C	0	0.02
3:147199132:C:T	PsychENCODE	Adult PFC	ZIC4	C	C	8.43E-08	1.36E-05
3:147199132:C:T	CMC	Adult PFC - SVA	ZIC4	C	C	NA	0.01
3:147199132:C:T	CMC	Adult PFC - no SVA	ZIC4	C	C	NA	0.01
3:147199132:C:T	CMC	Adult PFC - no SVA	ZIC4	C	C	NA	0.01

Table S14. Comparison between PAML (17) site models M8 and M7 for the *ZIC4* coding sequence

Model	Parameters	lnL	2(lnL(M8)-lnL(M7))	df	*chi*² P-value
M8	p_0, p, q, $w_s > 1$	-10278.63	41.26	2	< 0.001
M7	p, q	-10349.03			

Table S15. Genome-wide significant ($P < 5\times10^{-8}$) SNPs that overlap with Human Accelerated Regions or Anatomically Modern Human-derived Differentially Methylated Regions. A1 allele corresponds to the effect allele, whereas A2 is the reference allele.

Region	rsID	Annotation	A1	A2	A1 frequency	Beta	Beta SE	*P*
Left Total Surface Area	rs2853928	HAR	C	A	0.6930	-0.2948	0.0544	6.187E-08
Right Total Surface Area	rs2853928	HAR	C	A	0.6930	-0.3032	0.0552	3.972E-08
Left lateralorbitofrontal	rs10230207	HAR	T	G	0.6277	-13.1610	1.3770	1.300E-21
Left lateralorbitofrontal	rs56207542	HAR	A	G	0.2714	9.2790	1.5571	2.564E-09
Left insula	rs321403	HAR	T	G	0.3129	-9.4105	1.5778	2.484E-09
Right lateralorbitofrontal	rs6965709	HAR	A	T	0.7011	-14.6480	1.8505	2.547E-15
Right lateralorbitofrontal	rs714392	HAR	G	A	0.6297	-17.2680	1.7452	4.764E-23
Right lateralorbitofrontal	rs56207542	HAR	A	G	0.2714	13.3600	1.9703	1.216E-11
Right middletemporal	rs512182	HAR	A	G	0.4967	-11.3270	1.9498	6.339E-09
Right lateraloccipital	rs76715069	HAR	C	G	0.2045	27.1790	4.7494	1.058E-08
Left Total Surface Area	rs2857594	AMH-derived DMR	G	C	0.8496	-0.4284	0.0710	1.626E-09
Right Total Surface Area	rs3130625	AMH-derived DMR	G	A	0.8482	-0.4258	0.0710	2.082E-09
Left caudalmiddlefrontal	rs888278	AMH-derived DMR	T	G	0.6252	-14.4100	2.0750	3.873E-12
Left parsorbitalis	rs1541606	AMH-derived DMR	T	G	0.5760	3.1949	0.5308	1.768E-09
Left precuneus	rs2392657	AMH-derived DMR	T	A	0.5810	-14.4880	2.4462	3.203E-09
Left middletemporal	rs7612033	AMH-derived DMR	T	C	0.7111	12.5530	2.2999	4.852E-08
Left superiorparietal	rs11707890	AMH-derived DMR	G	T	0.4367	-21.0010	3.7174	1.626E-08
Left pericalcarine	rs1064838	AMH-derived DMR	C	T	0.4697	-10.1020	1.8285	3.324E-08
Left lateralorbitofrontal	rs11409341	AMH-derived DMR	GA	G	0.5269	-7.9222	1.3476	4.178E-09
Left precentral	rs78379000	AMH-derived DMR	G	C	0.0204	57.2490	9.1232	3.541E-10
Left precuneus	rs9901694	AMH-derived DMR	T	G	0.4808	-15.1430	2.4350	5.070E-10
Left insula	rs11398477	AMH-derived DMR	CA	C	0.4959	-10.2950	1.4662	2.239E-12
Left superiorparietal	rs2419232	AMH-derived DMR	C	T	0.5070	-24.3120	3.8690	3.351E-10
Left superiorparietal	rs13382112	AMH-derived DMR	T	A	0.3695	-21.0110	3.8516	4.931E-08
Right inferiorparietal	rs7584223	AMH-derived DMR	C	T	0.1923	30.4040	5.3346	1.213E-08
Right parsorbitalis	rs6743531	AMH-derived DMR	C	G	0.6030	4.4346	0.6555	1.355E-11
Right superiorparietal	rs11707890	AMH-derived DMR	G	T	0.4367	-20.8160	3.5227	3.475E-09
Right lateraloccipital	rs357552	AMH-derived DMR	T	G	0.7121	-23.0940	4.2024	3.926E-08
Right lateraloccipital	rs76715069	AMH-derived DMR	C	G	0.2045	27.1790	4.7494	1.058E-08
Right precentral	rs78379000	AMH-derived DMR	G	C	0.0204	55.7830	9.4995	4.345E-09

Right precuneus	rs370500262	AMH-derived DMR	G	A	0.2752	-17.8950	2.8142	2.061E-10
Right insula	rs11398477	AMH-derived DMR	CA	C	0.4959	-9.5983	1.7135	2.142E-08
Right superiorparietal	rs2419232	AMH-derived DMR	C	T	0.5070	-20.9730	3.6670	1.078E-08

SI References

1. C. Bycroft, *et al.*, The UK Biobank resource with deep phenotyping and genomic data. *Nature* **562**, 203–209 (2018).
2. C. Bellenguez, *et al.*, Genome-wide association study identifies a variant in HDAC9 associated with large vessel ischemic stroke. *Nat Genet* **44**, 328–333 (2012).
3. K. L. Miller, *et al.*, Multimodal population brain imaging in the UK Biobank prospective epidemiological study. *Nat Neurosci* **19**, 1523–1536 (2016).
4. A. M. Dale, B. Fischl, M. I. Sereno, Cortical Surface-Based Analysis: I. Segmentation and Surface Reconstruction. *NeuroImage* **9**, 179–194 (1999).
5. B. Fischl, M. I. Sereno, A. M. Dale, Cortical Surface-Based Analysis: II: Inflation, Flattening, and a Surface-Based Coordinate System. *NeuroImage* **9**, 195–207 (1999).
6. F. Alfaro-Almagro, *et al.*, Image processing and Quality Control for the first 10,000 brain imaging datasets from UK Biobank. *NeuroImage* **166**, 400–424 (2018).
7. A. K. Tilot, *et al.*, The Evolutionary History of Common Genetic Variants Influencing Human Cortical Surface Area. *Cerebral Cortex* **31**, 1873–1887 (2021).
8. K. L. Grasby, *et al.*, The genetic architecture of the human cerebral cortex. *Science* **367**, eaay6690 (2020).
9. R. S. Desikan, *et al.*, An automated labeling system for subdividing the human cerebral cortex on MRI scans into gyral based regions of interest. *NeuroImage* **31**, 968–980 (2006).
10. S. M. Smith, *et al.*, An expanded set of genome-wide association studies of brain imaging phenotypes in UK Biobank. *Nat Neurosci* **24**, 737–745 (2021).
11. L. T. Elliott, *et al.*, Genome-wide association studies of brain imaging phenotypes in UK Biobank. *Nature* **562**, 210–216 (2018).
12. S. Mori, *et al.*, Stereotaxic white matter atlas based on diffusion tensor imaging in an ICBM template. *NeuroImage* **40**, 570–582 (2008).
13. S. Wakana, *et al.*, Reproducibility of quantitative tractography methods applied to cerebral white matter. *NeuroImage* **36**, 630–644 (2007).
14. S. D. Turner, qqman: an R package for visualizing GWAS results using Q-Q and manhattan plots. *Journal of Open Source Software* **3**, 731 (2018).
15. B. K. Bulik-Sullivan, *et al.*, LD Score regression distinguishes confounding from polygenicity in genome-wide association studies. *Nat Genet* **47**, 291–295 (2015).
16. R. J. Pruim, *et al.*, LocusZoom: regional visualization of genome-wide association scan results. *Bioinformatics* **26**, 2336–2337 (2010).
17. Z. Yang, PAML: a program package for phylogenetic analysis by maximum likelihood. *Bioinformatics* **13**, 555–556 (1997).
